# Supplementary material for: Impact of the Topology of Global Macroeconomic Network on the Spreading of Economic Crises
Source: PLoS One. 2011 Mar 31;6(3):e18443. doi: 10.1371/journal.pone.0018443 (PMC3069097; doi:10.1371/journal.pone.0018443)
Supplement: Figure S1 — Flow-chart of the crisis spreading process starting from a single country C. This process is repeated for every starting country to obtain the avalanche size distribution P(A). (PDF) [file pone.0018443.s001.pdf]

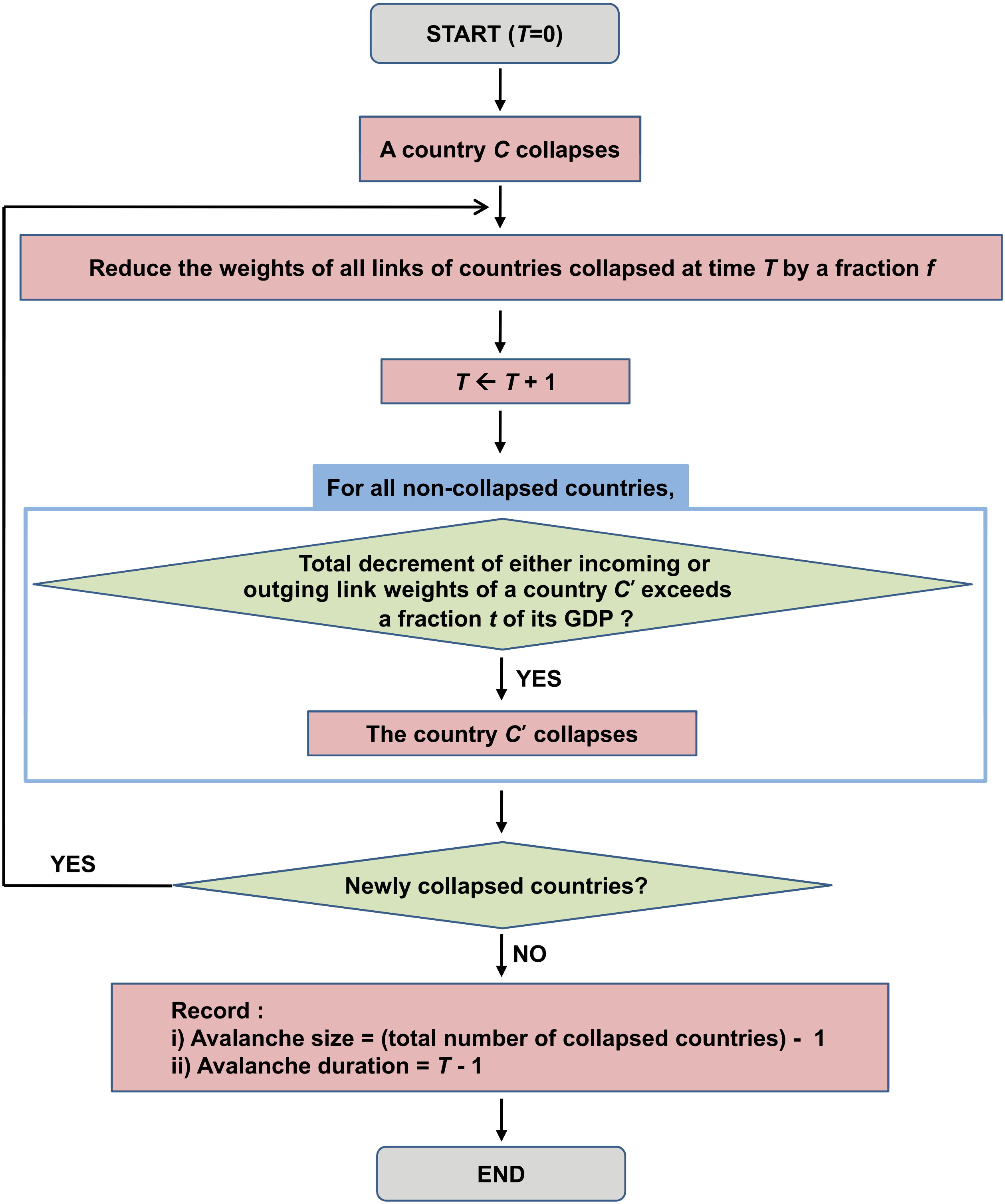
START (T=0)
A country C collapses
Reduce the weights of all links of countries collapsed at time T by a fraction f
T ← T + 1
For all non-collapsed countries,
Total decrement of either incoming or outging link weights of a country C′ exceeds a fraction t of its GDP ?
YES
The country C′ collapses
YES
Newly collapsed countries?
NO
Record :
i) Avalanche size = (total number of collapsed countries) - 1
ii) Avalanche duration = T - 1
END
