# Supplementary material for: Impact of the Topology of Global Macroeconomic Network on the Spreading of Economic Crises
Source: PLoS One. 2011 Mar 31;6(3):e18443. doi: 10.1371/journal.pone.0018443 (PMC3069097; doi:10.1371/journal.pone.0018443)

Figure S2A. Trade volume-GDP profiles of all countries (export).

Figure S2A. Trade volume-GDP profiles of all countries (export). (1/5)

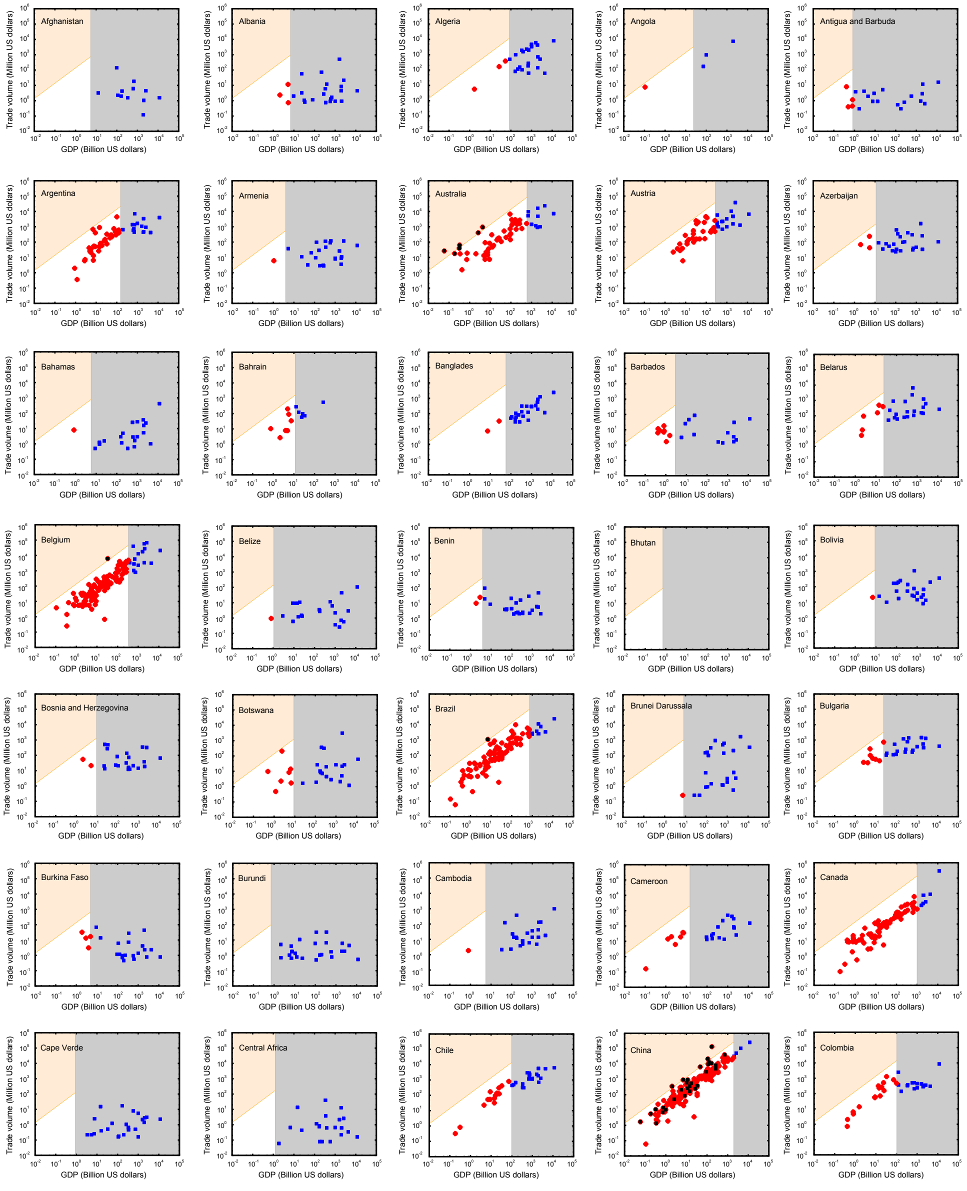

Figure S2A. Trade volume-GDP profiles of all countries (export). (2/5)

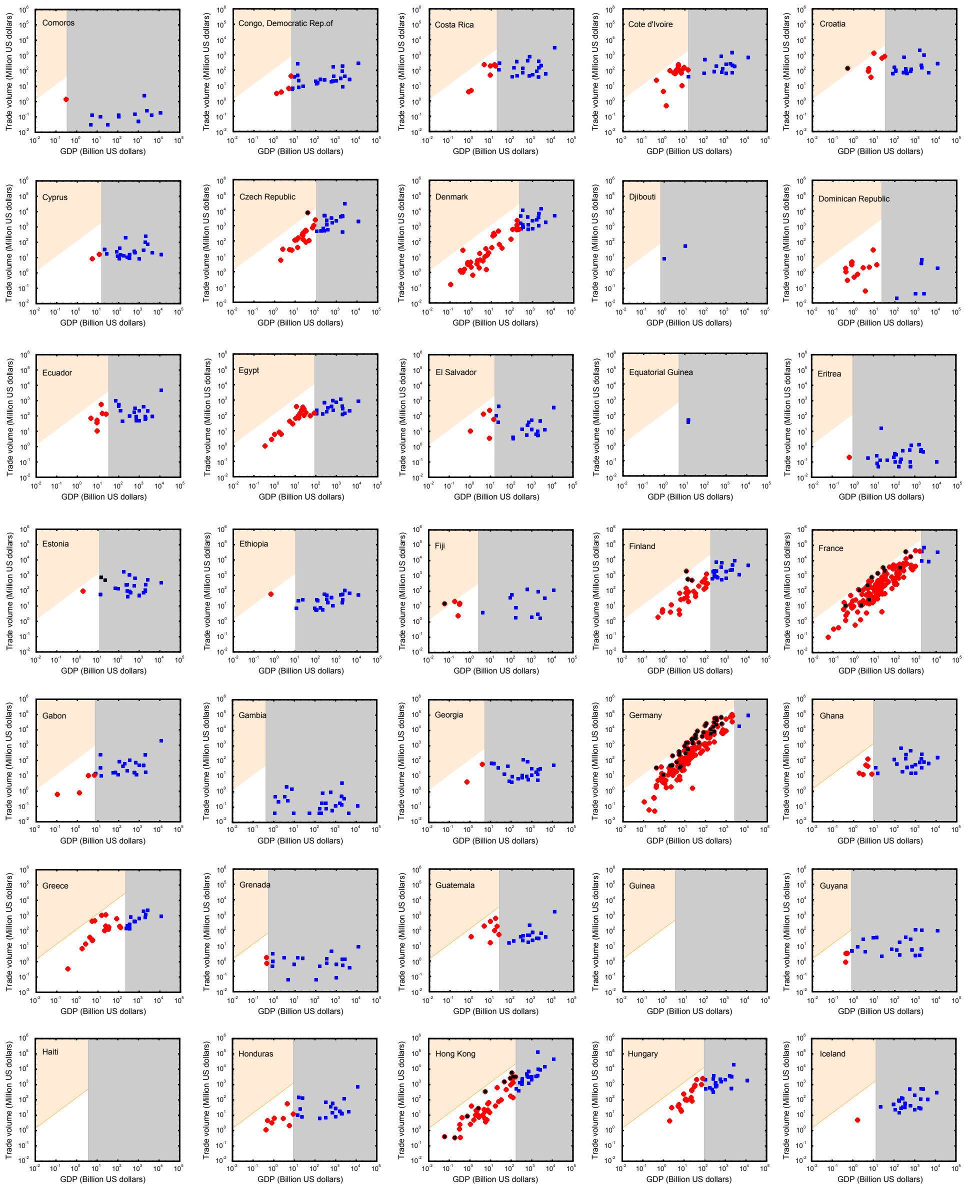

Figure S2A. Trade volume-GDP profiles of all countries (export).

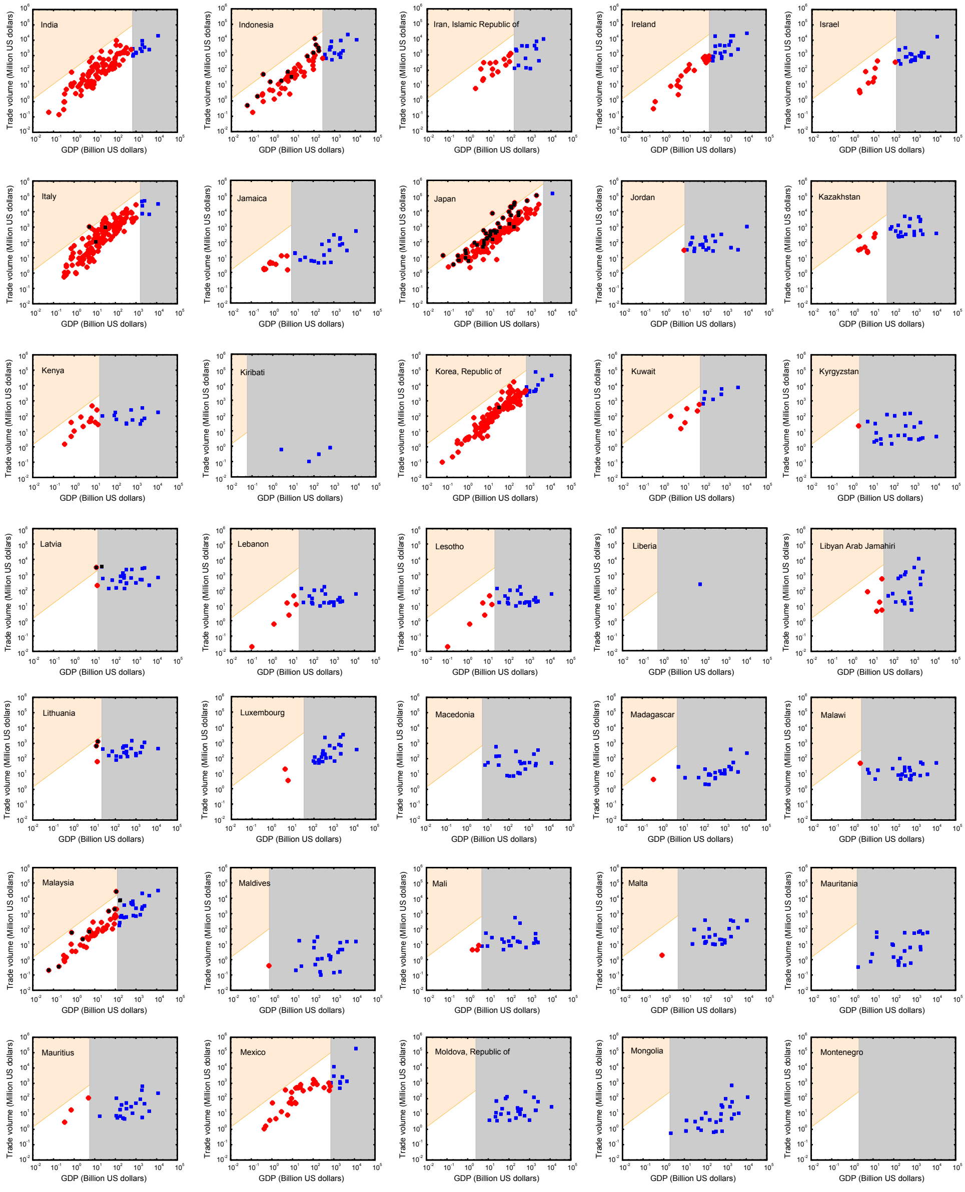

Figure S2A. Trade volume-GDP profiles of all countries (export).

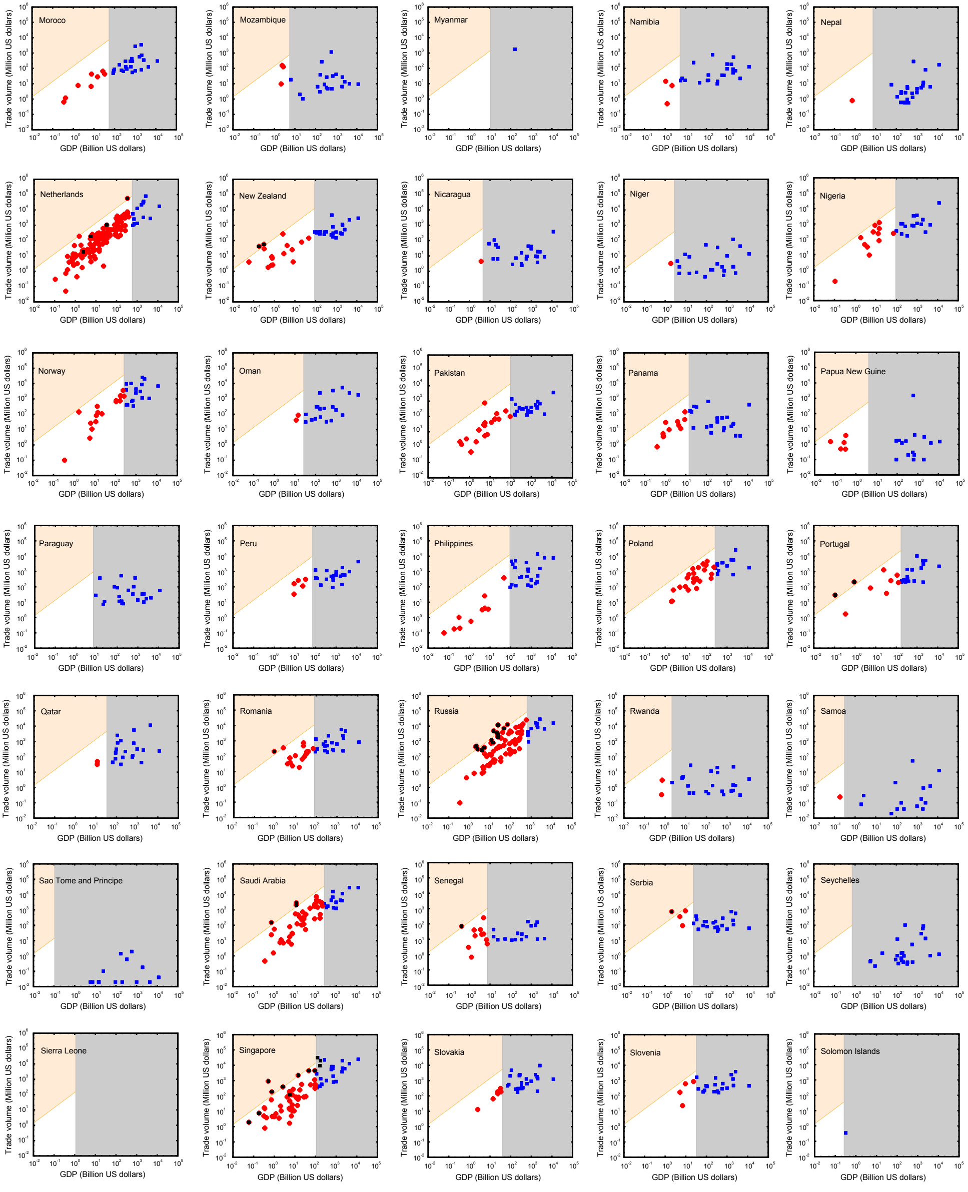

Figure S2A. Trade volume-GDP profiles of all countries (export). (5/5)

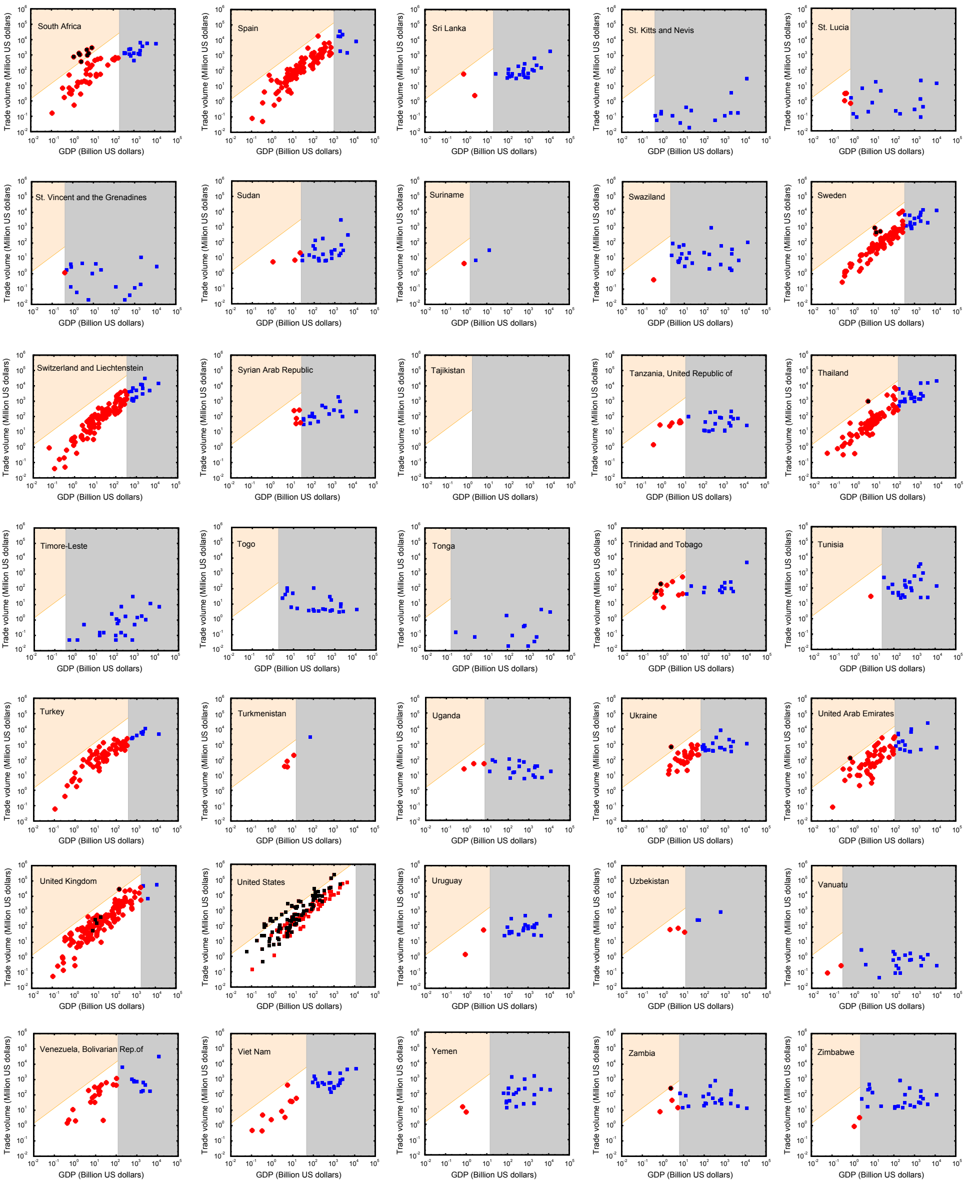

Figure S2B. Trade volume-GDP profiles of all countries (import).

Figure S2B. Trade volume-GDP profiles of all countries (import). (1/5)

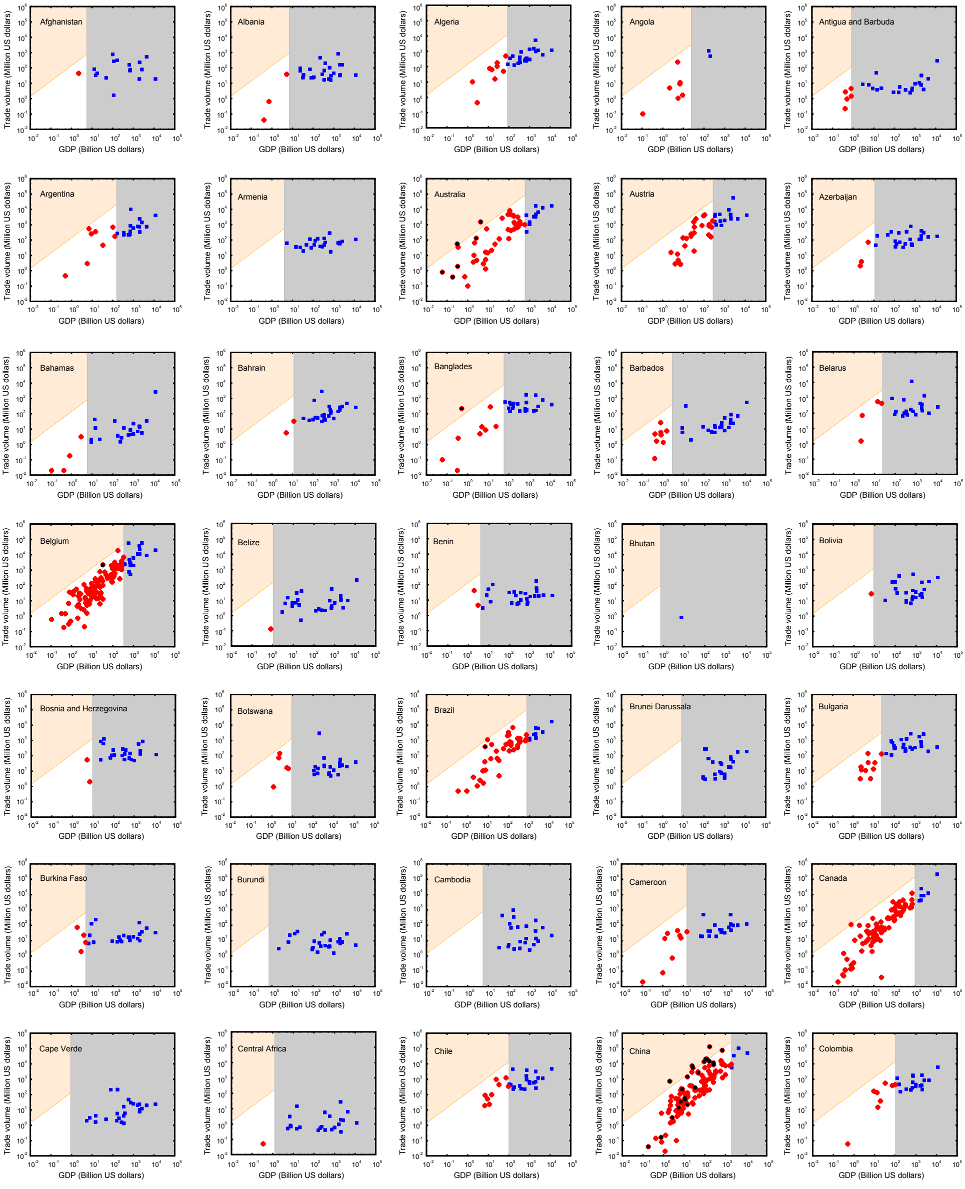

Figure S2B. Trade volume-GDP profiles of all countries (import).

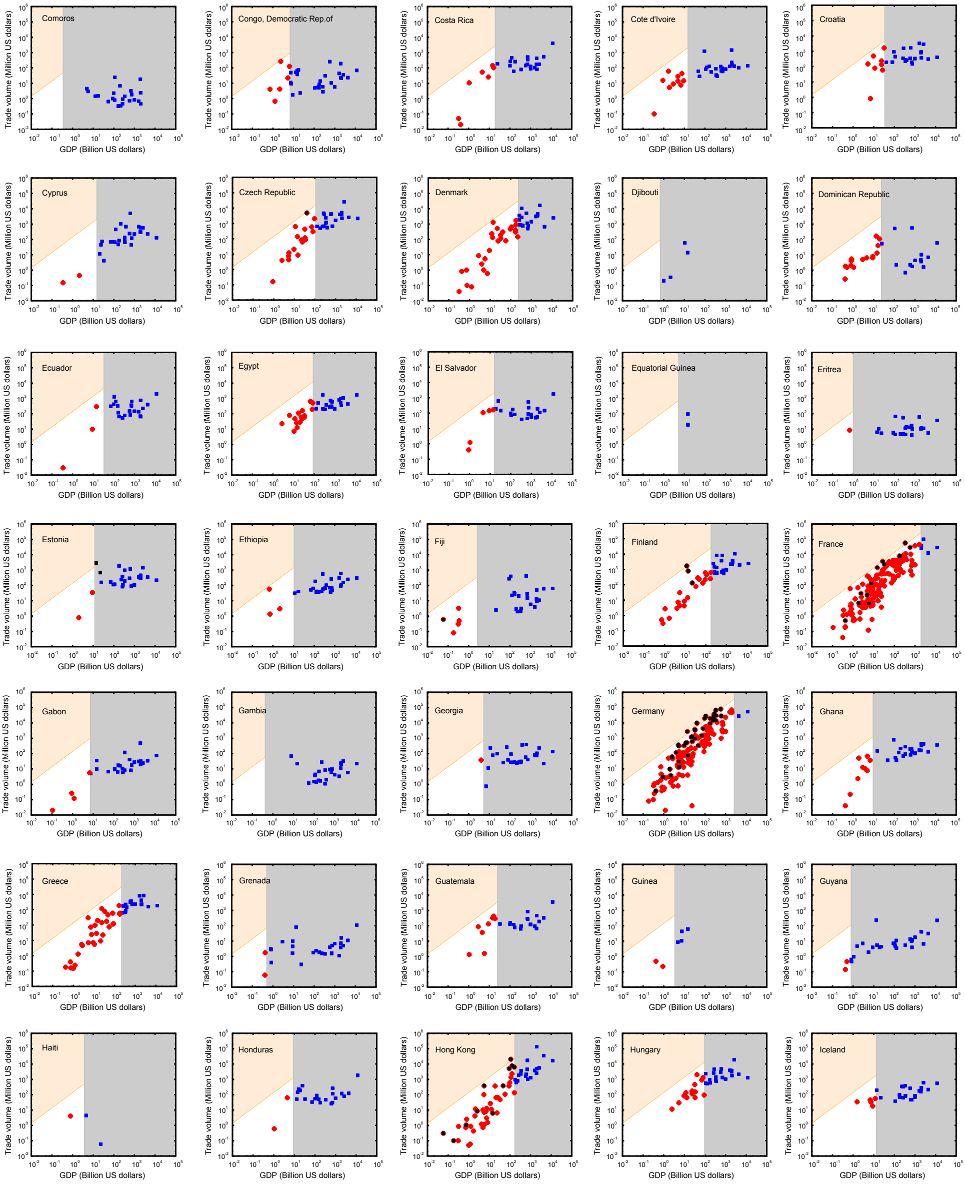

Figure S2B. Trade volume-GDP profiles of all countries (import).

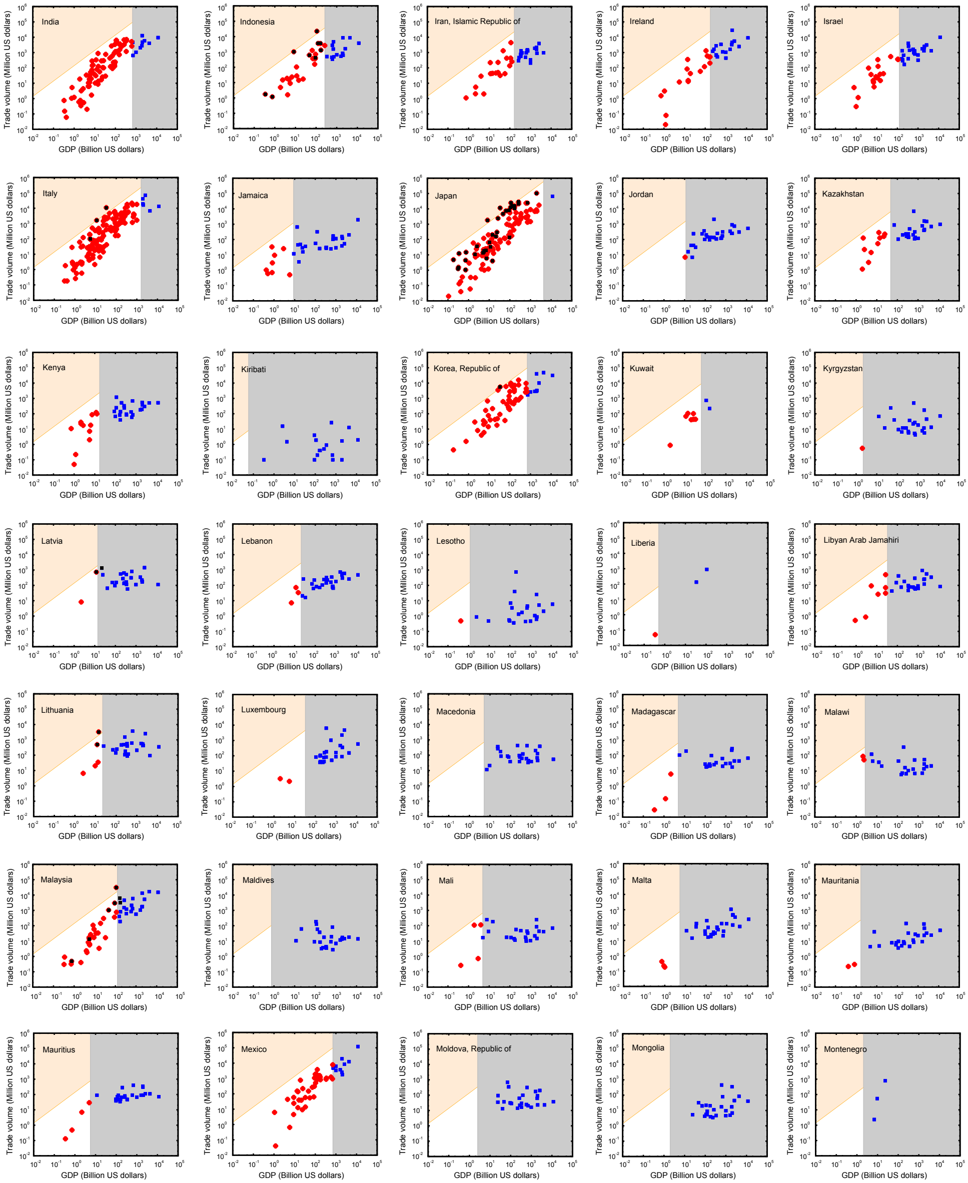

Figure S2B. Trade volume-GDP profiles of all countries (import). (4/5)

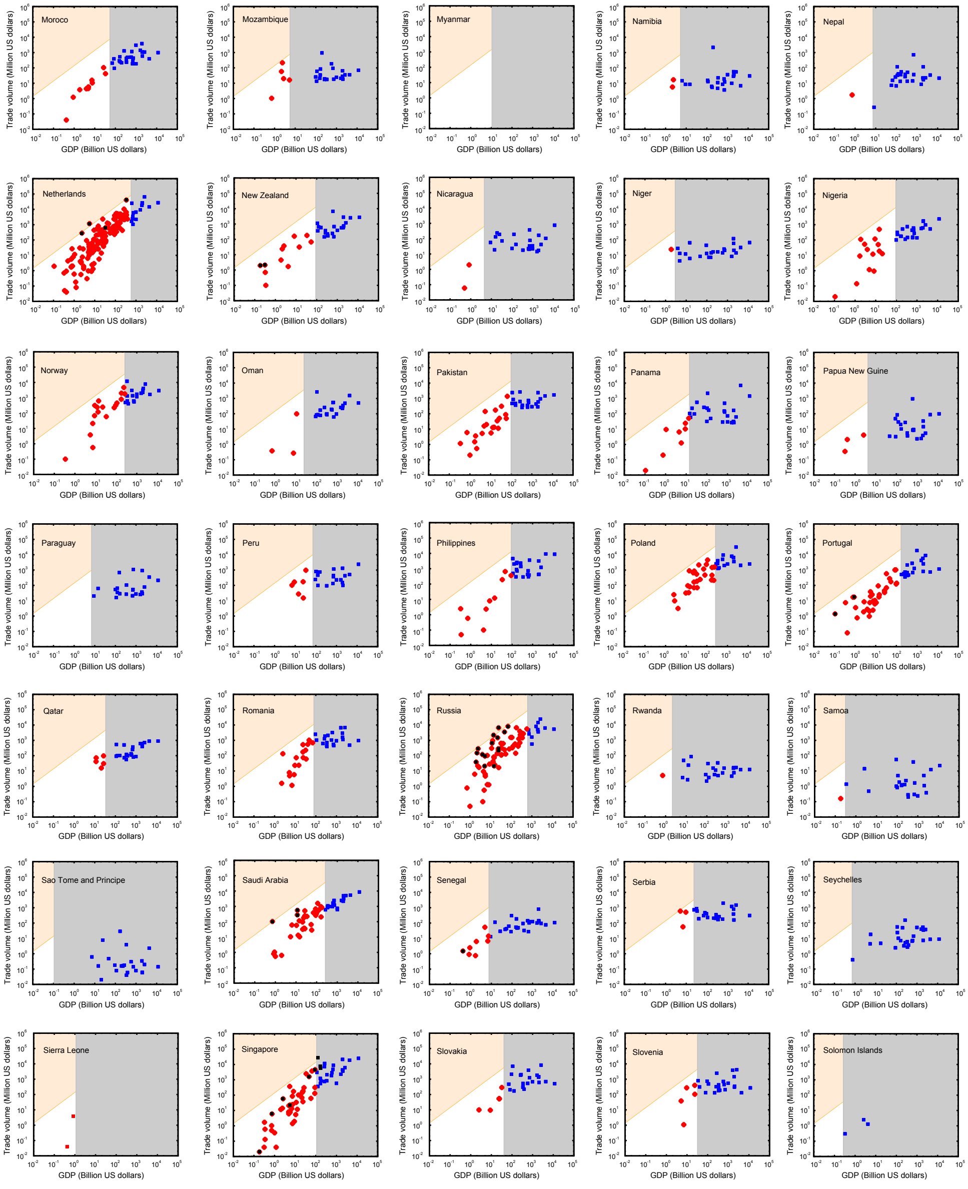

Figure S2B. Trade volume-GDP profiles of all countries (import).

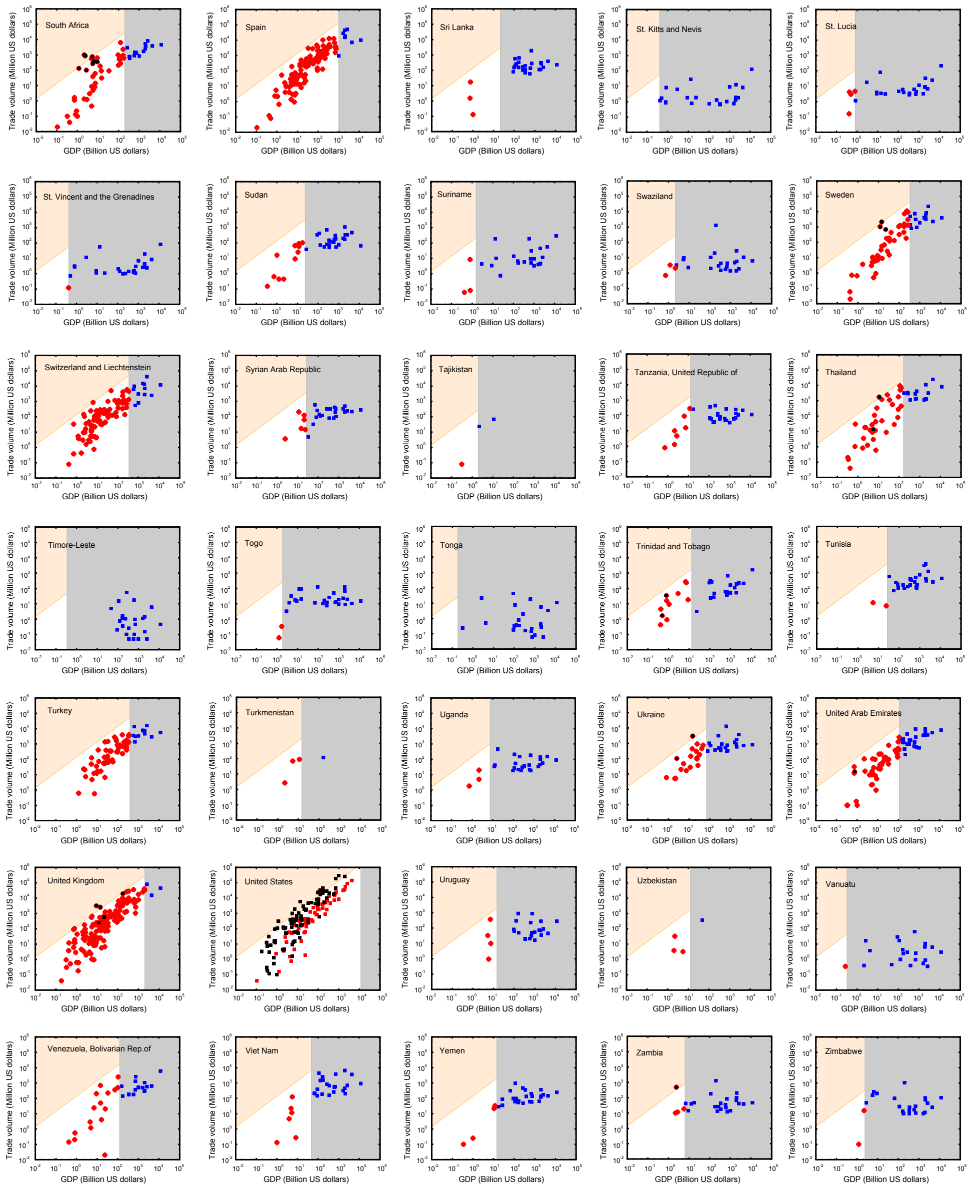

Supplement: Figure S2 — Trade volume-GDP profiles of all countries. (PDF) [file pone.0018443.s002.pdf]
