# Supplementary figures and images for: Impact of the Topology of Global Macroeconomic Network on the Spreading of Economic Crises
Source: PLoS One. 2011 Mar 31;6(3):e18443. doi: 10.1371/journal.pone.0018443 (PMC3069097; doi:10.1371/journal.pone.0018443)

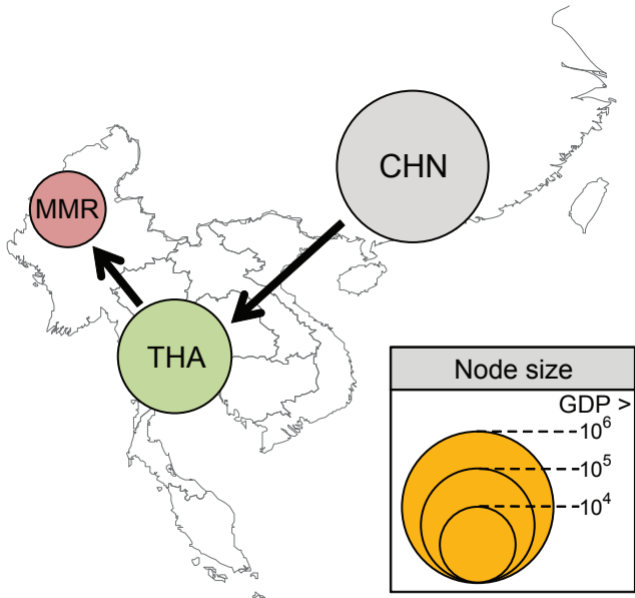

Supplement: Figure S3 — An avalanche relation between non-connected countries, China and Myanmar. (PDF) [file pone.0018443.s003.pdf]

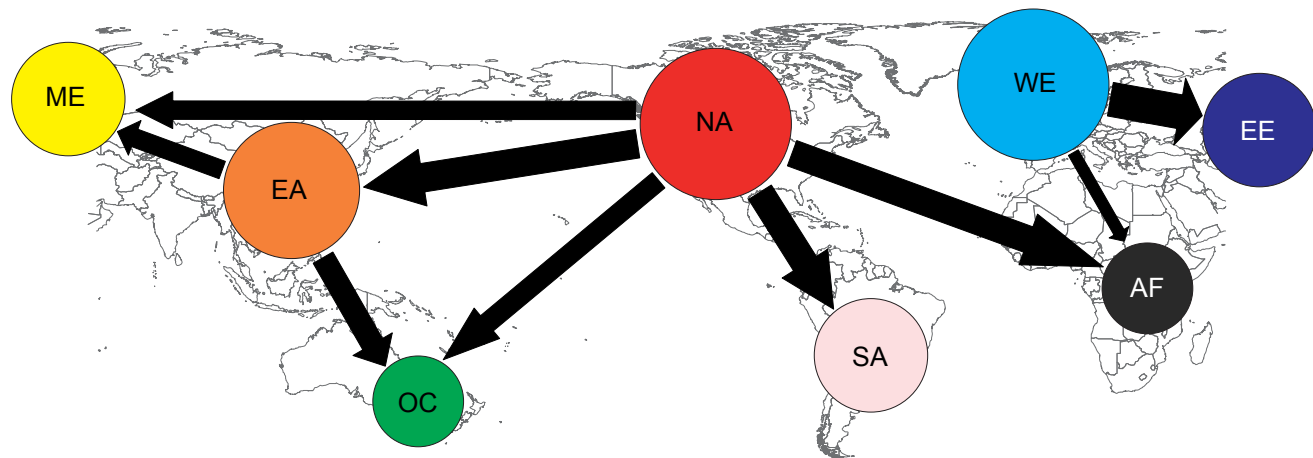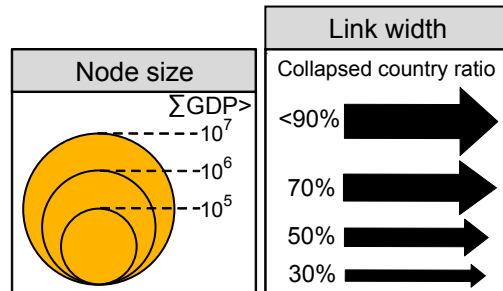

Supplement: Figure S5 — The continental avalanche network. (PDF) [file pone.0018443.s005.pdf]

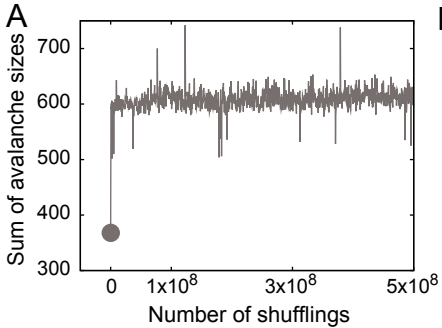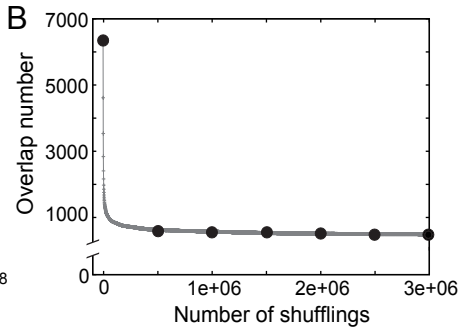

Supplement: Figure S6 — (A) Plot of the sum of avalanche sizes during the link shuffling procedure for constructing the LSN. Starting from 356 for the WEN, it quickly increased to a steady value 608±18 for the LSN. (B) Plot of the number of link overlaps between the LSN and the starting network configuration as a function of the number of random shufflings, showing the saturation after around 5×10^5^ shufflings. (PDF) [file pone.0018443.s006.pdf]

**A**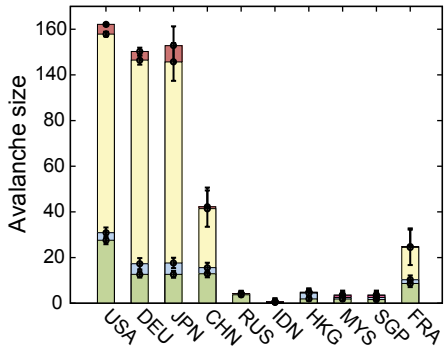**B**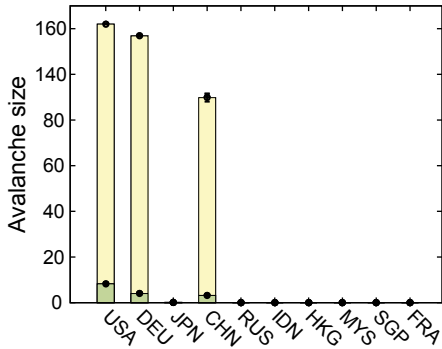

Supplement: Figure S7 — The avalanche profiles of countries with ten largest avalanche sizes in the GMN for (A) the GSN and (B) the GDN. (PDF) [file pone.0018443.s007.pdf]

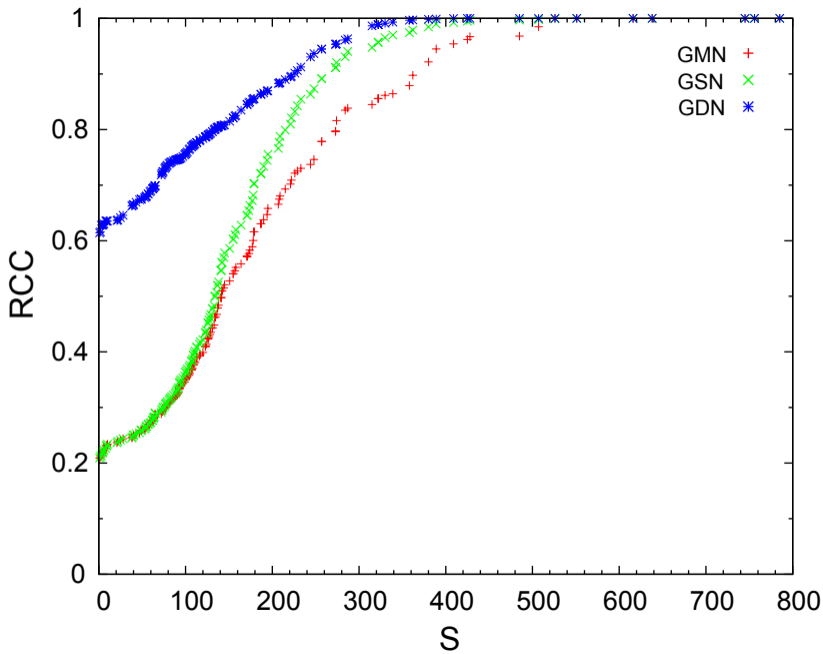

Supplement: Figure S8 — The rich-club coefficient (RCC) is plotted as a function of node strength s, the total trade volume of the country, for the original network (GMN, +) and two randomized structures, the GSN (x) and GDN (*). (PDF) [file pone.0018443.s008.pdf]

**A**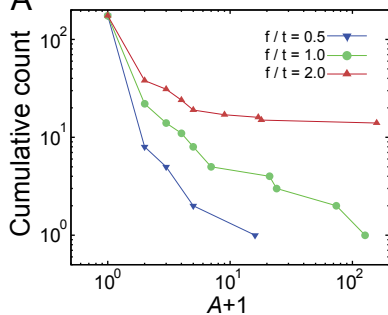**B**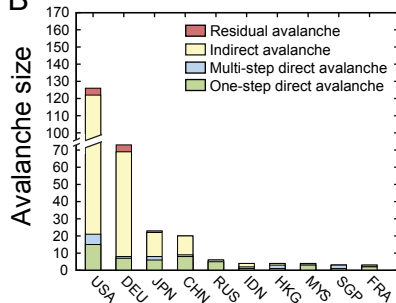**C**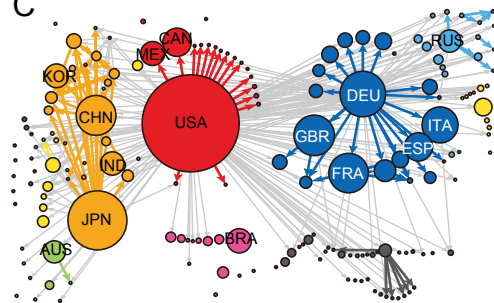**D**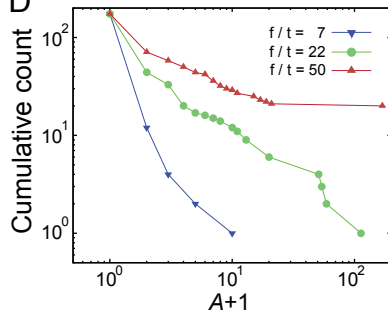**E**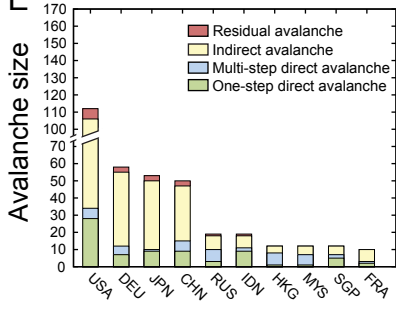**F**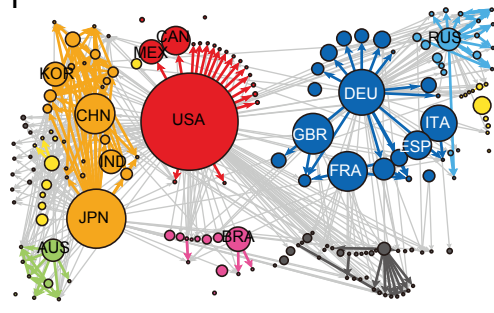**G**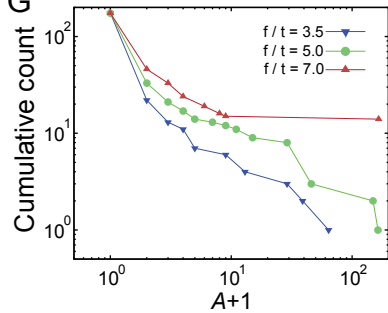**H**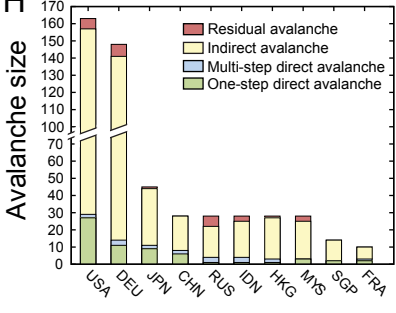**I**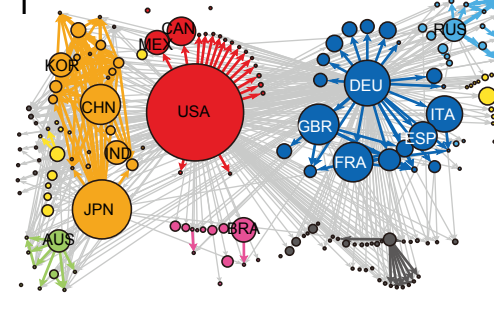

Supplement: Figure S9 — The global avalanche properties of crisis spreading models with modified rules. (A–C) The avalanche size distribution (A), avalanche profiles (B), and the avalanche network (C) of the link-weight rescaling model with α = 1.2. (D–F) Same set of plots for the node-weight rescaling model with α = 1.1. (G–I) Same set of plots for the total-trade model. We can see that the overall features of the avalanche outcomes are robust with these modifications. (PDF) [file pone.0018443.s009.pdf]

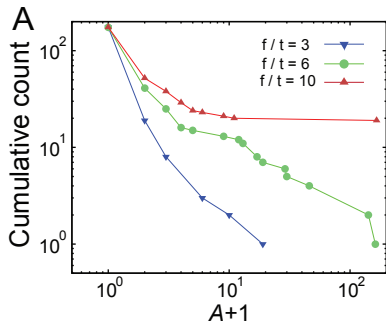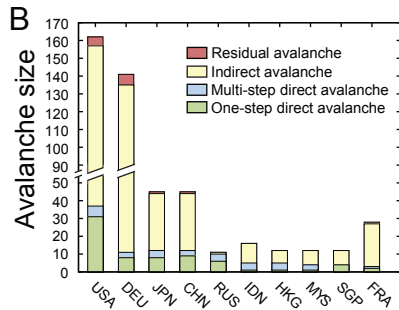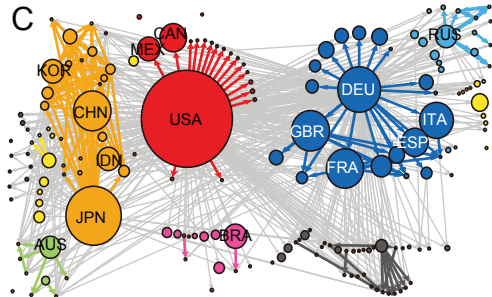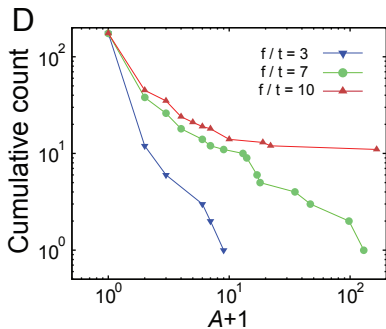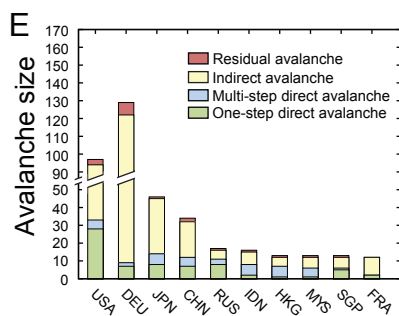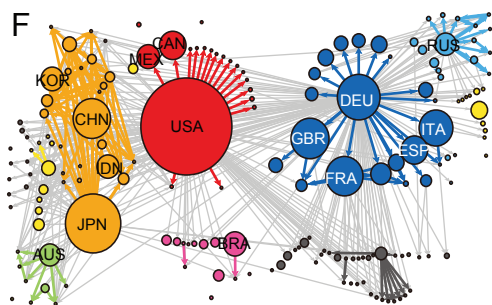

Supplement: Figure S10 — The global avalanche properties of crisis spreading models with countries' economic capacities modified by public debt (A–C), and external debt (D–F). We can see that the overall features of the avalanche outcomes are robust with these modifications. (PDF) [file pone.0018443.s010.pdf]

Cumulative count

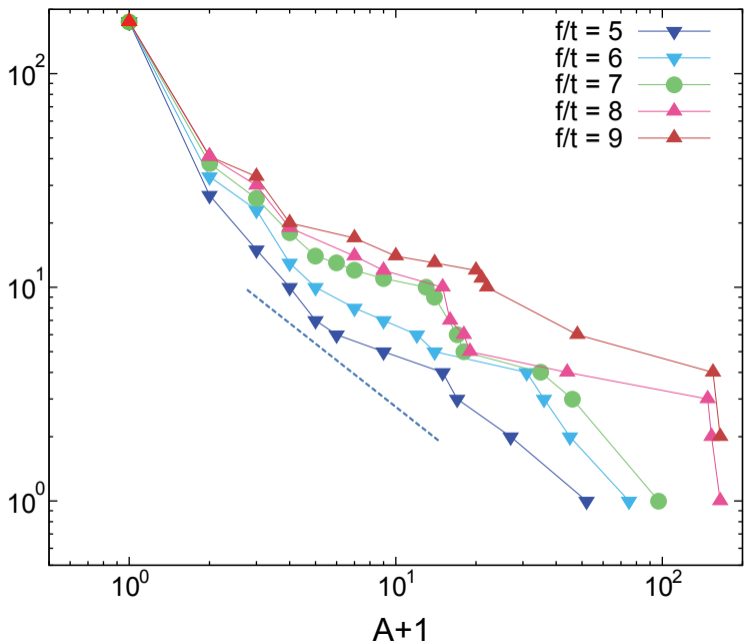

Supplement: Figure S11 — Avalanche size distributions. The power-law distribution is robust around the critical parameter f/t = 7. The dashed line has a slope of -1. (PDF) [file pone.0018443.s011.pdf]

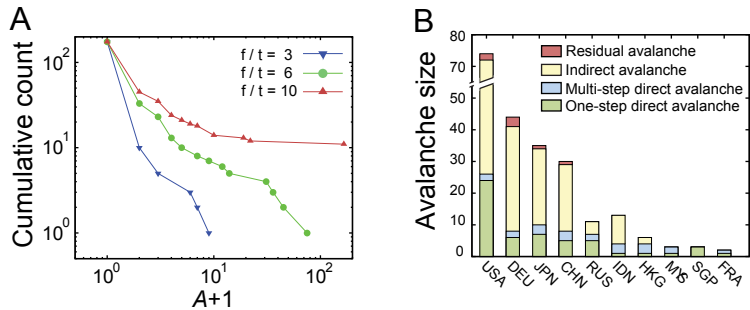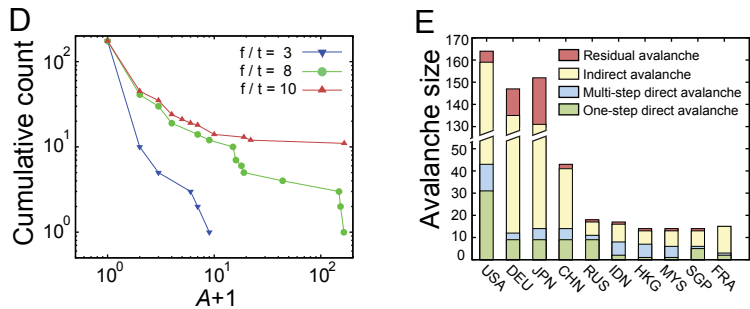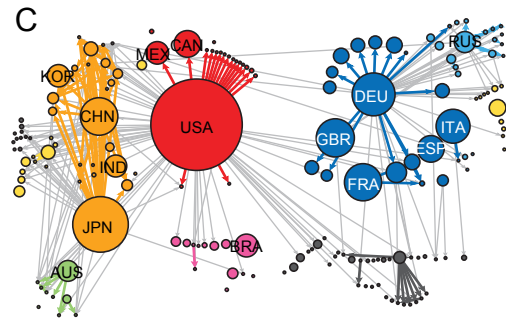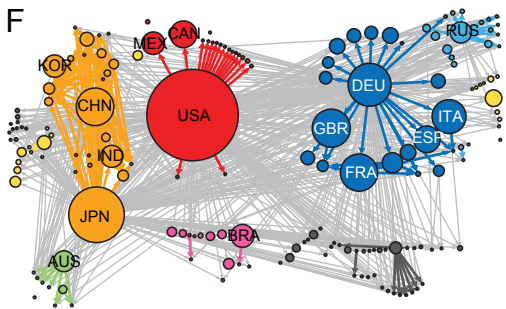

Supplement: Figure S12 — Crisis spreading dynamics properties for the cascade parameter other than 7, (A–C) f/t = 6 and (E–G) f/t = 8. Shown are the avalanche size distribution, the avalanche profiles, and the avalanche network, revealing the approximate power-law P(A), a significant propensity of indirect avalanches, and the intra-continent linkages, respectively, confirming the robustness of the overall conclusion of the model dynamics for a range of model parameters. (PDF) [file pone.0018443.s012.pdf]

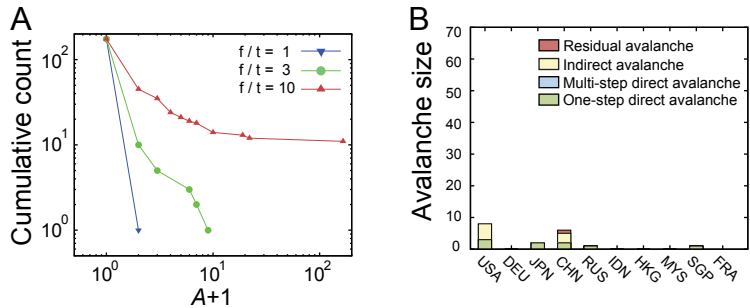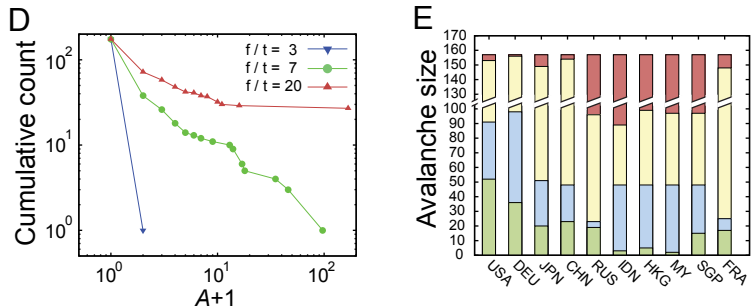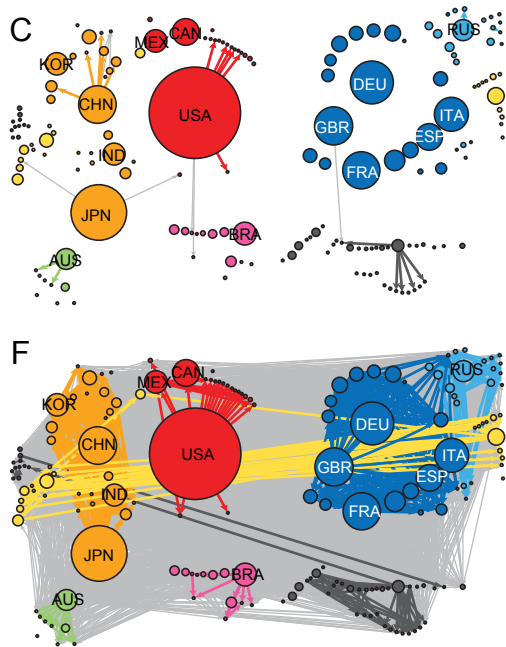

Supplement: Figure S13 — Crisis spreading dynamics properties in the non-power-law regimes. (A–C) f/t = 3 and (E–G) f/t = 20. Shown are the avalanche size distribution, the avalanche profiles, and the avalanche network, revealing the approximate power-law P(A), a significant propensity of indirect avalanches, and the intra-continent linkages, respectively, confirming the robustness of the overall conclusion of the model dynamics for a range of model parameters. (PDF) [file pone.0018443.s013.pdf]

Intra-continental links ratio

GMN  
GSN

0

0.1

0.2

0.3

0.4

0.5

0.6

0.7

0.8

0.9

1

$f/t$

30

25

20

15

10

5

0

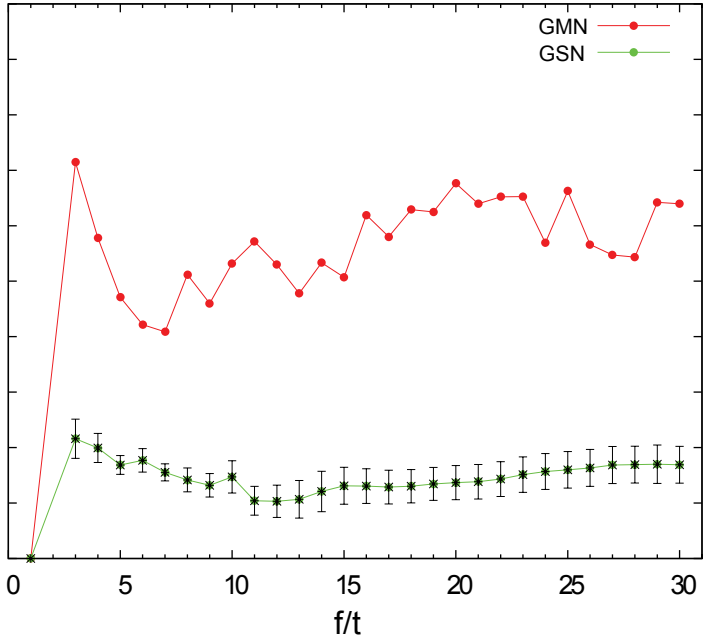

Supplement: Figure S14 — The fraction of intra-continental links as a function of the cascade parameter f/t. Countries with A = 167 were excluded for the calculations. Error-bars in the GSN results are standard deviations obtained from 103 independent randomized structures. (PDF) [file pone.0018443.s014.pdf]
