# Supplementary material for: Impact of the Topology of Global Macroeconomic Network on the Spreading of Economic Crises
Source: PLoS One. 2011 Mar 31;6(3):e18443. doi: 10.1371/journal.pone.0018443 (PMC3069097; doi:10.1371/journal.pone.0018443)
Supplement: Figure S4 — Poster layout of the global avalanche network (intended to be viewed with 300–400% magnification). (PDF) [file pone.0018443.s004.pdf]

The global avalanche network

in *"Impact of the topology of global macroeconomic network on the spreading of economic crises"*

by K.-M. Lee, J.-S. Yang, G. Kim, J. Lee, K.-I. Goh, and I.-M. Kim ⓒ 2011

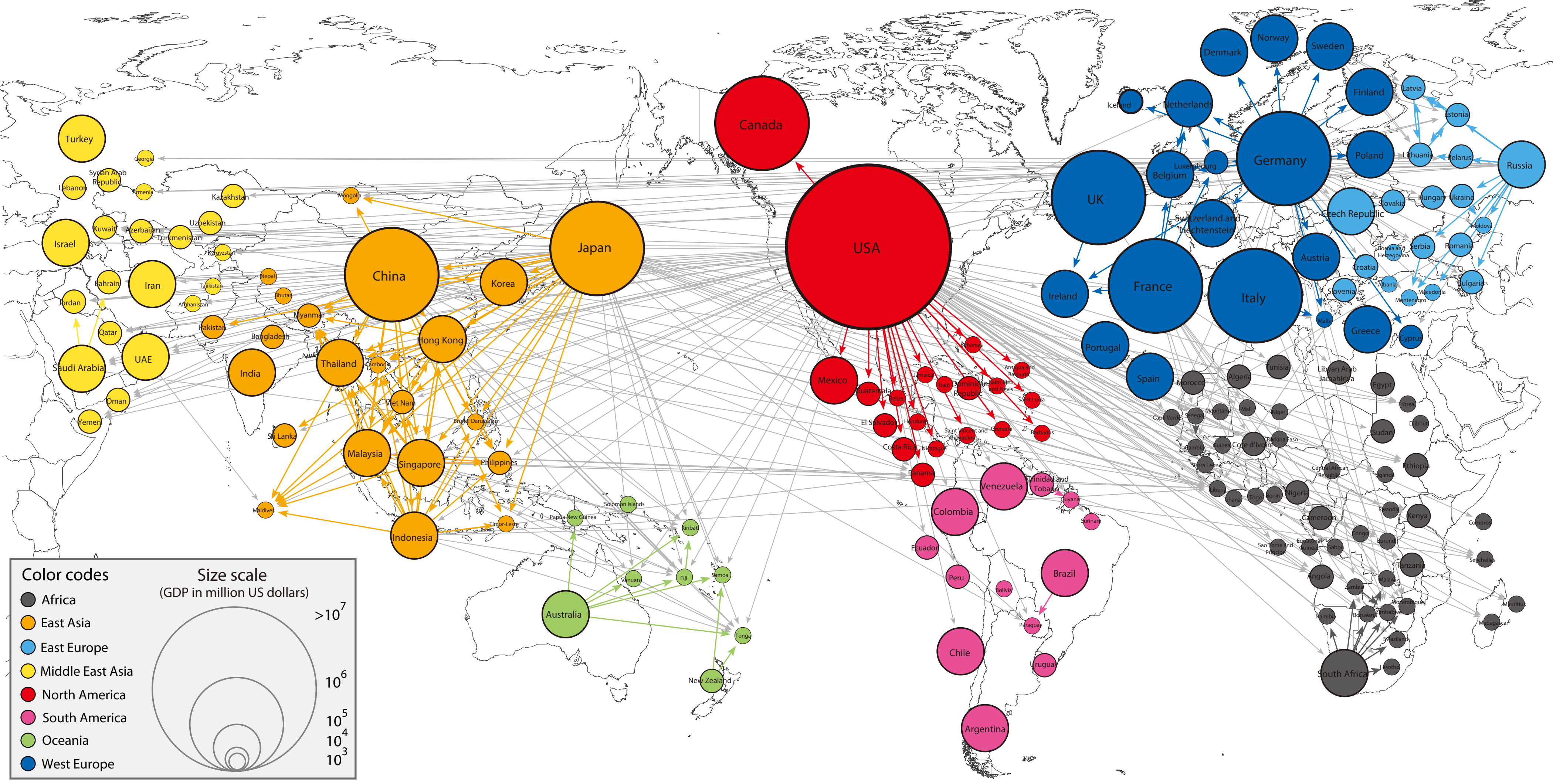

Figure S4. The avalanche network for the world economic network at *f*/*t*=7, overlaid with the world map. The countries are colorcoded according to the continential association and the size of a node follows its GDP (see Legend). An arrow from A to B is placed if the country A makes B collapse in the crisis spreading model. The arrow connecting countries within the same continent is colored following the same color codes, and otherwise it is colored gray.
