# Supplementary material for: Impact of the Topology of Global Macroeconomic Network on the Spreading of Economic Crises
Source: PLoS One. 2011 Mar 31;6(3):e18443. doi: 10.1371/journal.pone.0018443 (PMC3069097; doi:10.1371/journal.pone.0018443)
Supplement: Table S1 — Node and link data for the world economic network. (PDF) [file pone.0018443.s015.pdf]

Supplementary Table S1A. Summary of node data used in the paper.

	Name	Code	Continent	GDP	Total Export	Total Import	Avalanche Size (f/t=7)
1	Afghanistan	AFG	ME	5475.40	186.32	2743.70	0
2	Albania	ALB	EE	6953.60	730.96	2658.41	0
3	Algeria	DZA	AF	85448.00	38974.10	17590.40	0
4	Angola	AGO	AF	24190.00	8615.85	2131.34	0
5	Antigua and Barbuda	ATG	NA	832.80	67.10	491.65	0
6	Argentina	ARG	SA	154318.00	36393.70	25689.50	0
7	Armenia	ARM	ME	4008.60	837.58	1697.02	0
8	Australia	AUS	OC	610070.00	102896.00	114418.00	6
9	Austria	AUT	WE	277480.00	112210.00	121105.00	0
10	Azerbaijan	AZE	ME	11278.80	5002.44	4287.54	0
11	Bahamas	BHS	NA	5755.20	585.40	2916.83	0
12	Bahrain	BHR	ME	11748.80	1534.48	6883.64	0
13	Bangladesh	BGD	EA	57828.00	7701.35	10263.90	1
14	Barbados	BRB	NA	2903.60	330.73	1394.43	0
15	Belarus	BLR	EE	24498.00	15753.80	18633.00	0
16	Belgium	BEL	WE	339370.00	324997.00	316822.00	1
17	Belize	BLZ	NA	1060.80	229.44	448.57	0
18	Benin	BEN	AF	3918.00	387.92	798.09	0
19	Bhutan	BTN	EA	727.20	0.00	0.80	0
20	Bolivia	BOL	SA	9104.80	3098.76	2364.80	0
21	Bosnia and Herzegovina	BIH	EE	9627.20	2619.73	6477.50	0
22	Botswana	BWA	AF	9151.80	4253.16	3608.80	0
23	Brazil	BRA	SA	735122.00	115982.00	73810.00	1
24	Brunei Darussalam	BRN	EA	8272.60	5610.51	1428.80	0
25	Bulgaria	BGR	EE	23860.00	10928.50	17888.80	0
26	Burkina Faso	BFA	AF	4887.60	250.58	967.56	0
27	Burundi	BDI	AF	721.20	126.48	290.44	0
28	Cambodia	KHM	EA	5564.40	2015.02	2545.46	0
29	Cameroon	CMR	AF	14970.00	2130.71	2135.11	0
30	Canada	CAN	NA	1001550.00	351370.00	330741.00	0
31	Cape Verde	CPV	AF	907.00	66.34	688.58	0
32	Central Africa	CAF	AF	1258.20	81.70	71.10	0
33	Chile	CHL	SA	100378.00	42176.10	29312.70	0
34	China	CHN	EA	1982950.00	997233.00	643553.00	34
35	Colombia	COL	SA	103534.00	20416.00	19531.10	0
36	Comoros	COM	AF	346.40	5.09	78.12	0
37	Congo, Democratic Rep.of	COD	AF	6721.40	1469.58	1474.66	0
38	Costa Rica	CRI	NA	19076.00	7231.32	8690.29	0
39	Cote d'Ivoire	CIV	AF	14888.00	6281.80	4854.62	0
40	Croatia	HRV	EE	34014.00	8389.31	19000.30	1
41	Cyprus	CYP	WE	15014.00	969.80	11243.30	0
42	Czech Republic	CZE	EE	108780.00	77503.60	80911.70	1
43	Denmark	DNK	WE	233448.00	71966.60	71964.50	0
44	Djibouti	DJI	AF	673.60	65.26	72.88	0
45	Dominican Republic	DOM	NA	23468.00	64.97	1583.03	0
46	Ecuador	ECU	SA	32954.00	9912.08	9063.37	0
47	Egypt	EGY	AF	88972.00	9063.86	14472.60	0
48	El Salvador	SLV	NA	16176.00	1595.17	5148.91	0
49	Equatorial Guinea	GNQ	AF	5224.80	77.12	113.96	0
50	Eritrea	ERI	AF	1003.40	23.75	398.66	0
51	Estonia	EST	EE	11914.40	7437.26	13093.10	2
52	Ethiopia	ETH	AF	10597.80	761.04	3717.56	0
53	Fiji	FJI	OC	2603.20	565.82	1445.52	1
54	Finland	FIN	WE	179232.00	64090.70	61687.60	3
55	France	FRA	WE	1943790.00	456749.00	530403.00	12
56	Gabon	GAB	AF	7294.80	3311.58	1060.64	0
57	Gambia	GMB	AF	418.60	12.72	302.42	0
58	Georgia	GEO	ME	5337.60	778.92	2649.82	0
59	Germany	DEU	WE	2586450.00	1012450.00	840004.00	45
60	Ghana	GHA	AF	9219.20	2905.07	5041.56	0

61	Greece	GRC	WE	217778.00	15202.20	55769.40	0
62	Grenada	GRD	NA	499.40	31.74	295.59	0
63	Guatemala	GTM	NA	24842.00	3953.74	8349.65	0
64	Guinea	GIN	AF	3433.60	0.00	122.74	0
65	Guyana	GUY	SA	791.80	546.74	696.58	0
66	Haiti	HTI	NA	3696.80	0.00	8.50	0
67	Honduras	HND	NA	9023.80	1531.02	4100.68	0
68	Hong Kong	HKG	EA	171202.00	273438.00	288149.00	13
69	Hungary	HUN	EE	95334.00	61283.60	65878.90	0
70	Iceland	ISL	WE	13234.40	3165.06	4660.34	0
71	India	IND	EA	679594.00	103023.00	123370.00	0
72	Indonesia	IDN	EA	267534.00	92297.70	73482.30	16
73	Iran, Islamic Republic of	IRN	ME	164366.00	47958.40	29448.00	0
74	Ireland	IRL	WE	177164.00	132236.00	72432.20	0
75	Israel	ISR	ME	124500.00	38280.10	41327.10	0
76	Italy	ITA	WE	1620230.00	379777.00	391970.00	3
77	Jamaica	JAM	NA	8896.60	1604.86	4374.66	0
78	Japan	JPN	EA	4341180.00	628353.00	500424.00	45
79	Jordan	JOR	ME	11580.40	3043.60	8370.84	0
80	Kazakhstan	KAZ	ME	47346.00	27956.30	17961.00	0
81	Kenya	KEN	AF	17286.00	2612.95	7184.19	0
82	Kiribati	KIR	OC	58.80	1.80	68.50	0
83	Korea, Republic of	KOR	EA	703488.00	293736.00	251556.00	1
84	Kuwait	KWT	ME	64978.00	24260.40	1362.34	0
85	Kyrgyzstan	KGZ	ME	2207.40	763.56	1356.44	0
86	Latvia	LVA	EE	14081.00	24586.90	9294.58	2
87	Lebanon	LBN	ME	20862.00	942.54	6384.99	0
88	Lesotho	LSO	AF	1192.80	484.52	844.12	0
89	Liberia	LBR	AF	510.00	215.20	1033.79	0
90	Libyan Arab Jamahiriya	LBY	AF	33150.00	21036.10	5082.32	0
91	Lithuania	LTU	EE	22138.00	11404.70	18230.40	2
92	Luxembourg	LUX	WE	33158.00	12604.60	17211.00	0
93	Macedonia	MKD	EE	5191.80	2313.79	3405.92	0
94	Madagascar	MDG	AF	4955.60	956.74	1513.81	0
95	Malawi	MWI	AF	2747.00	555.29	1066.86	0
96	Malaysia	MYS	EA	125824.00	164130.00	120858.00	13
97	Maldives	MDV	EA	753.40	133.38	750.18	0
98	Mali	MLI	AF	4868.20	1150.68	1535.40	0
99	Malta	MLT	WE	5425.40	2122.57	3962.26	0
100	Mauritania	MRT	AF	1699.80	490.08	691.36	0
101	Mauritius	MUS	AF	5634.20	1898.54	2778.65	0
102	Mexico	MEX	NA	715714.00	221165.00	213238.00	0
103	Moldova, Republic of	MDA	EE	2517.00	1013.84	2496.02	0
104	Mongolia	MNG	EA	1999.80	1188.44	1265.72	0
105	Montenegro	MNE	EE	2084.00	0.00	840.35	0
106	Morocco	MAR	AF	54210.00	10892.80	20039.50	0
107	Mozambique	MOZ	AF	5584.60	1994.54	2137.93	0
108	Myanmar	MMR	EA	10667.60	1736.62	0.00	0
109	Namibia	NAM	AF	5288.60	2636.08	2582.62	0
110	Nepal	NPL	EA	7373.40	623.15	1496.09	0
111	Netherlands	NLD	WE	578870.00	347851.00	380878.00	4
112	New Zealand	NZL	OC	90452.00	18399.20	23946.70	2
113	Nicaragua	NIC	NA	4547.80	891.70	2653.51	0
114	Niger	NER	AF	2907.00	323.04	673.24	0
115	Nigeria	NGA	AF	94246.00	47278.40	14006.90	0
116	Norway	NOR	WE	263418.00	109885.00	59127.60	0
117	Oman	OMN	ME	26706.00	17708.80	8943.23	0
118	Pakistan	PAK	EA	98176.00	13168.00	20923.30	0
119	Panama	PAN	NA	14396.00	2680.77	13833.70	0
120	Papua New Guinea	PNG	OC	4192.60	1531.17	1465.69	0
121	Paraguay	PRY	SA	6980.40	1984.62	3967.56	0
122	Peru	PER	SA	72072.00	16917.40	12342.20	0
123	Philippines	PHL	EA	91930.00	55593.50	47625.20	0

124	Poland	POL	WE	262746.00	88029.70	110816.00	0
125	Portugal	PRT	WE	168912.00	35556.40	58222.20	2
126	Qatar	QAT	ME	33960.00	24417.70	7660.02	0
127	Romania	ROU	EE	80474.00	26582.30	41159.80	1
128	Russia	RUS	EE	624336.00	276423.00	139261.00	17
129	Rwanda	RWA	AF	2130.40	118.78	396.60	0
130	Samoa	WSM	OC	312.60	72.90	171.18	0
131	Sao Tome and Principe	STP	AF	107.20	4.40	46.12	0
132	Saudi Arabia	SAU	ME	263846.00	153716.00	54188.40	3
133	Senegal	SEN	AF	7616.00	1094.46	3143.42	1
134	Serbia	SRB	EE	23742.00	6186.25	12849.70	1
135	Seychelles	SYC	AF	720.40	327.50	620.96	0
136	Sierra Leone	SLE	AF	1127.60	0.00	3.89	0
137	Singapore	SGP	EA	109388.00	211952.00	185947.00	13
138	Slovakia	SVK	EE	40774.00	35179.70	36571.80	0
139	Slovenia	SVN	EE	32050.00	17575.20	19625.10	0
140	Solomon Islands	SLB	OC	269.00	0.35	4.10	0
141	South Africa	ZAF	AF	198726.00	53453.30	61859.00	8
142	Spain	ESP	WE	996630.00	186039.00	301827.00	0
143	Sri Lanka	LKA	EA	21068.00	4654.90	6990.47	0
144	St. Kitts and Nevis	KNA	NA	409.00	37.00	220.22	0
145	St. Lucia	LCA	NA	810.40	69.90	473.39	0
146	St. Vincent and the Grenadines	VCT	NA	421.40	37.30	219.13	0
147	Sudan	SDN	AF	23648.00	3954.90	6109.84	0
148	Suriname	SUR	SA	1556.60	46.54	903.02	0
149	Swaziland	SWZ	AF	2157.80	1623.28	1423.08	0
150	Sweden	SWE	WE	335926.00	134887.00	113860.00	3
151	Switzerland and Liechtenstein	CHE	WE	345838.00	150709.00	153676.00	0
152	Syrian Arab Republic	SYR	ME	26812.00	6048.30	5416.26	0
153	Tajikistan	TJK	ME	1992.40	0.00	91.70	0
154	Tanzania, United Republic of	TZA	AF	12734.00	1571.48	3492.26	0
155	Thailand	THA	EA	162796.00	114328.00	103303.00	2
156	Timor-Leste	TLS	EA	344.20	73.80	107.25	0
157	Togo	TGO	AF	1882.60	527.83	863.13	0
158	Tonga	TON	OC	184.40	11.48	114.38	0
159	Trinidad and Tobago	TTO	SA	13235.80	9159.52	5284.45	2
160	Tunisia	TUN	AF	26854.00	10414.60	13047.30	0
161	Turkey	TUR	ME	387906.00	67779.00	111132.00	0
162	Turkmenistan	TKM	ME	14668.00	3257.82	280.28	0
163	Uganda	UGA	AF	7419.60	778.52	2049.74	0
164	Ukraine	UKR	EE	70002.00	31858.80	38503.20	2
165	United Arab Emirates	ARE	ME	112790.00	74456.70	83737.70	1
166	United Kingdom	GBR	WE	2046070.00	387173.00	512130.00	5
167	United States	USA	NA	11749000.00	961453.00	1666450.00	96
168	Uruguay	URY	SA	14524.00	2842.22	4025.29	0
169	Uzbekistan	UZB	ME	12625.40	1712.74	407.06	0
170	Vanuatu	VUT	OC	324.60	19.25	174.65	0
171	Venezuela, Bolivarian Rep. of	VEN	SA	123502.00	48798.90	20327.00	0
172	Viet Nam	VNM	EA	46876.00	25973.40	31155.20	0
173	Yemen	YEM	ME	14444.00	5167.65	4584.61	0
174	Zambia	ZMB	AF	6340.40	2659.29	2882.32	1
175	Zimbabwe	ZWE	AF	2399.60	3083.56	2404.98	0

Supplementary Table S1B. Summary of link data used in the paper.

Export Country	Import Country	Trade Volume
AFG	BEL	1.54
AFG	FIN	4.26
AFG	FRA	0.12
AFG	DEU	4.42
AFG	IND	18.28
AFG	IRN	2.00
AFG	PAN	141.40
AFG	RWA	6.20
AFG	GBR	2.28
AFG	USA	1.04
AFG	URY	1.58
AFG	VUT	3.20
ALB	AUT	2.74
ALB	BIH	2.02
ALB	BGR	3.22
ALB	CHN	8.42
ALB	HRV	0.82
ALB	FRA	4.44
ALB	GEO	0.74
ALB	DEU	21.46
ALB	GRC	72.94
ALB	IND	1.68
ALB	ITA	512.26
ALB	MDG	11.58
ALB	MAR	2.35
ALB	NZL	0.90
ALB	RUS	1.16
ALB	RWA	1.30
ALB	SYC	56.55
ALB	SLB	1.08
ALB	LKA	0.94
ALB	CHE	6.04
ALB	SYR	0.76
ALB	TKM	12.14
ALB	USA	0.94
ALB	URY	4.48
DZA	BEL	1958.82
DZA	BRA	2021.76
DZA	CAN	3332.12
DZA	CHN	140.46
DZA	EGY	502.72
DZA	FRA	4289.70
DZA	DEU	518.44
DZA	GRC	116.38
DZA	IND	186.58
DZA	IDN	156.22
DZA	ITA	6128.12
DZA	JPN	62.26
DZA	KOR	273.70
DZA	MUS	5.66
DZA	MDA	63.22
DZA	MOZ	386.08
DZA	NZL	2325.76
DZA	QAT	1004.86
DZA	LKA	4733.28
DZA	SYR	103.22
DZA	TLS	81.40
DZA	TUR	167.06
DZA	TKM	1593.94
DZA	USA	717.60

DZA	URY	8104.72
AGO	CHL	971.23
AGO	CHN	7465.40
AGO	PHL	171.50
AGO	SAU	7.72
ATG	BRB	4.20
ATG	DOM	5.30
ATG	FIN	0.30
ATG	FRA	2.90
ATG	DEU	0.65
ATG	GRD	0.40
ATG	GUY	0.45
ATG	ITA	0.95
ATG	JAM	3.00
ATG	MUS	0.30
ATG	NZL	2.00
ATG	NER	1.95
ATG	SEN	0.80
ATG	SRB	0.90
ATG	SGP	3.85
ATG	LCA	8.15
ATG	VCT	1.15
ATG	TUN	0.90
ATG	USA	12.15
ATG	URY	16.25
ATG	VNM	0.55
ARG	DZA	482.42
ARG	BGD	223.64
ARG	BOL	402.34
ARG	BRA	7006.10
ARG	CPV	1.96
ARG	CHL	4433.95
ARG	CHN	3381.12
ARG	COL	457.64
ARG	CRI	59.54
ARG	ECU	328.54
ARG	EGY	500.14
ARG	SLV	82.58
ARG	FRA	434.08
ARG	GAB	6.48
ARG	DEU	929.26
ARG	GTM	118.26
ARG	HND	50.52
ARG	IND	724.88
ARG	ITA	1068.96
ARG	JPN	419.56
ARG	KOR	447.76
ARG	LSO	0.36
ARG	LBY	76.07
ARG	MWI	24.92
ARG	MYS	6.04
ARG	MDV	403.58
ARG	MEX	34.84
ARG	MDA	1337.22
ARG	MOZ	293.98
ARG	MMR	20.78
ARG	NLD	13.10
ARG	NZL	1380.96
ARG	NER	41.72
ARG	NGA	7.26
ARG	PNG	48.98
ARG	PER	676.66
ARG	PHL	747.92

ARG	POL	297.14
ARG	RWA	582.38
ARG	SRB	54.16
ARG	ESP	651.36
ARG	LKA	1640.46
ARG	TJK	122.72
ARG	TUN	26.32
ARG	TUR	148.52
ARG	USA	469.92
ARG	URY	3975.04
ARG	UZB	896.76
ARG	VNM	613.38
ARG	YEM	187.92
ARG	MKD	53.46
ARM	AUT	2.78
ARM	BEL	113.02
ARM	BLZ	6.10
ARM	BGR	11.02
ARM	CAN	7.92
ARM	CHN	8.78
ARM	FIN	2.90
ARM	FRA	3.64
ARM	GEO	37.64
ARM	DEU	117.68
ARM	IRN	28.66
ARM	ISR	97.18
ARM	ITA	26.24
ARM	KAZ	3.22
ARM	NZL	92.20
ARM	PRT	3.12
ARM	RWA	119.50
ARM	LKA	8.86
ARM	SYR	46.78
ARM	ARE	19.86
ARM	GBR	9.52
ARM	USA	10.90
ARM	URY	60.06
AUS	BHR	298.77
AUS	BGD	200.92
AUS	BEL	760.84
AUS	BWA	7.42
AUS	BRN	36.37
AUS	KHM	7.38
AUS	CAN	1419.06
AUS	CHN	16042.50
AUS	EGY	289.12
AUS	FJI	407.84
AUS	FIN	686.72
AUS	FRA	903.86
AUS	DEU	996.12
AUS	GHA	83.18
AUS	HKG	2117.98
AUS	IND	5163.00
AUS	IDN	2701.26
AUS	ITA	1175.94
AUS	JPN	23645.20
AUS	KIR	26.30
AUS	KOR	9550.64
AUS	MDV	2119.96
AUS	MLI	18.94
AUS	MLT	13.28
AUS	MEX	91.64
AUS	MNE	17.24

AUS	NLD	14.03
AUS	NZL	1620.90
AUS	NIC	6641.78
AUS	NOR	142.37
AUS	PAK	181.24
AUS	PAN	353.80
AUS	PRY	949.26
AUS	POL	730.80
AUS	ROU	108.18
AUS	STP	39.20
AUS	SEN	1590.94
AUS	SLE	7.26
AUS	SVK	2908.00
AUS	ESP	1452.92
AUS	KNA	138.28
AUS	SDN	1.56
AUS	SUR	172.42
AUS	THA	47.76
AUS	TLS	2883.44
AUS	TGO	17.10
AUS	TTO	17.94
AUS	UKR	20.90
AUS	GBR	1324.84
AUS	USA	4849.94
AUS	URY	7228.26
AUS	VEN	64.20
AUS	YEM	524.26
AUS	MKD	60.48
AUS	ZMB	22.18
AUT	ALB	38.62
AUT	DZA	165.90
AUT	ARM	36.74
AUT	AUS	696.68
AUT	BHR	75.67
AUT	BLR	99.36
AUT	BEL	1887.42
AUT	BIH	273.50
AUT	BGR	553.38
AUT	CAN	986.08
AUT	CHN	1528.28
AUT	HRV	1562.52
AUT	CZE	3842.10
AUT	DNK	880.90
AUT	EST	108.46
AUT	FIN	581.78
AUT	FRA	4857.74
AUT	GAB	6.02
AUT	GEO	28.10
AUT	DEU	37936.80
AUT	GRC	655.70
AUT	HUN	4564.74
AUT	IRN	469.18
AUT	ITA	10453.80
AUT	JPN	1287.62
AUT	KAZ	160.14
AUT	LVA	145.04
AUT	LTU	178.84
AUT	LUX	157.96
AUT	MDG	75.22
AUT	MRT	29.24
AUT	MNG	22.24
AUT	NZL	2191.08
AUT	OMN	489.92

AUT	PRT	2540.04
AUT	QAT	455.10
AUT	RUS	1920.74
AUT	RWA	2283.16
AUT	SYC	344.63
AUT	SVN	1999.86
AUT	SLB	2446.28
AUT	LKA	3238.90
AUT	CHE	1294.26
AUT	SYR	5814.66
AUT	TKM	989.58
AUT	ARE	493.08
AUT	USA	4700.16
AUT	URY	6663.24
AZE	BGR	35.22
AZE	CHL	22.92
AZE	CHN	33.34
AZE	HRV	78.14
AZE	CZE	620.62
AZE	FRA	257.94
AZE	GEO	237.16
AZE	GRC	96.88
AZE	HKG	26.86
AZE	IND	30.84
AZE	IDN	103.94
AZE	IRN	219.94
AZE	ISR	342.30
AZE	ITA	1612.56
AZE	KAZ	54.00
AZE	KOR	39.70
AZE	MRT	42.44
AZE	RUS	73.70
AZE	RWA	302.88
AZE	SVK	33.84
AZE	LKA	46.10
AZE	TZA	68.46
AZE	TKM	402.00
AZE	UGA	89.18
AZE	ARE	27.38
AZE	URY	104.10
BHS	ATG	8.00
BHS	ARG	2.90
BHS	AUS	2.80
BHS	BRA	1.65
BHS	CAN	27.45
BHS	CHN	5.30
BHS	FRA	36.50
BHS	DEU	22.90
BHS	GTM	1.55
BHS	HKG	1.15
BHS	JAM	0.50
BHS	JPN	1.00
BHS	MDA	0.65
BHS	NZL	25.05
BHS	PNG	1.25
BHS	ESP	4.95
BHS	LKA	2.95
BHS	CHE	0.50
BHS	USA	15.85
BHS	URY	421.55
BHS	UZB	0.95
BHR	BEN	7.84
BHR	BFA	7.72

BHR	KEN	121.20
BHR	MWI	201.30
BHR	MLI	10.38
BHR	MEX	89.18
BHR	PAK	98.06
BHR	ROU	72.22
BHR	WSM	2.68
BHR	SEN	549.68
BHR	SUR	58.36
BHR	THA	282.02
BHR	UKR	33.84
BGD	BEL	309.94
BGD	CAN	284.34
BGD	CHN	189.86
BGD	DNK	81.12
BGD	FRA	547.64
BGD	DEU	1253.84
BGD	HKG	106.60
BGD	IND	121.40
BGD	IRN	41.80
BGD	IRL	59.34
BGD	ITA	334.64
BGD	JPN	116.18
BGD	KOR	36.36
BGD	NLD	7.27
BGD	NZL	296.28
BGD	PAN	49.22
BGD	SEN	28.40
BGD	SVK	61.80
BGD	LKA	293.36
BGD	CHE	132.30
BGD	TJK	32.26
BGD	TLS	28.44
BGD	TKM	67.78
BGD	USA	841.88
BGD	URY	2379.30
BRB	ATG	8.42
BRB	BHS	3.15
BRB	BLZ	1.68
BRB	CAN	6.40
BRB	CHN	1.70
BRB	DOM	4.88
BRB	FRA	3.50
BRB	DEU	2.18
BRB	GRD	9.04
BRB	GTM	86.36
BRB	GUY	7.02
BRB	JAM	25.40
BRB	NZL	1.38
BRB	LCA	6.30
BRB	VCT	18.06
BRB	SDN	11.64
BRB	SWZ	4.24
BRB	SYR	1.60
BRB	TUN	46.28
BRB	USA	30.14
BRB	URY	51.36
BLR	BEL	87.14
BLR	BRA	201.16
BLR	CHN	355.66
BLR	CZE	61.50
BLR	DNK	78.74
BLR	EST	156.62

BLR	FRA	142.90
BLR	DEU	623.20
BLR	HUN	122.80
BLR	IND	105.68
BLR	ITA	158.98
BLR	KAZ	225.42
BLR	KGZ	11.94
BLR	LVA	489.02
BLR	LTU	394.06
BLR	MNG	94.42
BLR	MNE	5.24
BLR	NZL	2303.66
BLR	PRT	855.48
BLR	RUS	74.10
BLR	RWA	6562.54
BLR	SVN	52.14
BLR	CHE	181.54
BLR	ARE	905.72
BLR	USA	1242.02
BLR	URY	262.16
BEL	ALB	16.36
BEL	DZA	410.02
BEL	ARG	297.46
BEL	ARM	126.22
BEL	AUS	1040.32
BEL	AUT	3491.42
BEL	BHR	52.33
BEL	BRB	6.14
BEL	BLR	131.92
BEL	BEN	30.52
BEL	BOL	10.26
BEL	BWA	23.68
BEL	BRA	788.64
BEL	BRN	6.20
BEL	BGR	278.04
BEL	BFA	21.56
BEL	BDI	33.56
BEL	KHM	3.08
BEL	CMR	102.32
BEL	CAN	2288.74
BEL	CPV	16.38
BEL	CAF	5.60
BEL	CHN	3399.48
BEL	COM	1.42
BEL	COD	30.50
BEL	CIV	88.26
BEL	HRV	221.36
BEL	CYP	133.00
BEL	CZE	2343.10
BEL	DNK	2751.96
BEL	DOM	0.68
BEL	ECU	78.88
BEL	ERI	11.45
BEL	EST	190.54
BEL	ETH	70.56
BEL	FIN	1340.38
BEL	FRA	57820.40
BEL	GAB	119.64
BEL	GMB	8.36
BEL	GEO	26.74
BEL	DEU	66632.70
BEL	GHA	282.62
BEL	GRC	2207.78

BEL	GUY	5.34
BEL	HKG	2265.02
BEL	HUN	1411.94
BEL	ISL	81.12
BEL	IND	5849.90
BEL	IRN	560.73
BEL	IRL	2735.08
BEL	ISR	4051.92
BEL	ITA	17805.00
BEL	JAM	26.98
BEL	JPN	3156.66
BEL	KAZ	145.84
BEL	KEN	93.50
BEL	KGZ	6.98
BEL	LVA	165.64
BEL	LBN	141.28
BEL	LBY	96.07
BEL	LTU	392.14
BEL	LUX	6298.78
BEL	MWI	35.28
BEL	MYS	10.90
BEL	MLT	47.28
BEL	MRT	72.04
BEL	MUS	33.36
BEL	MEX	48.22
BEL	MNG	28.96
BEL	MNE	4.72
BEL	MOZ	410.50
BEL	MMR	17.56
BEL	NPL	11.02
BEL	NLD	13.63
BEL	NZL	40113.00
BEL	NIC	205.66
BEL	NGA	16.78
BEL	NOR	741.30
BEL	OMN	1307.58
BEL	PAK	98.96
BEL	PAN	253.82
BEL	PRY	5.50
BEL	PRT	4023.84
BEL	QAT	2154.50
BEL	ROU	104.32
BEL	RUS	717.14
BEL	RWA	2778.08
BEL	WSM	34.00
BEL	SAU	3.84
BEL	SEN	815.60
BEL	SRB	93.96
BEL	SLE	6.16
BEL	SVN	523.14
BEL	SLB	422.08
BEL	ESP	735.90
BEL	LKA	12805.90
BEL	KNA	196.80
BEL	SUR	50.92
BEL	SWZ	5.74
BEL	CHE	4871.80
BEL	SYR	4501.00
BEL	TJK	80.60
BEL	THA	61.06
BEL	TGO	0.25
BEL	TON	25.38
BEL	TUR	369.08

BEL	TKM	3315.38
BEL	UKR	34.76
BEL	ARE	338.82
BEL	GBR	978.96
BEL	USA	27856.50
BEL	URY	21031.50
BEL	VNM	184.02
BEL	YEM	159.34
BEL	ZWE	9.95
BLZ	BRB	1.36
BLZ	CAN	0.36
BLZ	CRI	10.60
BLZ	DOM	1.48
BLZ	SLV	1.22
BLZ	FIN	2.70
BLZ	FRA	0.70
BLZ	DEU	0.58
BLZ	GTM	1.32
BLZ	GUY	0.94
BLZ	HND	0.60
BLZ	IRL	3.08
BLZ	ITA	0.26
BLZ	JAM	9.38
BLZ	JPN	2.90
BLZ	MDA	6.18
BLZ	NZL	11.46
BLZ	PNG	9.40
BLZ	QAT	3.54
BLZ	LKA	2.16
BLZ	TUN	9.24
BLZ	USA	44.12
BLZ	URY	105.86
BEN	BGD	4.90
BEN	BEL	2.54
BEN	BRA	2.56
BEN	BFA	22.38
BEN	CHN	55.38
BEN	DNK	2.46
BEN	FRA	6.60
BEN	DEU	2.44
BEN	GHA	10.14
BEN	IND	33.64
BEN	IDN	18.88
BEN	ITA	7.10
BEN	MDV	2.32
BEN	MLT	113.36
BEN	MOZ	4.36
BEN	NGA	28.40
BEN	NOR	24.06
BEN	PAN	5.22
BEN	QAT	2.78
BEN	ESP	2.96
BEN	LKA	4.02
BEN	SYR	3.04
BEN	TLS	12.18
BEN	TON	11.60
BEN	YEM	4.60
BOL	ARG	252.92
BOL	BEL	51.98
BOL	BRA	1116.78
BOL	CAN	38.68
BOL	CHL	51.78
BOL	CHN	29.24

BOL	COL	165.16
BOL	ECU	9.76
BOL	FRA	7.98
BOL	DEU	13.16
BOL	ITA	20.24
BOL	JPN	201.16
BOL	KOR	76.20
BOL	MDV	16.98
BOL	MDA	24.00
BOL	NZL	26.92
BOL	PNG	23.84
BOL	PER	20.16
BOL	PHL	165.42
BOL	LKA	14.36
BOL	SYR	139.16
BOL	USA	66.32
BOL	URY	366.24
BOL	VNM	200.32
BIH	AUT	136.88
BIH	BEL	16.14
BIH	BGR	13.66
BIH	HRV	486.02
BIH	CZE	20.64
BIH	FRA	38.68
BIH	DEU	324.56
BIH	HUN	83.62
BIH	ITA	346.28
BIH	LTU	20.22
BIH	MDG	21.32
BIH	MAR	54.45
BIH	NZL	21.34
BIH	PRT	31.68
BIH	RUS	23.22
BIH	SYC	511.30
BIH	SLB	272.30
BIH	LKA	14.48
BIH	CHE	12.10
BIH	SYR	73.10
BIH	TKM	12.32
BIH	USA	18.74
BIH	URY	66.68
BWA	AGO	1.66
BWA	BEL	19.90
BWA	CAN	26.52
BWA	CHN	21.16
BWA	COD	1.72
BWA	FIN	8.36
BWA	FRA	5.02
BWA	DEU	26.46
BWA	GRC	7.20
BWA	GRD	9.74
BWA	ISR	26.64
BWA	ITA	2.14
BWA	JPN	1.20
BWA	LSO	0.48
BWA	NPL	8.40
BWA	NZL	2.88
BWA	OMN	315.90
BWA	QAT	10.40
BWA	SVK	1.94
BWA	ESP	361.26
BWA	SWE	2.28
BWA	SYR	4.54

BWA	USA	3106.14
BWA	URY	60.76
BWA	ZMB	13.92
BWA	ZWE	206.54
BRA	ALB	31.08
BRA	DZA	353.98
BRA	ATG	4.63
BRA	ARG	9883.80
BRA	AZE	46.44
BRA	BHS	4.50
BRA	BHR	138.73
BRA	BGD	179.00
BRA	BRB	25.60
BRA	BLR	131.30
BRA	BEL	2548.02
BRA	BLZ	3.98
BRA	BEN	7.06
BRA	BOL	526.44
BRA	BIH	57.04
BRA	BGR	277.20
BRA	CMR	47.54
BRA	CAN	2394.72
BRA	CPV	30.04
BRA	CAF	0.43
BRA	CHL	3825.58
BRA	CHN	11151.30
BRA	COL	1556.66
BRA	COM	1.82
BRA	CRI	351.34
BRA	CIV	65.78
BRA	HRV	147.62
BRA	CYP	120.36
BRA	DOM	1.74
BRA	ECU	641.24
BRA	EGY	596.82
BRA	SLV	226.92
BRA	ERI	10.33
BRA	ETH	49.00
BRA	FIN	533.26
BRA	FRA	2568.76
BRA	GAB	17.70
BRA	GMB	9.90
BRA	GEO	40.46
BRA	DEU	7549.12
BRA	GHA	130.84
BRA	GRD	5.10
BRA	GTM	274.40
BRA	GUY	11.94
BRA	HND	116.72
BRA	HKG	1012.66
BRA	IDN	485.98
BRA	IRN	1275.28
BRA	ITA	3767.60
BRA	JAM	168.74
BRA	JPN	3357.64
BRA	KAZ	139.02
BRA	KOR	1711.72
BRA	KGZ	4.26
BRA	LBN	65.50
BRA	LBY	58.63
BRA	MDG	43.30
BRA	MLT	33.56
BRA	MUS	27.48

BRA	MDA	4791.08
BRA	MOZ	436.50
BRA	MMR	18.48
BRA	NPL	12.06
BRA	NZL	6008.38
BRA	NER	76.24
BRA	NGA	21.06
BRA	NOR	274.03
BRA	OMN	545.66
BRA	PAK	61.24
BRA	PNG	128.25
BRA	PER	1085.34
BRA	PHL	1151.78
BRA	QAT	1288.02
BRA	ROU	82.06
BRA	RUS	448.44
BRA	RWA	2652.06
BRA	SAU	0.14
BRA	SEN	1187.76
BRA	SRB	124.10
BRA	ESP	1272.58
BRA	LKA	2754.24
BRA	LCA	1.00
BRA	VCT	4.34
BRA	SDN	3.00
BRA	SUR	53.96
BRA	SWZ	31.20
BRA	TJK	103.36
BRA	TON	13.40
BRA	TTO	0.06
BRA	TUN	669.90
BRA	TUR	162.12
BRA	ARE	330.78
BRA	GBR	638.08
BRA	USA	2550.34
BRA	URY	24628.40
BRA	UZB	866.78
BRA	VNM	2518.20
BRA	MKD	139.96
BRN	AUS	531.20
BRN	CAN	1.93
BRN	CHN	225.90
BRN	FRA	0.60
BRN	DEU	3.60
BRN	HKG	1.53
BRN	IND	153.53
BRN	IDN	1016.26
BRN	JPN	1824.87
BRN	KOR	677.23
BRN	MDV	107.43
BRN	NLD	0.27
BRN	NZL	1.30
BRN	NIC	159.22
BRN	NOR	0.93
BRN	PAK	0.27
BRN	POL	7.93
BRN	RWA	1.27
BRN	SVK	174.30
BRN	LKA	3.37
BRN	TLS	335.83
BRN	GBR	0.97
BRN	USA	8.37
BRN	URY	372.13

BRN	YEM	0.27
BGR	ALB	63.60
BGR	ARM	32.84
BGR	AUT	245.88
BGR	BEL	777.16
BGR	BIH	55.06
BGR	HRV	135.42
BGR	CYP	44.16
BGR	FRA	548.62
BGR	GEO	99.18
BGR	DEU	1270.06
BGR	GRC	1182.46
BGR	HUN	114.70
BGR	ITA	1440.64
BGR	MDG	263.18
BGR	MNG	34.84
BGR	NZL	163.70
BGR	PRT	163.76
BGR	RUS	521.94
BGR	RWA	212.14
BGR	SYC	724.70
BGR	SVK	181.72
BGR	SLB	103.92
BGR	LKA	370.98
BGR	TKM	1356.80
BGR	ARE	111.34
BGR	USA	310.04
BGR	URY	399.70
BFA	BEL	7.22
BFA	BEN	3.10
BFA	CIV	13.76
BFA	DNK	1.00
BFA	FRA	44.08
BFA	DEU	0.78
BFA	GHA	67.72
BFA	HKG	0.72
BFA	ITA	4.08
BFA	JPN	2.36
BFA	MLT	16.84
BFA	NZL	0.96
BFA	NGA	13.24
BFA	NOR	1.16
BFA	QAT	0.96
BFA	RWA	0.56
BFA	SVK	6.08
BFA	ESP	0.46
BFA	LKA	1.36
BFA	SYR	27.70
BFA	TON	32.14
BFA	USA	2.34
BFA	URY	0.78
BFA	VNM	1.18
BDI	ALB	0.64
BDI	BEL	6.66
BDI	COD	4.04
BDI	ETH	1.34
BDI	FRA	1.86
BDI	DEU	1.88
BDI	JPN	4.40
BDI	KEN	10.82
BDI	MMR	1.04
BDI	NLD	1.72
BDI	NZL	1.70

BDI	PAN	5.98
BDI	RWA	0.78
BDI	WSM	5.10
BDI	SVK	0.50
BDI	ESP	1.12
BDI	SUR	0.64
BDI	SWE	0.70
BDI	SYR	32.92
BDI	THA	0.78
BDI	TLS	1.04
BDI	UKR	1.80
BDI	GBR	32.46
BDI	USA	6.02
BDI	URY	0.54
KHM	BEL	4.96
KHM	CAN	36.00
KHM	CHN	13.68
KHM	FRA	42.60
KHM	DEU	135.40
KHM	GUY	1.86
KHM	HKG	375.28
KHM	IRL	11.98
KHM	ITA	4.80
KHM	JPN	17.98
KHM	KOR	6.00
KHM	LUX	2.16
KHM	MDV	13.76
KHM	NZL	25.60
KHM	OMN	3.80
KHM	POL	2.32
KHM	SVK	22.56
KHM	LKA	13.64
KHM	CHE	8.08
KHM	SYR	5.36
KHM	TLS	13.56
KHM	USA	132.28
KHM	URY	997.28
KHM	YEM	124.08
CMR	BEL	85.58
CMR	CAF	14.05
CMR	CHN	85.92
CMR	COD	37.40
CMR	GNQ	19.12
CMR	FRA	299.06
CMR	GAB	35.04
CMR	DEU	25.66
CMR	IND	13.64
CMR	IRL	15.04
CMR	ITA	416.86
CMR	NZL	224.34
CMR	NGA	6.20
CMR	NOR	18.76
CMR	PAN	13.16
CMR	QAT	24.76
CMR	SAU	0.16
CMR	ESP	31.00
CMR	LKA	499.72
CMR	TON	19.94
CMR	TKM	16.86
CMR	USA	73.84
CMR	URY	154.60
CAN	DZA	287.08
CAN	ATG	9.30

CAN	AUS	1456.92
CAN	BHS	22.80
CAN	BRB	53.62
CAN	BEL	1966.02
CAN	BLZ	6.60
CAN	BOL	16.28
CAN	BWA	11.40
CAN	BRA	1107.60
CAN	BRN	6.20
CAN	BDI	1.54
CAN	KHM	2.46
CAN	CHL	549.68
CAN	CHN	7575.98
CAN	COL	447.34
CAN	CRI	87.80
CAN	DOM	4.30
CAN	ECU	143.66
CAN	SLV	52.86
CAN	FJI	5.82
CAN	FRA	2190.62
CAN	DEU	2782.38
CAN	GHA	108.36
CAN	GRD	6.78
CAN	GTM	121.92
CAN	GUY	17.54
CAN	HKG	1284.36
CAN	ISL	86.84
CAN	IND	1045.04
CAN	IDN	658.72
CAN	IRN	206.68
CAN	IRL	435.02
CAN	ISR	320.74
CAN	ITA	1645.88
CAN	JAM	114.48
CAN	JPN	8894.40
CAN	KAZ	120.00
CAN	KOR	2599.62
CAN	KGZ	18.22
CAN	LSO	0.46
CAN	LBY	61.97
CAN	LUX	87.00
CAN	MDV	558.12
CAN	MLI	7.00
CAN	MDA	6190.00
CAN	MNE	9.72
CAN	MOZ	187.44
CAN	NZL	2185.84
CAN	NIC	389.00
CAN	NER	22.00
CAN	OMN	1704.00
CAN	PAN	288.08
CAN	PNG	29.75
CAN	PRY	2.34
CAN	PHL	229.34
CAN	RWA	573.58
CAN	STP	0.24
CAN	SEN	605.60
CAN	SVK	586.92
CAN	LKA	945.72
CAN	LCA	8.94
CAN	VCT	11.72
CAN	SDN	7.02
CAN	SUR	77.54

CAN	SWZ	9.16
CAN	SYR	806.16
CAN	THA	33.86
CAN	TON	9.23
CAN	TTO	0.08
CAN	TUN	161.14
CAN	GBR	558.32
CAN	USA	7599.74
CAN	URY	290402.00
CAN	UZB	16.32
CAN	VNM	517.78
CAN	ZMB	13.84
CPV	BEL	0.46
CPV	BRA	0.50
CPV	CAN	0.16
CPV	CIV	15.24
CPV	CZE	0.16
CPV	SLV	0.40
CPV	FRA	1.42
CPV	GAB	0.26
CPV	DEU	3.04
CPV	GIN	0.22
CPV	ITA	2.18
CPV	LBY	0.52
CPV	MRT	0.22
CPV	MOZ	1.22
CPV	NZL	7.92
CPV	QAT	17.20
CPV	SEN	1.04
CPV	SRB	2.46
CPV	ESP	0.22
CPV	LKA	5.50
CPV	CHE	0.66
CPV	GBR	0.18
CPV	USA	2.84
CPV	URY	2.32
CAF	BEL	39.88
CAF	CMR	13.50
CAF	CHN	0.22
CAF	COD	0.66
CAF	DNK	0.08
CAF	FRA	13.00
CAF	DEU	2.58
CAF	HKG	0.86
CAF	IRL	0.08
CAF	ISR	1.16
CAF	ITA	2.18
CAF	JPN	0.34
CAF	NZL	0.08
CAF	QAT	0.76
CAF	LKA	0.66
CAF	SUR	0.44
CAF	SYR	3.76
CAF	TON	0.06
CAF	TKM	0.62
CAF	USA	0.62
CAF	URY	0.16
CHL	ARG	679.93
CHL	BEL	548.20
CHL	BOL	170.88
CHL	BRA	2311.38
CHL	BGR	303.98
CHL	CAN	1247.12

CHL	CHN	5631.18
CHL	COL	441.73
CHL	CRI	137.66
CHL	ECU	417.28
CHL	SLV	97.24
CHL	FRA	1869.20
CHL	DEU	1312.73
CHL	GTM	125.20
CHL	HND	54.08
CHL	IND	1155.80
CHL	ITA	2315.85
CHL	JPN	5468.72
CHL	KOR	2818.23
CHL	MDA	1884.33
CHL	NZL	2919.68
CHL	NER	22.72
CHL	PNG	31.98
CHL	PER	53.24
CHL	PHL	804.03
CHL	STP	0.82
CHL	LKA	1102.73
CHL	CHE	322.18
CHL	TTO	0.32
CHL	TKM	314.60
CHL	USA	701.82
CHL	URY	6350.74
CHL	UZB	63.58
CHL	VNM	496.93
CHN	AFG	227.86
CHN	ALB	160.30
CHN	DZA	965.40
CHN	ATG	7.63
CHN	ARG	2373.02
CHN	ARM	69.72
CHN	AUS	16408.40
CHN	AUT	3872.88
CHN	AZE	182.60
CHN	BHR	264.33
CHN	BGD	1618.34
CHN	BRB	42.12
CHN	BLR	375.66
CHN	BEL	10996.10
CHN	BLZ	18.84
CHN	BEN	59.28
CHN	BOL	157.48
CHN	BIH	218.08
CHN	BWA	36.48
CHN	BRA	6363.98
CHN	BRN	83.33
CHN	BGR	652.60
CHN	BFA	25.98
CHN	BDI	10.32
CHN	KHM	206.02
CHN	CMR	121.50
CHN	CAN	24486.30
CHN	CPV	7.52
CHN	CAF	1.90
CHN	CHL	3187.93
CHN	COL	1819.20
CHN	COM	2.38
CHN	COD	22.80
CHN	CRI	417.68
CHN	CIV	224.58

CHN	HRV	933.10
CHN	CYP	289.82
CHN	CZE	5004.54
CHN	DNK	3669.36
CHN	DOM	3.58
CHN	ECU	751.42
CHN	EGY	990.36
CHN	SLV	193.32
CHN	ERI	10.68
CHN	EST	452.82
CHN	ETH	595.36
CHN	FJI	50.22
CHN	FIN	3786.80
CHN	FRA	26067.20
CHN	GAB	19.06
CHN	GMB	23.80
CHN	GEO	81.76
CHN	DEU	51452.60
CHN	GHA	450.06
CHN	GRC	2276.62
CHN	GRD	7.24
CHN	GTM	447.72
CHN	GUY	41.72
CHN	HND	118.84
CHN	HKG	135638.00
CHN	HUN	3749.44
CHN	ISL	224.56
CHN	IND	12726.20
CHN	IDN	8227.94
CHN	IRN	1589.00
CHN	IRL	4564.86
CHN	ISR	2043.90
CHN	ITA	19068.30
CHN	JAM	154.96
CHN	JPN	104947.00
CHN	JOR	921.04
CHN	KAZ	1593.12
CHN	KEN	545.55
CHN	KIR	1.70
CHN	KOR	40344.80
CHN	KGZ	172.34
CHN	LVA	166.34
CHN	LBN	476.20
CHN	LSO	25.64
CHN	LBY	120.60
CHN	LTU	425.30
CHN	LUX	87.68
CHN	MDG	125.46
CHN	MWI	297.60
CHN	MYS	29.72
CHN	MDV	13112.20
CHN	MLI	13.78
CHN	MLT	84.68
CHN	MRT	101.90
CHN	MUS	23.48
CHN	MEX	305.04
CHN	MDA	19130.40
CHN	MNG	90.56
CHN	MNE	350.86
CHN	MOZ	1081.62
CHN	MMR	64.56
CHN	NPL	56.96
CHN	NLD	117.90

CHN	NZL	26034.90
CHN	NIC	2805.32
CHN	NER	167.32
CHN	NGA	60.20
CHN	NOR	1656.57
CHN	OMN	3155.30
CHN	PAK	249.06
CHN	PAN	2374.92
CHN	PNG	168.23
CHN	PRY	33.14
CHN	PER	915.32
CHN	PHL	1305.12
CHN	POL	3200.78
CHN	PRT	6377.58
CHN	QAT	819.38
CHN	ROU	390.96
CHN	RUS	1565.94
CHN	RWA	14527.60
CHN	WSM	16.90
CHN	STP	5.12
CHN	SAU	0.06
CHN	SEN	4851.72
CHN	SRB	144.28
CHN	SYC	794.13
CHN	SLE	7.38
CHN	SVK	21378.30
CHN	SVN	1438.28
CHN	SLB	307.46
CHN	ESP	5236.30
CHN	LKA	15224.40
CHN	KNA	312.78
CHN	LCA	1.86
CHN	VCT	8.86
CHN	SDN	4.26
CHN	SUR	1092.30
CHN	SWZ	44.74
CHN	SWE	28.54
CHN	CHE	3287.34
CHN	SYR	2785.62
CHN	TJK	489.04
CHN	THA	247.54
CHN	TLS	11201.60
CHN	TGO	1.40
CHN	TON	67.98
CHN	TTO	5.58
CHN	TUN	198.12
CHN	TUR	400.82
CHN	TKM	7361.36
CHN	UKR	139.02
CHN	ARE	1737.30
CHN	GBR	9807.26
CHN	USA	32328.30
CHN	URY	255896.00
CHN	UZB	278.42
CHN	VEN	10.85
CHN	VNM	1027.60
CHN	YEM	6488.26
CHN	MKD	356.68
CHN	ZMB	98.52
CHN	ZWE	105.15
COL	BEL	328.64
COL	BOL	60.34
COL	BRA	207.48

COL	CAN	234.32
COL	CHL	470.83
COL	CHN	338.72
COL	CRI	261.72
COL	DOM	513.50
COL	ECU	1285.38
COL	SLV	70.12
COL	FRA	282.42
COL	DEU	356.32
COL	GTM	155.48
COL	GUY	6.34
COL	HND	54.96
COL	ISR	364.26
COL	ITA	424.14
COL	JAM	56.34
COL	JPN	302.58
COL	MDA	514.20
COL	NZL	495.28
COL	PNG	230.96
COL	PHL	798.56
COL	QAT	138.68
COL	LKA	448.38
COL	LCA	0.72
COL	VCT	4.66
COL	SDN	2.04
COL	SWZ	13.74
COL	SYR	373.36
COL	TUN	316.12
COL	USA	317.74
COL	URY	8522.26
COL	VNM	2465.38
COM	CAN	0.05
COM	ECU	0.03
COM	FRA	2.43
COM	DEU	0.25
COM	IND	0.15
COM	JPN	0.13
COM	MWI	0.03
COM	MEX	0.13
COM	STP	1.38
COM	SVK	0.13
COM	GBR	0.10
COM	URY	0.18
COM	MKD	0.10
COD	AGO	9.03
COD	BRA	40.78
COD	BFA	6.50
COD	CMR	21.20
COD	CAF	3.03
COD	CHL	18.93
COD	CHN	186.75
COD	CIV	7.58
COD	FRA	87.65
COD	GAB	5.62
COD	DEU	40.45
COD	GHA	36.52
COD	ISL	45.60
COD	IND	19.70
COD	IDN	23.10
COD	ISR	15.03
COD	ITA	29.08
COD	JPN	24.88
COD	KOR	164.38

COD	NZL	17.80
COD	QAT	24.98
COD	WSM	3.76
COD	SRB	6.68
COD	SVK	13.10
COD	LKA	20.08
COD	TUN	267.84
COD	USA	8.20
COD	URY	279.63
COD	ZMB	41.70
CRI	BEL	130.42
CRI	BLZ	4.72
CRI	CAN	51.10
CRI	CHN	376.80
CRI	COL	37.74
CRI	DOM	107.84
CRI	SLV	186.06
CRI	FRA	36.02
CRI	DEU	201.30
CRI	GTM	291.60
CRI	GUY	3.90
CRI	HND	190.14
CRI	HKG	367.50
CRI	IRL	42.84
CRI	ITA	84.96
CRI	JAM	47.86
CRI	JPN	57.14
CRI	KOR	42.40
CRI	MDV	142.44
CRI	MDA	769.82
CRI	NZL	421.88
CRI	NER	232.70
CRI	PNG	229.68
CRI	CHE	68.76
CRI	USA	147.82
CRI	URY	2957.88
CIV	DZA	63.04
CIV	BEL	186.78
CIV	BEN	104.40
CIV	BFA	234.64
CIV	CMR	37.56
CIV	CAN	61.82
CIV	CPV	4.10
CIV	CAF	0.48
CIV	CHN	69.32
CIV	COD	64.50
CIV	GNQ	94.84
CIV	FRA	1415.54
CIV	GAB	9.76
CIV	GMB	22.04
CIV	DEU	176.46
CIV	GHA	151.12
CIV	GIN	60.68
CIV	IND	126.10
CIV	ITA	260.20
CIV	MLT	184.58
CIV	NZL	801.84
CIV	NGA	66.72
CIV	NOR	494.26
CIV	PNG	104.42
CIV	PRT	81.54
CIV	RWA	74.00
CIV	SRB	112.08

CIV	LKA	246.76
CIV	TON	93.76
CIV	USA	204.68
CIV	URY	673.78
HRV	ALB	34.78
HRV	AUT	694.94
HRV	BIH	1279.08
HRV	CZE	85.38
HRV	FRA	209.16
HRV	DEU	974.58
HRV	HUN	152.56
HRV	ITA	2020.60
HRV	JPN	69.02
HRV	LBR	131.92
HRV	MDG	85.24
HRV	MRT	126.92
HRV	NZL	83.84
HRV	PRT	70.54
HRV	RUS	60.38
HRV	RWA	116.44
HRV	SYC	608.15
HRV	SLB	794.10
HRV	CHE	122.14
HRV	SYR	93.24
HRV	TKM	61.50
HRV	GBR	98.54
HRV	USA	145.26
HRV	URY	271.00
CYP	BEL	15.70
CYP	CZE	8.60
CYP	EGY	12.55
CYP	FRA	101.83
CYP	DEU	74.00
CYP	GRC	199.30
CYP	IRL	13.00
CYP	ISR	14.90
CYP	ITA	30.53
CYP	JPN	20.40
CYP	JOR	16.10
CYP	LBN	33.65
CYP	NPL	8.40
CYP	NZL	25.53
CYP	RUS	23.68
CYP	RWA	21.30
CYP	SEN	10.38
CYP	LKA	8.23
CYP	CHE	8.93
CYP	TJK	17.34
CYP	GBR	41.48
CYP	USA	248.15
CYP	URY	15.82
CZE	ALB	25.00
CZE	AUT	4324.16
CZE	BLR	124.30
CZE	BEL	2199.12
CZE	BIH	112.16
CZE	BGR	328.32
CZE	CHN	381.96
CZE	HRV	445.32
CZE	DNK	646.40
CZE	EST	118.24
CZE	FIN	446.18
CZE	FRA	4259.12

CZE	GEO	27.00
CZE	DEU	27124.00
CZE	HUN	2349.24
CZE	ISL	37.52
CZE	ITA	3713.82
CZE	KAZ	106.16
CZE	LVA	156.68
CZE	LTU	245.58
CZE	LUX	82.68
CZE	MNG	28.76
CZE	MNE	5.52
CZE	NZL	3123.92
CZE	OMN	452.92
CZE	PRT	4516.76
CZE	RUS	1018.52
CZE	RWA	1537.62
CZE	SYC	231.98
CZE	SVN	6911.48
CZE	SLB	488.70
CZE	LKA	2032.50
CZE	CHE	1238.18
CZE	SYR	1130.54
CZE	TKM	530.84
CZE	ARE	715.72
CZE	GBR	428.84
CZE	USA	3996.62
CZE	URY	1861.20
DNK	AUS	628.00
DNK	AUT	683.80
DNK	BHS	1.50
DNK	BRB	6.34
DNK	BLR	65.18
DNK	BEL	1221.00
DNK	BLZ	2.16
DNK	BEN	5.54
DNK	BFA	10.32
DNK	BDI	4.32
DNK	CAN	697.42
DNK	CAF	0.65
DNK	CHN	1109.50
DNK	CZE	625.70
DNK	ERI	4.55
DNK	EST	235.94
DNK	ETH	40.58
DNK	FJI	1.94
DNK	FIN	2395.24
DNK	FRA	3788.82
DNK	GMB	27.42
DNK	DEU	13801.20
DNK	GRC	626.68
DNK	GRD	1.58
DNK	HKG	788.26
DNK	ISL	345.00
DNK	IRL	1221.22
DNK	ITA	2634.12
DNK	JPN	1801.92
DNK	LVA	272.30
DNK	LTU	482.90
DNK	LUX	37.78
DNK	MYS	16.78
DNK	MLI	3.74
DNK	MRT	34.52
DNK	MNE	3.48

DNK	NZL	4042.72
DNK	NIC	142.92
DNK	OMN	4677.78
DNK	PRT	1556.32
DNK	QAT	597.62
DNK	RWA	1251.90
DNK	WSM	5.80
DNK	STP	1.30
DNK	SAU	0.16
DNK	SLE	3.26
DNK	LKA	2336.82
DNK	LCA	1.26
DNK	SDN	1.02
DNK	CHE	11118.60
DNK	SYR	874.84
DNK	TGO	1.05
DNK	UKR	18.62
DNK	USA	6765.02
DNK	URY	4928.92
DNK	ZMB	13.22
DJI	ERI	8.50
DJI	ETH	56.76
DOM	ATG	4.78
DOM	BHS	2.15
DOM	BRB	1.98
DOM	BLZ	0.48
DOM	CAN	0.04
DOM	FRA	3.80
DOM	DEU	0.04
DOM	GRD	0.30
DOM	GUY	4.28
DOM	HTI	0.06
DOM	JAM	28.66
DOM	LCA	1.82
DOM	VCT	3.10
DOM	SDN	1.08
DOM	SWZ	0.74
DOM	TUN	3.10
DOM	USA	6.68
DOM	URY	1.86
DOM	VNM	0.02
ECU	ARG	45.28
ECU	BEL	99.36
ECU	BOL	10.52
ECU	BRA	49.14
ECU	CAN	49.44
ECU	CHL	443.48
ECU	CHN	60.30
ECU	COL	549.70
ECU	SLV	145.60
ECU	FRA	91.36
ECU	DEU	212.04
ECU	GTM	130.92
ECU	HND	52.12
ECU	ITA	397.56
ECU	JAM	36.20
ECU	JPN	92.46
ECU	KOR	91.50
ECU	MDA	57.14
ECU	NZL	177.34
ECU	NER	70.46
ECU	PNG	562.60
ECU	PHL	968.06

ECU	RWA	316.28
ECU	LKA	219.20
ECU	USA	72.34
ECU	URY	4692.10
ECU	VNM	219.58
EGY	DZA	140.54
EGY	BEL	173.16
EGY	BDI	2.66
EGY	CHN	114.36
EGY	COM	1.00
EGY	CYP	68.00
EGY	ERI	5.73
EGY	ETH	63.82
EGY	FRA	399.92
EGY	DEU	135.14
EGY	GRC	122.52
EGY	IND	918.32
EGY	ITA	1134.54
EGY	JPN	194.98
EGY	JOR	371.12
EGY	KEN	139.95
EGY	KOR	213.80
EGY	LBN	222.36
EGY	LBY	149.36
EGY	MRT	40.98
EGY	MUS	7.92
EGY	MOZ	93.40
EGY	NZL	379.08
EGY	WSM	5.84
EGY	SEN	677.32
EGY	SRB	27.74
EGY	SVK	130.16
EGY	LKA	720.16
EGY	SUR	340.56
EGY	TJK	254.90
EGY	TUR	98.14
EGY	TKM	304.08
EGY	GBR	209.98
EGY	USA	265.78
EGY	URY	839.68
EGY	MKD	96.86
SLV	BEL	13.58
SLV	BLZ	10.28
SLV	CAN	13.08
SLV	CHN	5.36
SLV	CRI	102.54
SLV	DOM	39.44
SLV	FRA	9.82
SLV	DEU	47.06
SLV	GTM	419.52
SLV	HND	231.66
SLV	ITA	6.42
SLV	JAM	3.50
SLV	JPN	12.54
SLV	MDV	3.36
SLV	MDA	39.08
SLV	NZL	5.70
SLV	NER	131.48
SLV	PNG	59.25
SLV	RWA	25.08
SLV	LKA	53.42
SLV	USA	5.58
SLV	URY	353.40

SLV	VNM	4.02
GNQ	CMR	44.54
GNQ	UZB	32.58
ERI	AUS	0.10
ERI	CMR	0.08
ERI	DJI	0.20
ERI	FRA	0.50
ERI	DEU	0.45
ERI	GRC	0.15
ERI	HKG	0.05
ERI	IND	1.20
ERI	ISR	0.30
ERI	ITA	1.35
ERI	KEN	0.05
ERI	MRT	0.18
ERI	NZL	0.60
ERI	PAN	0.20
ERI	RWA	0.05
ERI	SEN	0.55
ERI	SVK	0.35
ERI	ESP	0.10
ERI	KNA	0.13
ERI	SUR	15.95
ERI	GBR	0.10
ERI	USA	0.63
ERI	URY	0.10
ERI	YEM	0.13
ERI	MKD	0.25
EST	AUT	40.98
EST	BEL	87.42
EST	CAN	47.32
EST	CHN	93.80
EST	DNK	234.36
EST	FIN	1673.78
EST	FRA	108.64
EST	DEU	501.58
EST	HUN	134.42
EST	ISL	55.84
EST	ITA	73.44
EST	LVA	728.24
EST	LTU	487.60
EST	NZL	204.10
EST	OMN	242.72
EST	PRT	94.78
EST	RWA	643.08
EST	LKA	47.90
EST	CHE	1051.76
EST	TON	92.56
EST	TKM	71.80
EST	ARE	143.48
EST	USA	247.16
EST	URY	330.50
ETH	BEL	27.82
ETH	CHN	56.28
ETH	DJI	58.66
ETH	EGY	7.18
ETH	FRA	16.60
ETH	DEU	102.00
ETH	GRC	5.52
ETH	IND	10.20
ETH	ISR	22.24
ETH	ITA	52.64
ETH	JPN	67.52

ETH	JOR	7.02
ETH	NZL	35.34
ETH	PAN	5.58
ETH	QAT	7.46
ETH	SEN	57.56
ETH	SVK	5.38
ETH	SUR	25.08
ETH	SYR	50.48
ETH	TKM	16.70
ETH	GBR	25.44
ETH	USA	24.86
ETH	URY	52.00
ETH	MKD	21.48
FJI	AUS	131.68
FJI	CAN	2.06
FJI	CHN	3.14
FJI	DEU	1.74
FJI	HKG	8.48
FJI	JPN	35.60
FJI	KIR	15.50
FJI	NIC	37.16
FJI	PRY	4.06
FJI	QAT	1.88
FJI	STP	14.00
FJI	SVK	58.12
FJI	ZAF	2.50
FJI	TTO	21.56
FJI	USA	94.60
FJI	URY	117.44
FJI	VEN	16.30
FIN	DZA	124.88
FIN	ATG	5.67
FIN	AUS	514.16
FIN	AUT	1016.46
FIN	AZE	71.88
FIN	BEL	1897.14
FIN	KHM	2.86
FIN	CMR	19.18
FIN	CAN	634.36
FIN	CHL	305.90
FIN	CHN	2243.98
FIN	COL	154.22
FIN	CYP	83.26
FIN	DNK	1704.00
FIN	ECU	65.66
FIN	EST	1856.36
FIN	FRA	2331.60
FIN	DEU	9044.24
FIN	GRC	593.78
FIN	GRD	1.84
FIN	HND	29.60
FIN	HUN	588.10
FIN	ISL	67.98
FIN	ISR	289.70
FIN	ITA	2123.48
FIN	JPN	1109.24
FIN	JOR	140.82
FIN	KAZ	197.94
FIN	KGZ	11.78
FIN	LVA	553.44
FIN	LTU	470.82
FIN	LUX	41.16
FIN	MEX	67.16

FIN	MNE	3.96
FIN	NPL	5.32
FIN	NZL	3454.56
FIN	OMN	1993.96
FIN	PRT	1465.20
FIN	RWA	6634.96
FIN	SEN	828.72
FIN	LKA	1759.28
FIN	VCT	3.94
FIN	CHE	7270.56
FIN	SYR	785.44
FIN	THA	38.18
FIN	TUR	89.64
FIN	TKM	686.08
FIN	ARE	371.12
FIN	GBR	1212.26
FIN	USA	4680.46
FIN	URY	4418.42
FIN	ZMB	26.00
FRA	AFG	18.80
FRA	ALB	32.00
FRA	DZA	5200.92
FRA	ATG	8.07
FRA	ARG	718.00
FRA	ARM	57.92
FRA	AUS	3275.22
FRA	AUT	4568.04
FRA	AZE	111.50
FRA	BHS	15.30
FRA	BHR	176.77
FRA	BRB	24.58
FRA	BLR	214.80
FRA	BEL	37756.90
FRA	BEN	177.72
FRA	BOL	26.94
FRA	BIH	134.26
FRA	BWA	20.98
FRA	BRA	2628.74
FRA	BRN	17.87
FRA	BGR	844.12
FRA	BFA	147.62
FRA	BDI	13.18
FRA	KHM	42.40
FRA	CMR	487.10
FRA	CAN	4238.72
FRA	CPV	23.58
FRA	CAF	27.10
FRA	CHL	673.58
FRA	CHN	9483.08
FRA	COL	405.86
FRA	COM	18.38
FRA	COD	191.30
FRA	CRI	172.86
FRA	CIV	1450.52
FRA	HRV	808.18
FRA	CYP	316.64
FRA	CZE	3799.46
FRA	DNK	3345.66
FRA	DOM	4.58
FRA	ECU	69.68
FRA	EGY	532.80
FRA	SLV	57.32
FRA	ERI	10.30

FRA	EST	286.74
FRA	ETH	92.96
FRA	FJI	12.76
FRA	FIN	2307.68
FRA	GAB	482.68
FRA	GMB	11.22
FRA	GEO	77.14
FRA	DEU	70750.80
FRA	GHA	162.40
FRA	GRC	4103.18
FRA	GRD	3.86
FRA	GTM	65.84
FRA	HKG	2533.18
FRA	HUN	3226.92
FRA	ISL	160.26
FRA	IND	2763.04
FRA	IDN	822.10
FRA	IRN	2134.90
FRA	IRL	3305.28
FRA	ISR	1283.56
FRA	ITA	40936.90
FRA	JAM	37.98
FRA	JPN	8633.78
FRA	JOR	252.28
FRA	KAZ	392.52
FRA	KEN	196.40
FRA	KIR	0.10
FRA	KOR	2956.72
FRA	KGZ	11.68
FRA	LVA	223.48
FRA	LBN	594.52
FRA	LSO	0.62
FRA	LBY	244.60
FRA	LTU	540.58
FRA	LUX	2236.50
FRA	MDG	67.18
FRA	MWI	231.34
FRA	MYS	14.20
FRA	MDV	1700.20
FRA	MLI	13.96
FRA	MLT	248.74
FRA	MRT	457.46
FRA	MUS	130.12
FRA	MEX	343.44
FRA	MDA	2552.26
FRA	MNG	61.96
FRA	MNE	17.20
FRA	MOZ	3834.06
FRA	MMR	28.58
FRA	NPL	28.46
FRA	NLD	21.97
FRA	NZL	18142.00
FRA	NIC	581.06
FRA	NER	16.94
FRA	NGA	122.24
FRA	NOR	624.27
FRA	OMN	2284.90
FRA	PAK	222.68
FRA	PAN	286.36
FRA	PNG	31.83
FRA	PRY	3.82
FRA	PER	36.62
FRA	PHL	151.30

FRA	POL	419.82
FRA	PRT	6560.66
FRA	QAT	5789.14
FRA	ROU	599.54
FRA	RUS	2902.42
FRA	RWA	4985.08
FRA	WSM	15.16
FRA	SAU	0.34
FRA	SEN	2145.36
FRA	SRB	803.88
FRA	SYC	390.05
FRA	SLE	53.18
FRA	SVK	4402.96
FRA	SVN	1378.34
FRA	SLB	1454.24
FRA	ESP	2509.76
FRA	LKA	44205.90
FRA	KNA	112.98
FRA	LCA	1.26
FRA	VCT	8.82
FRA	SDN	2.64
FRA	SUR	176.54
FRA	SWZ	4.40
FRA	SWE	1.38
FRA	CHE	5924.90
FRA	SYR	13123.30
FRA	TJK	204.60
FRA	THA	68.66
FRA	TLS	1342.42
FRA	TON	120.70
FRA	TTO	0.42
FRA	TUN	52.06
FRA	TUR	3517.66
FRA	TKM	6262.92
FRA	UKR	45.04
FRA	ARE	860.76
FRA	GBR	3828.60
FRA	USA	39451.20
FRA	URY	35572.30
FRA	UZB	67.32
FRA	VEN	6.55
FRA	VNM	286.94
FRA	YEM	439.48
FRA	MKD	121.26
FRA	ZMB	66.30
FRA	ZWE	11.88
GAB	AUS	16.14
GAB	BRA	11.64
GAB	CAF	0.78
GAB	CHN	236.88
GAB	COD	11.02
GAB	FRA	231.84
GAB	GIN	10.28
GAB	ISL	32.94
GAB	IND	73.28
GAB	ITA	47.68
GAB	MOZ	15.66
GAB	NZL	16.36
GAB	NOR	11.90
GAB	OMN	21.16
GAB	SAU	0.62
GAB	SRB	14.06
GAB	SVK	85.02

GAB	ESP	61.32
GAB	LKA	49.50
GAB	SYR	104.58
GAB	TLS	43.06
GAB	TUN	235.72
GAB	ARE	16.54
GAB	USA	17.06
GAB	URY	1936.62
GAB	UZB	9.92
GMB	BEL	0.18
GMB	CHN	0.14
GMB	DNK	0.82
GMB	FRA	0.50
GMB	DEU	0.36
GMB	GHA	0.04
GMB	GRC	0.18
GMB	GIN	0.48
GMB	IND	0.06
GMB	ITA	0.18
GMB	JPN	0.04
GMB	LSO	0.48
GMB	MLT	0.26
GMB	MUS	0.22
GMB	MOZ	0.04
GMB	NZL	0.92
GMB	PRY	2.08
GMB	QAT	0.08
GMB	SRB	1.50
GMB	SGP	0.04
GMB	ESP	0.04
GMB	LKA	0.12
GMB	SYR	0.08
GMB	TLS	0.04
GMB	USA	3.72
GMB	URY	0.12
GEO	ARM	61.90
GEO	AZE	71.00
GEO	BEL	8.40
GEO	BGR	36.06
GEO	CAN	31.80
GEO	CHN	5.76
GEO	CZE	4.28
GEO	FRA	13.72
GEO	DEU	31.14
GEO	GRC	7.08
GEO	IND	6.30
GEO	ITA	19.14
GEO	KAZ	14.28
GEO	NZL	9.96
GEO	RUS	4.86
GEO	RWA	92.74
GEO	SLE	4.38
GEO	LKA	12.34
GEO	SYR	11.54
GEO	TKM	123.94
GEO	UGA	69.48
GEO	ARE	46.80
GEO	GBR	9.84
GEO	USA	27.80
GEO	URY	54.38
DEU	AFG	78.28
DEU	ALB	158.62
DEU	DZA	1144.22

DEU	ATG	3.90
DEU	ARG	1374.60
DEU	ARM	80.12
DEU	AUS	6543.74
DEU	AUT	55430.90
DEU	AZE	299.94
DEU	BHS	5.60
DEU	BHR	293.90
DEU	BGD	301.94
DEU	BRB	23.98
DEU	BLR	1372.82
DEU	BEL	57208.30
DEU	BLZ	3.16
DEU	BEN	18.92
DEU	BOL	53.54
DEU	BIH	869.24
DEU	BWA	24.12
DEU	BRA	6119.60
DEU	BRN	40.97
DEU	BGR	2548.92
DEU	BFA	37.30
DEU	BDI	8.18
DEU	KHM	9.22
DEU	CMR	97.66
DEU	CAN	8502.20
DEU	CPV	12.18
DEU	CAF	1.70
DEU	CHL	1204.28
DEU	CHN	33726.70
DEU	COL	842.00
DEU	COD	35.80
DEU	CRI	174.20
DEU	CIV	131.24
DEU	HRV	2877.42
DEU	CYP	562.36
DEU	CZE	25772.00
DEU	DNK	16483.70
DEU	DOM	1.56
DEU	ECU	225.30
DEU	EGY	1095.32
DEU	SLV	110.86
DEU	ERI	11.25
DEU	EST	1419.72
DEU	ETH	152.70
DEU	FJI	10.70
DEU	FIN	10550.00
DEU	FRA	101301.00
DEU	GAB	24.40
DEU	GMB	32.64
DEU	GEO	235.74
DEU	GHA	432.10
DEU	GRC	8477.28
DEU	GRD	5.08
DEU	GTM	189.24
DEU	GUY	6.52
DEU	HND	78.82
DEU	HKG	5534.40
DEU	HUN	18804.40
DEU	ISL	606.46
DEU	IND	5821.20
DEU	IDN	1626.92
DEU	IRN	3877.53
DEU	IRL	5586.34

DEU	ISR	3098.58
DEU	ITA	70281.50
DEU	JAM	53.52
DEU	JPN	17397.20
DEU	JOR	748.34
DEU	KAZ	1496.82
DEU	KEN	296.00
DEU	KOR	9995.26
DEU	KGZ	44.48
DEU	LVA	1420.34
DEU	LBN	606.46
DEU	LSO	5.14
DEU	LBY	562.47
DEU	LTU	2506.84
DEU	LUX	4719.20
DEU	MDG	379.80
DEU	MWI	46.66
DEU	MYS	19.82
DEU	MDV	5235.54
DEU	MLI	14.26
DEU	MLT	43.14
DEU	MRT	326.22
DEU	MUS	32.94
DEU	MEX	112.10
DEU	MDA	8431.22
DEU	MNG	202.08
DEU	MNE	44.56
DEU	MOZ	1078.22
DEU	MMR	34.58
DEU	NPL	54.44
DEU	NLD	22.40
DEU	NZL	64094.70
DEU	NIC	1207.72
DEU	NER	41.32
DEU	NGA	14.26
DEU	NOR	966.23
DEU	OMN	7770.90
DEU	PAK	486.96
DEU	PAN	949.08
DEU	PNG	61.58
DEU	PRY	5.40
DEU	PER	77.50
DEU	PHL	424.30
DEU	POL	1236.42
DEU	PRT	31140.20
DEU	QAT	8948.12
DEU	ROU	705.70
DEU	RUS	6773.02
DEU	RWA	24400.50
DEU	WSM	17.06
DEU	STP	0.38
DEU	SAU	0.20
DEU	SEN	5091.52
DEU	SRB	94.82
DEU	SYC	1495.58
DEU	SLE	38.16
DEU	SVK	6284.60
DEU	SVN	8309.02
DEU	SLB	4113.94
DEU	ESP	8113.32
DEU	LKA	50177.10
DEU	KNA	154.98
DEU	LCA	1.78

DEU	VCT	6.68
DEU	SDN	2.40
DEU	SUR	338.48
DEU	SWZ	11.54
DEU	SWE	1.90
DEU	CHE	22138.40
DEU	SYR	41287.00
DEU	TJK	243.84
DEU	THA	108.84
DEU	TLS	3156.02
DEU	TGO	0.05
DEU	TON	19.23
DEU	TTO	0.06
DEU	TUN	221.66
DEU	TUR	1168.18
DEU	TKM	15884.00
DEU	UKR	56.26
DEU	ARE	3695.12
DEU	GBR	3931.24
DEU	USA	78414.20
DEU	URY	86966.70
DEU	UZB	88.66
DEU	VEN	0.35
DEU	VNM	566.00
DEU	YEM	688.68
DEU	MKD	167.62
DEU	ZMB	41.40
DEU	ZWE	48.50
GHA	AUS	14.62
GHA	BEL	132.78
GHA	BEN	50.56
GHA	BFA	124.54
GHA	CAN	15.06
GHA	CHN	33.12
GHA	CIV	14.42
GHA	DNK	18.82
GHA	EST	33.82
GHA	FRA	130.82
GHA	DEU	79.70
GHA	IND	40.56
GHA	ITA	74.96
GHA	JPN	63.90
GHA	MDV	59.08
GHA	MLT	42.34
GHA	NZL	436.74
GHA	NGA	12.14
GHA	NOR	116.06
GHA	SRB	12.88
GHA	ESP	641.60
GHA	LKA	52.28
GHA	SYR	243.48
GHA	TON	14.93
GHA	TKM	35.24
GHA	USA	255.18
GHA	URY	155.44
GRC	ALB	464.58
GRC	ARM	38.26
GRC	AUT	159.96
GRC	BEL	266.76
GRC	BGR	1131.82
GRC	COM	0.34
GRC	CYP	1048.96
GRC	DNK	146.30

GRC	FRA	770.82
GRC	GEO	23.66
GRC	DEU	2203.14
GRC	ISR	166.56
GRC	ITA	1942.50
GRC	LBN	104.68
GRC	LBY	181.98
GRC	MDG	431.34
GRC	MRT	25.84
GRC	MUS	7.08
GRC	MNG	14.16
GRC	NZL	421.88
GRC	PRT	213.52
GRC	RUS	629.68
GRC	RWA	411.44
GRC	SYC	209.28
GRC	SLB	128.54
GRC	LKA	654.24
GRC	CHE	184.74
GRC	SYR	137.66
GRC	TKM	814.62
GRC	GBR	199.96
GRC	USA	1170.22
GRC	URY	897.72
GRD	ATG	0.94
GRD	ARG	0.46
GRD	BRB	1.64
GRD	BEL	1.40
GRD	CAN	0.60
GRD	COL	0.06
GRD	DOM	1.48
GRD	FRA	1.28
GRD	DEU	1.30
GRD	GUY	0.46
GRD	JAM	0.60
GRD	JPN	0.36
GRD	NZL	4.30
GRD	NER	0.06
GRD	LKA	0.08
GRD	LCA	1.68
GRD	VCT	2.96
GRD	SDN	0.70
GRD	CHE	0.72
GRD	TUN	1.64
GRD	USA	0.52
GRD	URY	8.50
GTM	BEL	21.88
GTM	BLZ	38.46
GTM	CAN	73.02
GTM	CHN	29.58
GTM	CRI	183.38
GTM	DOM	52.82
GTM	SLV	606.04
GTM	DEU	65.56
GTM	HND	384.86
GTM	ITA	30.20
GTM	JAM	15.66
GTM	JPN	36.90
GTM	KOR	47.36
GTM	MDA	218.76
GTM	NZL	48.50
GTM	NER	189.96
GTM	PNG	96.64

GTM	PHL	14.98
GTM	RWA	16.64
GTM	SEN	37.74
GTM	LKA	22.24
GTM	SYR	44.86
GTM	URY	1657.42
GTM	VNM	20.28
GUY	ATG	4.50
GUY	BRB	26.32
GUY	BEL	36.12
GUY	CAN	110.70
GUY	CHN	6.08
GUY	DOM	2.02
GUY	FRA	6.02
GUY	GRD	3.18
GUY	HTI	4.04
GUY	HKG	2.48
GUY	IND	5.24
GUY	ISR	5.22
GUY	ITA	2.34
GUY	JAM	33.00
GUY	NZL	26.60
GUY	QAT	16.88
GUY	LKA	2.28
GUY	LCA	0.84
GUY	VCT	4.66
GUY	SDN	3.10
GUY	SWZ	8.38
GUY	TUN	34.32
GUY	ARE	5.84
GUY	USA	103.16
GUY	URY	93.42
HND	BHS	2.10
HND	BRB	6.12
HND	BEL	70.20
HND	BLZ	6.30
HND	CAN	8.02
HND	CHN	12.42
HND	CRI	25.40
HND	DOM	7.72
HND	SLV	144.14
HND	FRA	13.10
HND	DEU	119.58
HND	GRD	4.50
HND	GTM	131.82
HND	ITA	17.70
HND	JAM	11.86
HND	JPN	16.92
HND	KOR	14.28
HND	MDA	58.34
HND	NZL	36.42
HND	NER	55.82
HND	PNG	10.16
HND	QAT	6.18
HND	LKA	27.46
HND	VCT	3.18
HND	SDN	1.12
HND	CHE	6.92
HND	USA	12.30
HND	URY	700.94
HKG	AUS	3596.56
HKG	BHS	3.70
HKG	BGD	468.82

HKG	BEL	1740.32
HKG	BLZ	2.58
HKG	BWA	15.06
HKG	BRN	66.13
HKG	KHM	349.56
HKG	CAN	3276.72
HKG	CHN	131655.00
HKG	COD	4.98
HKG	DNK	396.28
HKG	FJI	27.80
HKG	FRA	3834.16
HKG	GMB	6.90
HKG	DEU	8970.24
HKG	GTM	77.36
HKG	HUN	943.78
HKG	IND	3087.18
HKG	IDN	1322.96
HKG	IRL	519.18
HKG	ISR	1324.78
HKG	ITA	3398.72
HKG	JPN	14436.00
HKG	KEN	39.88
HKG	KIR	0.40
HKG	KOR	7102.64
HKG	LSO	39.36
HKG	LUX	99.44
HKG	MWI	23.48
HKG	MYS	7.36
HKG	MDV	3121.60
HKG	MLI	8.62
HKG	MRT	17.64
HKG	MDA	1216.52
HKG	MNE	20.50
HKG	NPL	6.14
HKG	NLD	55.73
HKG	NZL	4861.06
HKG	NOR	175.17
HKG	PRY	10.14
HKG	POL	2585.80
HKG	STP	1.26
HKG	SVK	5846.34
HKG	LKA	2120.86
HKG	KNA	629.72
HKG	SWZ	4.92
HKG	SWE	19.88
HKG	CHE	1068.58
HKG	SYR	2312.02
HKG	TLS	3150.30
HKG	TGO	0.35
HKG	TON	10.15
HKG	TTO	0.34
HKG	UKR	21.46
HKG	GBR	2074.66
HKG	USA	10126.60
HKG	URY	45468.40
HKG	VEN	2.45
HKG	VNM	140.06
HKG	YEM	1512.50
HKG	ZWE	10.75
HUN	ALB	22.72
HUN	AUT	3770.72
HUN	BLR	83.22
HUN	BEL	1290.68

HUN	BIH	240.48
HUN	BGR	445.26
HUN	CHN	549.48
HUN	HRV	908.40
HUN	CZE	2038.74
HUN	DNK	473.96
HUN	EST	111.22
HUN	FIN	602.70
HUN	FRA	3340.44
HUN	DEU	19907.60
HUN	ITA	3664.56
HUN	JOR	100.82
HUN	LVA	95.54
HUN	LTU	142.04
HUN	MDG	37.88
HUN	MEX	49.42
HUN	MNG	31.14
HUN	MNE	4.22
HUN	NZL	2250.52
HUN	OMN	324.78
HUN	PRT	2310.08
HUN	RUS	2474.32
HUN	RWA	1538.18
HUN	SYC	401.43
HUN	SVN	2110.64
HUN	SLB	740.90
HUN	LKA	2038.10
HUN	CHE	1071.00
HUN	SYR	734.26
HUN	TKM	850.48
HUN	UKR	18.24
HUN	ARE	998.52
HUN	GBR	518.34
HUN	USA	3166.60
HUN	URY	1826.02
ISL	BEL	60.74
ISL	CAN	23.14
ISL	CHN	27.26
ISL	DNK	132.40
ISL	FIN	14.38
ISL	FRA	112.94
ISL	DEU	512.08
ISL	GRC	28.56
ISL	IRL	84.44
ISL	ITA	29.92
ISL	JPN	106.52
ISL	LTU	34.74
ISL	MUS	4.82
ISL	NZL	508.96
ISL	NOR	50.40
ISL	OMN	118.34
ISL	PRT	33.66
ISL	QAT	92.58
ISL	RWA	41.80
ISL	LKA	203.40
ISL	CHE	32.18
ISL	SYR	54.76
ISL	GBR	22.88
ISL	USA	535.02
ISL	URY	299.14
IND	AFG	66.90
IND	DZA	231.46
IND	ARG	237.08

IND	AZE	53.54
IND	BHR	180.93
IND	BGD	1734.44
IND	BEL	3231.90
IND	BEN	12.12
IND	BOL	13.10
IND	BWA	19.12
IND	BRA	1176.72
IND	BRN	7.63
IND	BFA	10.30
IND	BDI	10.52
IND	KHM	13.24
IND	CMR	35.62
IND	CAN	1449.92
IND	CHN	9318.22
IND	COL	273.46
IND	COM	3.12
IND	COD	10.40
IND	CIV	96.22
IND	DNK	506.00
IND	EGY	308.46
IND	ERI	16.65
IND	ETH	265.18
IND	FJI	28.08
IND	FRA	1912.82
IND	GAB	8.14
IND	GMB	6.24
IND	DEU	3492.88
IND	GHA	199.76
IND	GUY	9.74
IND	HKG	4478.76
IND	IDN	1537.28
IND	IRN	1319.38
IND	ISR	1279.24
IND	ITA	2751.12
IND	JPN	2460.52
IND	JOR	170.48
IND	KAZ	111.62
IND	KEN	684.40
IND	KIR	0.20
IND	KOR	2692.00
IND	KGZ	5.30
IND	LBN	79.00
IND	LSO	3.32
IND	LBY	66.10
IND	MWI	67.12
IND	MYS	54.10
IND	MDV	1275.80
IND	MLI	82.14
IND	MLT	44.08
IND	MRT	19.92
IND	MEX	397.56
IND	MDA	944.72
IND	MOZ	187.32
IND	MMR	92.80
IND	NPL	18.88
IND	NLD	716.83
IND	NZL	2457.40
IND	NIC	149.50
IND	NER	20.22
IND	NGA	25.48
IND	NOR	599.17
IND	PAK	534.48

IND	PAN	727.70
IND	PRY	9.36
IND	PER	20.96
IND	PHL	131.50
IND	POL	389.04
IND	ROU	251.00
IND	WSM	15.30
IND	STP	0.30
IND	SEN	2019.78
IND	SRB	102.54
IND	SLE	14.06
IND	SVK	4756.96
IND	ESP	1477.90
IND	LKA	1577.48
IND	KNA	1905.04
IND	SUR	307.12
IND	SWZ	9.40
IND	SWE	2.50
IND	TJK	198.12
IND	THA	266.10
IND	TLS	1397.94
IND	TGO	0.95
IND	TON	12.15
IND	TTO	0.14
IND	TUN	37.04
IND	TUR	112.48
IND	TKM	1381.88
IND	UKR	181.96
IND	ARE	308.14
IND	GBR	9414.32
IND	USA	5179.76
IND	URY	19634.00
IND	UZB	31.36
IND	VEN	0.95
IND	YEM	570.32
IND	MKD	198.00
IND	ZMB	82.94
IND	ZWE	30.40
IDN	AUS	3129.10
IDN	BHR	31.07
IDN	BGD	446.64
IDN	BEL	1056.86
IDN	BEN	6.32
IDN	BRA	477.86
IDN	BRN	38.77
IDN	KHM	76.90
IDN	CHN	8680.38
IDN	COM	0.92
IDN	COD	2.80
IDN	EGY	207.78
IDN	ERI	4.73
IDN	ETH	56.36
IDN	FJI	20.06
IDN	FRA	711.02
IDN	DEU	1838.94
IDN	GHA	70.64
IDN	HKG	1854.10
IDN	IND	3326.66
IDN	ITA	1075.18
IDN	JPN	21327.80
IDN	JOR	134.24
IDN	KEN	234.85
IDN	KIR	0.50

IDN	KOR	7545.44
IDN	LSO	1.32
IDN	LUX	41.46
IDN	MDG	38.76
IDN	MWI	26.38
IDN	MYS	6.74
IDN	MDV	4538.62
IDN	MLI	17.64
IDN	MEX	66.78
IDN	MMR	19.26
IDN	NLD	37.37
IDN	NZL	2140.08
IDN	NIC	371.32
IDN	NOR	113.47
IDN	PAN	621.40
IDN	PRY	27.02
IDN	POL	1372.16
IDN	ROU	73.10
IDN	STP	2.40
IDN	SAU	0.18
IDN	SEN	598.68
IDN	SLE	5.26
IDN	SVK	11586.00
IDN	LKA	1322.54
IDN	KNA	169.50
IDN	SUR	83.14
IDN	SWE	2.92
IDN	THA	105.20
IDN	TLS	2935.76
IDN	TGO	54.00
IDN	TON	12.30
IDN	TTO	1.98
IDN	TKM	591.06
IDN	GBR	960.84
IDN	USA	1321.84
IDN	URY	9793.20
IDN	VEN	2.90
IDN	YEM	830.94
IDN	MKD	48.26
IRN	AFG	313.88
IRN	ARM	120.94
IRN	AZE	286.54
IRN	CHN	7570.02
IRN	FRA	2467.22
IRN	GEO	28.02
IRN	DEU	414.06
IRN	GRC	1853.98
IRN	HKG	133.12
IRN	IND	3628.30
IRN	ITA	4070.42
IRN	JPN	10869.80
IRN	KOR	3869.18
IRN	KWT	217.10
IRN	KGZ	6.46
IRN	MWI	22.00
IRN	MOZ	527.74
IRN	NZL	593.80
IRN	PAK	78.98
IRN	PAN	363.18
IRN	POL	891.64
IRN	RWA	143.72
IRN	SEN	205.40
IRN	SVK	1231.90

IRN	ESP	2304.32
IRN	LKA	128.88
IRN	KNA	325.46
IRN	TKM	3906.08
IRN	UGA	119.00
IRN	GBR	1217.86
IRN	UZB	49.38
IRL	AUS	1406.78
IRL	AUT	826.18
IRL	BEL	18547.70
IRL	KHM	2.80
IRL	CAN	1787.76
IRL	CHN	1095.28
IRL	COM	0.34
IRL	COD	8.60
IRL	CRI	256.72
IRL	CZE	487.04
IRL	DNK	881.74
IRL	FIN	628.12
IRL	FRA	8300.64
IRL	DEU	19723.60
IRL	GRC	513.42
IRL	HKG	930.62
IRL	ISL	97.02
IRL	ISR	342.18
IRL	ITA	4848.82
IRL	JPN	2613.98
IRL	KEN	136.55
IRL	KOR	653.52
IRL	LUX	107.74
IRL	MDV	808.44
IRL	MLT	11.44
IRL	MRT	23.38
IRL	MDA	823.00
IRL	NZL	4777.30
IRL	OMN	798.98
IRL	POL	698.24
IRL	PRT	457.64
IRL	QAT	515.74
IRL	SRB	46.88
IRL	SVK	867.04
IRL	ESP	618.32
IRL	LKA	4301.16
IRL	SDN	0.98
IRL	CHE	1567.72
IRL	SYR	4130.00
IRL	USA	19269.20
IRL	URY	28313.30
IRL	ZWE	9.98
ISR	ARM	89.74
ISR	AUS	410.64
ISR	BEL	3207.46
ISR	BWA	19.26
ISR	BRA	491.38
ISR	CAN	499.36
ISR	CHN	829.40
ISR	CYP	442.72
ISR	ETH	35.18
ISR	FRA	950.66
ISR	DEU	1500.24
ISR	HKG	2334.30
ISR	IND	1176.28
ISR	ITA	967.46

ISR	JPN	753.88
ISR	JOR	148.52
ISR	KOR	510.12
ISR	MRT	16.12
ISR	MNE	5.40
ISR	NZL	1299.84
ISR	RWA	418.52
ISR	WSM	3.82
ISR	SVK	367.72
ISR	ESP	282.02
ISR	LKA	768.20
ISR	SYR	806.48
ISR	TLS	451.36
ISR	TKM	840.80
ISR	USA	1572.40
ISR	URY	17080.80
ITA	ALB	827.50
ITA	DZA	1479.66
ITA	ATG	10.53
ITA	ARG	762.26
ITA	ARM	63.38
ITA	AUS	3331.66
ITA	AUT	9322.82
ITA	AZE	108.18
ITA	BHS	7.10
ITA	BHR	141.47
ITA	BGD	152.16
ITA	BRB	12.88
ITA	BLR	422.62
ITA	BEL	10954.10
ITA	BEN	17.98
ITA	BOL	23.60
ITA	BIH	600.28
ITA	BWA	5.86
ITA	BRA	2399.32
ITA	BRN	18.63
ITA	BGR	1763.66
ITA	BFA	13.58
ITA	BDI	8.36
ITA	KHM	5.50
ITA	CMR	72.28
ITA	CAN	3932.16
ITA	CPV	20.12
ITA	CAF	0.93
ITA	CHL	579.53
ITA	CHN	7453.70
ITA	COL	367.62
ITA	COM	0.68
ITA	COD	43.20
ITA	CRI	103.94
ITA	CIV	125.40
ITA	HRV	3223.18
ITA	CYP	675.54
ITA	CZE	3950.32
ITA	DNK	3090.28
ITA	ECU	136.42
ITA	EGY	797.46
ITA	SLV	50.42
ITA	ERI	60.58
ITA	EST	344.80
ITA	ETH	301.56
ITA	FJI	3.92
ITA	FIN	2159.40

ITA	FRA	46150.20
ITA	GAB	31.16
ITA	GMB	3.32
ITA	GEO	81.66
ITA	DEU	51279.40
ITA	GHA	167.34
ITA	GRC	8123.22
ITA	GRD	1.12
ITA	GTM	91.28
ITA	HKG	3798.66
ITA	HUN	3885.14
ITA	ISL	176.44
ITA	IND	2076.24
ITA	IDN	517.02
ITA	IRN	1877.40
ITA	IRL	1605.86
ITA	ISR	1767.78
ITA	JPN	6834.38
ITA	JOR	349.72
ITA	KAZ	783.42
ITA	KEN	182.30
ITA	KOR	2832.74
ITA	KGZ	7.82
ITA	LVA	340.34
ITA	LBN	736.62
ITA	LSO	1.06
ITA	LBY	931.90
ITA	LTU	582.18
ITA	LUX	470.36
ITA	MDG	202.98
ITA	MWI	32.36
ITA	MYS	7.32
ITA	MDV	1050.42
ITA	MLI	11.82
ITA	MLT	21.72
ITA	MRT	1063.68
ITA	MUS	9.22
ITA	MEX	89.02
ITA	MDA	3688.48
ITA	MNG	173.16
ITA	MNE	4.92
ITA	MOZ	1393.28
ITA	MMR	20.90
ITA	NPL	9.94
ITA	NLD	8.60
ITA	NZL	9180.26
ITA	NIC	571.08
ITA	NER	14.38
ITA	NGA	8.48
ITA	NOR	559.13
ITA	OMN	1986.40
ITA	PAK	259.68
ITA	PAN	429.16
ITA	PNG	26.40
ITA	PRY	2.42
ITA	PER	25.66
ITA	PHL	230.84
ITA	PRT	7927.86
ITA	QAT	4265.10
ITA	ROU	621.32
ITA	RUS	6597.02
ITA	RWA	8136.26
ITA	WSM	4.56

ITA	SEN	2435.08
ITA	SRB	101.80
ITA	SYC	1211.30
ITA	SLE	39.52
ITA	SVK	2317.94
ITA	SVN	1802.26
ITA	SLB	3855.00
ITA	ESP	1691.14
ITA	LKA	28158.80
ITA	KNA	111.68
ITA	LCA	0.74
ITA	VCT	3.24
ITA	SDN	2.60
ITA	SUR	198.62
ITA	SWZ	3.96
ITA	SWE	4.80
ITA	CHE	4064.00
ITA	SYR	14939.70
ITA	TJK	336.38
ITA	THA	59.50
ITA	TLS	1164.30
ITA	TGO	1.10
ITA	TON	22.45
ITA	TUN	110.66
ITA	TUR	2796.14
ITA	TKM	7693.78
ITA	UKR	34.14
ITA	ARE	1160.80
ITA	GBR	3632.64
ITA	USA	24782.80
ITA	URY	31812.50
ITA	UZB	73.60
ITA	VEN	0.55
ITA	VNM	496.24
ITA	YEM	316.76
ITA	MKD	64.08
ITA	ZWE	10.18
JAM	ATG	4.53
JAM	BHS	1.45
JAM	BRB	12.02
JAM	BLZ	4.00
JAM	CAN	280.50
JAM	CHN	148.82
JAM	DOM	6.36
JAM	FIN	4.22
JAM	FRA	60.20
JAM	GEO	11.70
JAM	DEU	73.10
JAM	GRD	1.64
JAM	GUY	3.88
JAM	ISL	18.00
JAM	JPN	26.46
JAM	MDA	4.50
JAM	NZL	110.80
JAM	OMN	67.40
JAM	RUS	5.78
JAM	RWA	28.14
JAM	SVN	9.52
JAM	LCA	1.80
JAM	VCT	4.00
JAM	SDN	1.52
JAM	SWZ	3.36
JAM	CHE	13.44

JAM	SYR	4.30
JAM	TUN	18.06
JAM	GBR	5.34
JAM	USA	177.12
JAM	URY	492.90
JPN	AFG	531.80
JPN	DZA	582.16
JPN	ATG	19.30
JPN	ARG	739.48
JPN	AUS	12772.10
JPN	AUT	2368.48
JPN	AZE	156.52
JPN	BHS	34.15
JPN	BHR	473.97
JPN	BGD	775.84
JPN	BRB	72.06
JPN	BLR	98.14
JPN	BEL	8735.88
JPN	BLZ	9.38
JPN	BEN	20.68
JPN	BOL	176.90
JPN	BWA	19.68
JPN	BRA	3448.66
JPN	BRN	181.57
JPN	BGR	194.70
JPN	BFA	64.68
JPN	BDI	27.56
JPN	KHM	69.26
JPN	CMR	99.06
JPN	CAN	12093.90
JPN	CPV	19.66
JPN	CAF	6.10
JPN	CHL	1136.08
JPN	CHN	103700.00
JPN	COL	843.72
JPN	COD	23.30
JPN	CRI	528.30
JPN	CIV	103.40
JPN	HRV	308.04
JPN	CYP	224.44
JPN	CZE	2504.56
JPN	DNK	669.24
JPN	DOM	6.84
JPN	ECU	392.56
JPN	EGY	429.00
JPN	SLV	145.42
JPN	ERI	5.73
JPN	EST	343.78
JPN	ETH	246.56
JPN	FJI	60.50
JPN	FIN	1940.92
JPN	FRA	12939.00
JPN	GAB	34.12
JPN	GMB	5.86
JPN	GEO	22.40
JPN	DEU	27613.90
JPN	GHA	130.90
JPN	GRC	1587.52
JPN	GRD	10.96
JPN	GTM	324.44
JPN	GUY	31.08
JPN	HND	121.64
JPN	HKG	35326.40

JPN	HUN	2186.80
JPN	ISL	216.04
JPN	IND	4049.60
JPN	IDN	8382.28
JPN	IRN	954.53
JPN	IRL	2466.56
JPN	ISR	1290.56
JPN	ITA	6640.40
JPN	JAM	199.98
JPN	JOR	305.98
JPN	KAZ	698.52
JPN	KEN	509.65
JPN	KIR	12.60
JPN	KOR	47807.40
JPN	KGZ	13.26
JPN	LBN	256.36
JPN	LSO	2.06
JPN	LBY	340.07
JPN	LTU	95.16
JPN	LUX	155.02
JPN	MWI	46.50
JPN	MYS	22.08
JPN	MDV	16833.50
JPN	MLI	17.88
JPN	MLT	41.02
JPN	MRT	81.50
JPN	MUS	24.08
JPN	MEX	110.12
JPN	MDA	12578.70
JPN	MNG	23.06
JPN	MNE	83.78
JPN	MOZ	389.48
JPN	MMR	35.96
JPN	NPL	6.94
JPN	NLD	34.17
JPN	NZL	14314.90
JPN	NIC	2572.14
JPN	NER	105.50
JPN	NGA	32.10
JPN	NOR	517.93
JPN	OMN	1666.76
JPN	PAK	1524.12
JPN	PAN	1435.42
JPN	PNG	7127.60
JPN	PRY	87.58
JPN	PER	343.34
JPN	PHL	505.90
JPN	POL	9232.40
JPN	PRT	2039.80
JPN	QAT	750.38
JPN	ROU	879.44
JPN	RUS	481.16
JPN	RWA	6432.04
JPN	WSM	12.20
JPN	STP	12.42
JPN	SAU	2.28
JPN	SEN	5416.38
JPN	SRB	75.92
JPN	SLE	8.68
JPN	SVK	19039.90
JPN	SVN	698.54
JPN	SLB	129.26
JPN	ESP	3822.86

JPN	LKA	7161.08
JPN	KNA	386.42
JPN	LCA	8.24
JPN	VCT	25.04
JPN	SDN	8.34
JPN	SUR	476.24
JPN	SWZ	55.62
JPN	SWE	10.78
JPN	CHE	2277.56
JPN	SYR	2463.38
JPN	TJK	217.22
JPN	THA	216.86
JPN	TLS	24513.40
JPN	TGO	5.90
JPN	TON	10.45
JPN	TTO	3.26
JPN	TUN	211.76
JPN	TUR	241.32
JPN	TKM	2924.18
JPN	UKR	153.30
JPN	ARE	720.90
JPN	GBR	5437.36
JPN	USA	15001.10
JPN	URY	139638.00
JPN	UZB	45.02
JPN	VEN	6.30
JPN	VNM	631.98
JPN	YEM	3563.12
JPN	MKD	143.84
JPN	ZMB	38.58
JPN	ZWE	25.58
JOR	DZA	74.82
JOR	BHR	32.54
JOR	CHN	45.30
JOR	EGY	47.46
JOR	ETH	30.58
JOR	IND	333.42
JOR	IDN	27.22
JOR	IRN	42.08
JOR	ISR	125.78
JOR	JPN	33.34
JOR	KWT	68.30
JOR	LBN	72.26
JOR	LBY	26.38
JOR	MDV	32.36
JOR	ROU	41.30
JOR	SEN	277.90
JOR	SUR	91.30
JOR	SYR	114.84
JOR	TJK	205.70
JOR	GBR	218.10
JOR	URY	1069.68
JOR	MKD	32.94
KAZ	AFG	22.10
KAZ	ARM	48.88
KAZ	AZE	214.98
KAZ	CAN	296.12
KAZ	CHN	3048.12
KAZ	FIN	232.14
KAZ	FRA	2347.96
KAZ	GEO	26.40
KAZ	DEU	341.18
KAZ	GRC	465.44

KAZ	IRN	1307.64
KAZ	ISR	545.14
KAZ	ITA	4595.68
KAZ	KGZ	243.18
KAZ	MNG	36.50
KAZ	MNE	31.90
KAZ	NZL	1139.48
KAZ	PRT	243.72
KAZ	QAT	276.40
KAZ	RUS	1025.74
KAZ	RWA	3511.68
KAZ	LKA	501.54
KAZ	SYR	5029.38
KAZ	TKM	337.18
KAZ	ARE	745.26
KAZ	USA	592.78
KAZ	URY	382.10
KAZ	VUT	367.72
KEN	AFG	43.92
KEN	BEL	34.48
KEN	BDI	39.12
KEN	COM	1.48
KEN	COD	73.00
KEN	EGY	110.36
KEN	ERI	10.65
KEN	ETH	39.50
KEN	FRA	52.54
KEN	DEU	72.30
KEN	IND	55.74
KEN	ITA	30.40
KEN	MYS	21.18
KEN	NZL	251.78
KEN	PAN	172.84
KEN	WSM	87.92
KEN	SLE	4.76
KEN	SUR	103.24
KEN	SYR	31.42
KEN	THA	252.62
KEN	UKR	464.72
KEN	GBR	62.26
KEN	USA	343.52
KEN	URY	178.86
KEN	MKD	28.28
KEN	ZMB	46.06
KIR	AUS	0.80
KIR	BGD	0.10
KIR	FJI	0.60
KIR	HKG	0.30
KOR	AFG	76.58
KOR	ALB	16.16
KOR	DZA	328.58
KOR	ATG	3.40
KOR	ARG	372.50
KOR	ARM	17.28
KOR	AUS	4163.20
KOR	AUT	888.12
KOR	AZE	44.04
KOR	BHR	109.70
KOR	BGD	534.46
KOR	BRB	17.04
KOR	BEL	1969.98
KOR	BLZ	3.02
KOR	BEN	5.48

KOR	BOL	18.32
KOR	BWA	4.94
KOR	BRA	2326.58
KOR	BRN	28.90
KOR	KHM	86.48
KOR	CAN	4535.46
KOR	CHL	1623.78
KOR	CHN	75131.70
KOR	COL	619.18
KOR	CRI	172.40
KOR	CIV	58.82
KOR	HRV	194.02
KOR	CYP	44.42
KOR	CZE	659.20
KOR	DNK	739.84
KOR	ECU	361.40
KOR	EGY	280.68
KOR	SLV	87.05
KOR	ERI	4.03
KOR	EST	88.06
KOR	ETH	70.64
KOR	FJI	14.94
KOR	FIN	872.20
KOR	FRA	4307.30
KOR	DEU	10934.20
KOR	GHA	120.80
KOR	GRC	2268.84
KOR	GRD	1.52
KOR	GTM	284.62
KOR	HND	30.46
KOR	HKG	17188.90
KOR	HUN	1322.76
KOR	ISL	46.20
KOR	IND	4643.12
KOR	IDN	4548.76
KOR	IRN	1498.60
KOR	IRL	1100.12
KOR	ISR	804.80
KOR	ITA	4161.48
KOR	JPN	23799.90
KOR	JOR	311.34
KOR	KAZ	320.72
KOR	KEN	85.80
KOR	KIR	0.10
KOR	KGZ	26.54
KOR	LBN	86.82
KOR	LSO	4.40
KOR	LBY	291.87
KOR	MDG	33.30
KOR	MWI	20.86
KOR	MYS	7.16
KOR	MDV	5951.64
KOR	MLI	2.70
KOR	MRT	39.28
KOR	MDA	7814.20
KOR	MNG	20.30
KOR	MNE	78.66
KOR	MOZ	285.40
KOR	NPL	3.62
KOR	NLD	36.70
KOR	NZL	3457.40
KOR	NIC	676.68
KOR	NER	33.30

KOR	NOR	442.27
KOR	OMN	631.16
KOR	PAK	554.00
KOR	PAN	536.52
KOR	PNG	150.03
KOR	PRY	8.54
KOR	PER	50.20
KOR	PHL	367.02
KOR	POL	3584.94
KOR	PRT	1996.86
KOR	ROU	371.22
KOR	RUS	547.12
KOR	RWA	4596.20
KOR	STP	1.68
KOR	SEN	2442.88
KOR	SVK	8769.60
KOR	SVN	1245.26
KOR	SLB	209.18
KOR	ESP	1311.60
KOR	LKA	3871.84
KOR	KNA	275.74
KOR	VCT	3.06
KOR	SUR	190.22
KOR	SWE	4.38
KOR	CHE	957.88
KOR	SYR	552.78
KOR	TJK	326.96
KOR	THA	46.54
KOR	TLS	4203.54
KOR	TGO	0.45
KOR	TTO	0.22
KOR	TUN	45.36
KOR	TUR	98.68
KOR	TKM	3045.60
KOR	UKR	18.10
KOR	ARE	740.78
KOR	GBR	2825.60
KOR	USA	5490.74
KOR	URY	45727.40
KOR	UZB	46.90
KOR	VEN	2.50
KOR	VNM	353.58
KOR	YEM	3787.22
KOR	MKD	57.32
KOR	ZMB	11.84
KOR	ZWE	10.98
KWT	BHR	37.40
KWT	BGD	572.42
KWT	EGY	645.92
KWT	IND	2674.62
KWT	IDN	1248.12
KWT	JPN	7394.68
KWT	KOR	5976.08
KWT	NLD	15.27
KWT	PAN	1365.56
KWT	SVK	3709.04
KWT	YEM	216.74
KWT	MKD	306.38
KWT	ZWE	98.13
KGZ	AFG	43.94
KGZ	AZE	2.06
KGZ	BLR	1.52
KGZ	BEL	5.70

KGZ	BGR	3.18
KGZ	CAN	19.44
KGZ	CHN	37.84
KGZ	DEU	3.76
KGZ	IND	3.20
KGZ	IRN	5.56
KGZ	ITA	2.92
KGZ	KAZ	125.54
KGZ	LVA	8.22
KGZ	NZL	3.28
KGZ	RUS	1.58
KGZ	RWA	151.50
KGZ	SYR	143.76
KGZ	TZA	23.16
KGZ	TKM	23.28
KGZ	UGA	2.62
KGZ	ARE	5.16
KGZ	GBR	106.00
KGZ	USA	3.38
KGZ	URY	4.62
KGZ	VUT	32.34
LVA	AUT	125.30
LVA	BLR	562.90
LVA	BEL	267.80
LVA	CZE	138.00
LVA	DNK	1290.90
LVA	EST	2964.10
LVA	FIN	785.60
LVA	FRA	466.20
LVA	DEU	2772.60
LVA	ISL	196.40
LVA	IRL	352.70
LVA	ITA	483.00
LVA	JPN	204.40
LVA	KAZ	126.60
LVA	LTU	3287.40
LVA	NZL	615.50
LVA	OMN	621.30
LVA	PRT	885.80
LVA	RWA	2157.70
LVA	LKA	292.60
LVA	CHE	2124.20
LVA	SYR	284.80
LVA	ARE	435.60
LVA	USA	2497.50
LVA	URY	648.00
LBN	DZA	15.54
LBN	BEL	14.82
LBN	CAN	9.94
LBN	CAF	0.60
LBN	COD	2.30
LBN	CYP	11.30
LBN	EGY	28.46
LBN	FRA	31.24
LBN	DEU	18.38
LBN	GRC	9.22
LBN	IND	16.70
LBN	ITA	22.80
LBN	JOR	41.68
LBN	KWT	40.92
LBN	MRT	14.22
LBN	NZL	13.96
LBN	NOR	12.70

LBN	ROU	15.60
LBN	SAU	0.02
LBN	SEN	95.24
LBN	LKA	16.28
LBN	SYR	162.26
LBN	TJK	125.66
LBN	TKM	54.14
LBN	GBR	96.40
LBN	USA	16.66
LBN	URY	55.50
LSO	BEL	24.76
LSO	BWA	0.94
LSO	CAN	10.50
LSO	CHN	0.02
LSO	FRA	0.56
LSO	GAB	0.12
LSO	DEU	0.02
LSO	GRC	0.24
LSO	HKG	0.06
LSO	IRL	0.02
LSO	ITA	0.56
LSO	JPN	0.06
LSO	KEN	0.22
LSO	MWI	0.16
LSO	MDA	0.04
LSO	NZL	0.18
LSO	NOR	0.14
LSO	SVK	0.04
LSO	ESP	131.02
LSO	SWE	3.32
LSO	SYR	5.42
LSO	USA	0.18
LSO	URY	305.84
LSO	ZWE	0.10
LBR	BGD	215.20
LBY	AUT	18.90
LBY	BEL	12.80
LBY	BRA	4.90
LBY	CYP	4.10
LBY	EGY	57.10
LBY	FRA	574.40
LBY	DEU	1556.20
LBY	GRC	780.54
LBY	IND	28.80
LBY	ITA	10970.00
LBY	LBN	16.20
LBY	MDV	16.60
LBY	MRT	76.50
LBY	MDA	13.80
LBY	MOZ	41.80
LBY	NZL	70.00
LBY	QAT	577.94
LBY	LKA	2972.78
LBY	CHE	15.10
LBY	SYR	1001.94
LBY	TJK	4.90
LBY	TUR	532.74
LBY	TKM	1454.44
LBY	USA	233.60
LTU	BLR	431.28
LTU	BEL	221.32
LTU	CAN	161.58
LTU	CZE	81.78

LTU	DNK	516.14
LTU	EST	675.42
LTU	FIN	131.68
LTU	FRA	598.46
LTU	DEU	1156.58
LTU	ISL	63.96
LTU	ITA	253.02
LTU	KAZ	160.42
LTU	LVA	1325.26
LTU	NZL	452.36
LTU	OMN	247.30
LTU	PRT	653.72
LTU	RWA	1470.32
LTU	SVK	149.46
LTU	LKA	212.28
LTU	CHE	679.12
LTU	SYR	295.00
LTU	TKM	137.06
LTU	ARE	300.40
LTU	USA	581.20
LTU	URY	449.54
LUX	ARM	19.02
LUX	AUT	291.86
LUX	BEL	2210.40
LUX	KHM	3.44
LUX	CAN	63.62
LUX	CHN	188.52
LUX	CZE	114.44
LUX	DNK	115.08
LUX	FIN	76.52
LUX	FRA	2331.96
LUX	DEU	3310.72
LUX	HKG	47.88
LUX	HUN	64.38
LUX	ITA	767.90
LUX	NZL	636.28
LUX	OMN	58.86
LUX	PRT	185.84
LUX	QAT	69.58
LUX	RWA	108.80
LUX	LKA	440.60
LUX	CHE	177.68
LUX	SYR	167.82
LUX	TKM	98.02
LUX	GBR	48.98
LUX	USA	650.64
LUX	URY	355.80
MDG	ALB	38.30
MDG	AUT	12.02
MDG	BEL	64.06
MDG	BIH	54.56
MDG	BGR	142.46
MDG	HRV	147.48
MDG	CZE	7.24
MDG	FRA	35.62
MDG	DEU	363.50
MDG	GRC	300.58
MDG	ITA	196.62
MDG	NZL	53.18
MDG	QAT	7.52
MDG	RUS	7.86
MDG	RWA	20.78
MDG	SYC	598.65

MDG	SLB	38.04
MDG	LKA	56.34
MDG	CHE	10.76
MDG	SYR	10.96
MDG	TKM	48.30
MDG	USA	46.36
MDG	URY	52.60
MWI	BHR	5.68
MWI	BEL	12.34
MWI	CAN	10.40
MWI	CHN	20.12
MWI	COM	4.42
MWI	FRA	407.04
MWI	DEU	56.90
MWI	HKG	10.10
MWI	IND	10.56
MWI	ITA	30.84
MWI	JPN	13.76
MWI	MEX	29.34
MWI	MOZ	5.78
MWI	NZL	15.74
MWI	QAT	2.08
MWI	SVK	20.58
MWI	ESP	8.72
MWI	LKA	17.98
MWI	CHE	4.98
MWI	SYR	5.68
MWI	TLS	8.80
MWI	GBR	2.14
MWI	USA	24.90
MWI	URY	227.86
MYS	AUS	4.74
MYS	BEL	27.24
MYS	DNK	8.66
MYS	EGY	22.74
MYS	FRA	7.96
MYS	DEU	46.04
MYS	GRC	4.62
MYS	IND	4.40
MYS	JPN	9.54
MYS	KEN	17.50
MYS	KOR	8.06
MYS	MMR	19.90
MYS	NZL	25.94
MYS	PRT	9.72
MYS	QAT	8.48
MYS	RWA	17.14
MYS	ESP	102.22
MYS	LKA	9.76
MYS	SYR	16.06
MYS	THA	4.62
MYS	TKM	7.34
MYS	USA	57.62
MYS	URY	53.18
MYS	ZMB	11.48
MYS	ZWE	50.33
MDV	ARG	170.72
MDV	AUS	4529.40
MDV	AZE	53.76
MDV	BHR	56.10
MDV	BGD	419.44
MDV	BEL	604.94
MDV	BEN	6.30

MDV	BRA	755.56
MDV	BRN	279.60
MDV	KHM	67.32
MDV	CAN	2226.26
MDV	CHN	20904.80
MDV	CZE	636.02
MDV	EGY	213.70
MDV	ETH	40.72
MDV	FJI	22.40
MDV	FRA	1970.28
MDV	GMB	1.40
MDV	DEU	3184.68
MDV	GHA	37.18
MDV	GUY	3.52
MDV	HKG	7734.48
MDV	HUN	519.36
MDV	IND	4111.54
MDV	IDN	3548.12
MDV	IRN	255.13
MDV	IRL	601.26
MDV	ITA	836.98
MDV	JPN	14858.60
MDV	KIR	0.20
MDV	KOR	6324.82
MDV	LSO	3.44
MDV	MWI	20.58
MDV	MLI	58.52
MDV	MRT	70.50
MDV	MEX	88.22
MDV	MDA	3807.56
MDV	MNE	9.02
MDV	NLD	40.63
MDV	NZL	5108.32
MDV	NIC	642.46
MDV	NGA	7.52
MDV	PAK	86.68
MDV	PAN	843.12
MDV	PRY	28.74
MDV	PER	34.92
MDV	PHL	100.06
MDV	POL	2017.40
MDV	ROU	102.48
MDV	STP	1.88
MDV	SEN	562.46
MDV	SLE	10.14
MDV	SVK	27891.40
MDV	SVN	170.56
MDV	ESP	719.38
MDV	LKA	675.16
MDV	KNA	265.88
MDV	SUR	72.78
MDV	TJK	114.26
MDV	THA	60.32
MDV	TLS	7091.10
MDV	TGO	0.75
MDV	TTO	0.36
MDV	UKR	53.76
MDV	GBR	1946.32
MDV	USA	2681.22
MDV	URY	32227.40
MDV	VEN	1.00
MDV	YEM	1442.24
MDV	MKD	96.62

MLI	CAN	0.14
MLI	CHN	0.16
MLI	DNK	0.10
MLI	FRA	4.48
MLI	DEU	4.56
MLI	GRC	0.16
MLI	HKG	1.06
MLI	IND	1.04
MLI	IDN	1.18
MLI	IRN	1.10
MLI	ITA	3.00
MLI	JPN	14.80
MLI	KOR	1.58
MLI	MDV	0.48
MLI	NZL	1.90
MLI	PAK	0.38
MLI	PNG	0.20
MLI	SLE	0.40
MLI	SVK	5.96
MLI	KNA	17.02
MLI	TLS	30.22
MLI	GBR	15.44
MLI	USA	12.72
MLI	URY	15.30
MLT	BGD	14.02
MLT	BEL	13.76
MLT	BFA	7.60
MLT	CHN	49.62
MLT	CIV	27.62
MLT	FRA	22.74
MLT	DEU	13.26
MLT	GHA	7.76
MLT	GIN	8.42
MLT	IND	6.24
MLT	IDN	15.50
MLT	ITA	11.76
MLT	MDV	8.22
MLT	MUS	4.36
MLT	MOZ	4.52
MLT	NGA	4.34
MLT	PAN	15.80
MLT	SRB	53.18
MLT	SVK	8.12
MLT	ESP	549.48
MLT	SYR	240.48
MLT	TLS	27.20
MLT	USA	14.72
MLT	YEM	21.96
MRT	BEL	40.90
MRT	BRA	10.28
MRT	CAN	12.48
MRT	CPV	1.94
MRT	CHN	31.36
MRT	CZE	12.00
MRT	FIN	30.32
MRT	FRA	373.54
MRT	DEU	320.38
MRT	HKG	101.06
MRT	HUN	29.52
MRT	ITA	102.74
MRT	JPN	119.36
MRT	KOR	20.20
MRT	LBY	92.57

MRT	MDV	14.92
MRT	NZL	20.74
MRT	SEN	10.46
MRT	SVK	369.44
MRT	LKA	18.34
MRT	CHE	9.80
MRT	TUR	10.50
MRT	GBR	13.46
MRT	URY	356.26
MUS	DZA	10.05
MUS	BEL	56.03
MUS	CMR	29.44
MUS	CHN	6.78
MUS	CIV	60.60
MUS	FIN	0.65
MUS	FRA	70.88
MUS	DEU	50.05
MUS	GHA	2.35
MUS	GRC	1.28
MUS	HKG	0.43
MUS	IND	0.60
MUS	ITA	61.63
MUS	JPN	59.23
MUS	NOR	9.00
MUS	PAN	1.48
MUS	RWA	6.23
MUS	SRB	0.75
MUS	LKA	52.80
MUS	CHE	3.70
MUS	SYR	0.43
MUS	TLS	0.83
MUS	TON	0.33
MUS	USA	4.53
MEX	AUT	4.78
MEX	BEL	52.24
MEX	CHN	5.92
MEX	COM	2.92
MEX	DNK	5.44
MEX	FRA	359.00
MEX	DEU	46.58
MEX	HKG	7.08
MEX	IND	11.16
MEX	ITA	92.28
MEX	JPN	15.22
MEX	KEN	7.20
MEX	MWI	111.30
MEX	MDV	5.58
MEX	NZL	30.18
MEX	QAT	19.44
MEX	SLE	18.26
MEX	SVK	7.64
MEX	ESP	38.86
MEX	LKA	50.98
MEX	SYR	22.32
MEX	GBR	106.14
MEX	USA	650.32
MEX	URY	227.70
MDA	ARG	836.46
MDA	AUS	340.78
MDA	BHS	8.55
MDA	BRB	14.08
MDA	BEL	520.10
MDA	BLZ	52.88

MDA	BOL	46.66
MDA	BRA	1073.88
MDA	CAN	12255.30
MDA	CHL	930.53
MDA	CHN	1233.34
MDA	COL	1760.04
MDA	CRI	508.46
MDA	DOM	556.20
MDA	ECU	325.98
MDA	SLV	529.68
MDA	FRA	476.20
MDA	DEU	2599.76
MDA	GRD	1.62
MDA	GTM	828.64
MDA	GUY	3.88
MDA	HND	260.42
MDA	IND	643.00
MDA	JAM	102.94
MDA	JPN	1338.66
MDA	NZL	1056.92
MDA	NER	366.52
MDA	PNG	486.98
MDA	PER	54.30
MDA	PHL	440.26
MDA	LKA	2960.28
MDA	SDN	1.08
MDA	SWZ	4.90
MDA	TUN	56.08
MDA	USA	1036.46
MDA	URY	186117.00
MDA	UZB	49.06
MDA	VNM	1287.02
MNG	AUT	15.40
MNG	AZE	3.88
MNG	BLR	70.38
MNG	BEL	11.78
MNG	BGR	12.14
MNG	CZE	3.86
MNG	FRA	17.18
MNG	DEU	62.62
MNG	GRC	5.48
MNG	HUN	11.38
MNG	ITA	121.88
MNG	KAZ	22.32
MNG	LTU	6.56
MNG	NZL	7.72
MNG	PRT	24.78
MNG	RUS	133.52
MNG	RWA	284.80
MNG	SVN	9.92
MNG	LKA	4.02
MNG	SYR	9.46
MNG	TJK	3.62
MNG	TKM	20.96
MNG	ARE	103.52
MNG	USA	17.62
MNG	URY	29.04
MNE	AUS	10.02
MNE	BEL	0.72
MNE	CAN	97.44
MNE	CHN	729.94
MNE	EST	0.78
MNE	FRA	5.04

MNE	DEU	9.64
MNE	HKG	4.08
MNE	IND	0.76
MNE	ITA	29.48
MNE	JPN	11.12
MNE	KAZ	1.18
MNE	KOR	28.86
MNE	KWT	0.88
MNE	KGZ	0.56
MNE	LUX	3.22
MNE	NZL	4.02
MNE	RWA	38.26
MNE	SEN	0.64
MNE	SVK	11.64
MNE	SYR	3.00
MNE	ARE	5.04
MNE	USA	61.28
MNE	URY	127.04
MNE	VUT	3.80
MOZ	DZA	49.02
MOZ	BEL	293.72
MOZ	BRA	289.06
MOZ	CAN	68.56
MOZ	CHN	82.20
MOZ	COM	0.64
MOZ	CIV	28.22
MOZ	FRA	3470.64
MOZ	GAB	6.62
MOZ	GMB	1.18
MOZ	DEU	349.30
MOZ	GRC	75.68
MOZ	IND	430.30
MOZ	ITA	567.20
MOZ	JPN	123.38
MOZ	LBY	42.47
MOZ	MUS	7.68
MOZ	MDA	54.58
MOZ	NZL	289.76
MOZ	NIC	67.64
MOZ	PAN	88.02
MOZ	QAT	173.10
MOZ	RWA	127.66
MOZ	SEN	64.92
MOZ	SRB	42.54
MOZ	LKA	2793.90
MOZ	SYR	124.54
MOZ	TUR	65.10
MOZ	TKM	91.18
MOZ	USA	722.42
MOZ	URY	301.52
MMR	AGO	1.05
MMR	BEL	5.60
MMR	CHN	27.15
MMR	FRA	6.50
MMR	DEU	13.35
MMR	IND	31.60
MMR	IDN	8.90
MMR	JPN	9.90
MMR	KEN	2.00
MMR	MYS	126.80
MMR	MDV	6.45
MMR	NZL	1169.35
MMR	QAT	38.35

MMR	RWA	4.65
MMR	ESP	273.60
MMR	LKA	40.70
MMR	SWE	9.68
MMR	SYR	27.05
MMR	TLS	3.00
MMR	TKM	4.75
MMR	URY	9.45
MMR	ZMB	18.48
MMR	ZWE	156.18
NAM	TLS	1736.62
NPL	AGO	238.98
NPL	BEL	9.76
NPL	BLZ	14.34
NPL	BWA	16.68
NPL	CAN	94.78
NPL	CAF	0.50
NPL	CHN	50.98
NPL	COD	22.06
NPL	FRA	89.56
NPL	DEU	70.46
NPL	ISR	36.82
NPL	ITA	188.16
NPL	JPN	22.02
NPL	KOR	35.22
NPL	MDV	16.88
NPL	MMR	16.20
NPL	NZL	36.28
NPL	ESP	760.96
NPL	LKA	191.44
NPL	SWE	7.60
NPL	SYR	14.48
NPL	GBR	16.28
NPL	USA	545.42
NPL	URY	128.74
NPL	YEM	11.48
NLD	AUS	1.30
NLD	AUT	2.63
NLD	BGD	8.70
NLD	BEL	6.17
NLD	BTN	0.80
NLD	CAN	4.73
NLD	CHN	10.40
NLD	DNK	0.60
NLD	FRA	7.17
NLD	DEU	81.07
NLD	HKG	2.97
NLD	IND	282.07
NLD	ITA	5.57
NLD	JPN	6.37
NLD	NZL	2.40
NLD	OMN	0.57
NLD	QAT	2.43
NLD	SVK	1.40
NLD	LKA	2.67
NLD	CHE	0.93
NLD	SYR	5.13
NLD	TLS	0.60
NLD	TKM	0.57
NLD	USA	11.97
NLD	URY	173.93
NZL	ALB	18.68
NZL	DZA	236.18

NZL	ATG	2.43
NZL	ARG	220.86
NZL	AUS	989.54
NZL	AUT	4695.66
NZL	AZE	109.74
NZL	BHS	4.20
NZL	BHR	73.43
NZL	BRB	12.64
NZL	BLR	168.74
NZL	BEL	55986.30
NZL	BLZ	9.14
NZL	BEN	26.64
NZL	BOL	6.78
NZL	BIH	69.84
NZL	BWA	6.58
NZL	BRN	9.53
NZL	BGR	353.54
NZL	BFA	16.36
NZL	BDI	3.90
NZL	CMR	52.12
NZL	CAN	1343.72
NZL	CPV	47.26
NZL	CAF	3.18
NZL	CHL	227.18
NZL	CHN	3325.08
NZL	COL	199.26
NZL	COM	0.72
NZL	COD	256.50
NZL	CRI	142.56
NZL	CIV	121.38
NZL	HRV	349.78
NZL	CYP	239.22
NZL	CZE	3976.96
NZL	DNK	5064.50
NZL	DOM	2.18
NZL	ECU	149.10
NZL	EGY	272.56
NZL	ERI	12.98
NZL	EST	289.62
NZL	ETH	70.78
NZL	FIN	3694.30
NZL	FRA	29848.00
NZL	GAB	41.36
NZL	GMB	9.30
NZL	GEO	57.72
NZL	DEU	79221.60
NZL	GHA	202.48
NZL	GRC	3082.08
NZL	GRD	3.94
NZL	GUY	13.54
NZL	HKG	1614.14
NZL	HUN	2866.66
NZL	ISL	258.42
NZL	IDN	446.54
NZL	IRN	499.13
NZL	IRL	3406.08
NZL	ISR	1636.16
NZL	ITA	21286.90
NZL	JAM	24.72
NZL	JPN	2768.46
NZL	JOR	119.18
NZL	KAZ	202.52
NZL	KEN	132.30

NZL	KOR	2528.16
NZL	KGZ	22.20
NZL	LVA	312.32
NZL	LBN	160.32
NZL	LSO	0.44
NZL	LBY	76.30
NZL	LTU	617.22
NZL	LUX	1032.82
NZL	MDG	58.58
NZL	MYS	13.44
NZL	MDV	845.96
NZL	MLI	8.96
NZL	MLT	17.72
NZL	MRT	126.96
NZL	MUS	11.68
NZL	MDA	1238.58
NZL	MNG	24.48
NZL	MOZ	505.12
NZL	MMR	186.84
NZL	NPL	8.46
NZL	NIC	239.60
NZL	NGA	14.68
NZL	NOR	434.97
NZL	OMN	3014.46
NZL	PAK	195.28
NZL	PNG	28.63
NZL	POL	413.14
NZL	PRT	5008.02
NZL	QAT	2761.26
NZL	ROU	132.22
NZL	RUS	1050.56
NZL	RWA	5516.40
NZL	WSM	7.56
NZL	SAU	0.30
NZL	SEN	956.02
NZL	SRB	140.10
NZL	SYC	180.43
NZL	SLE	6.50
NZL	SVK	1906.86
NZL	SVN	530.66
NZL	SLB	707.66
NZL	ESP	905.10
NZL	LKA	12608.50
NZL	KNA	59.68
NZL	VCT	6.34
NZL	SDN	1.26
NZL	SUR	112.06
NZL	SWZ	190.68
NZL	SWE	1.74
NZL	CHE	7464.80
NZL	SYR	6116.96
NZL	TJK	80.70
NZL	THA	63.24
NZL	TGO	0.05
NZL	TON	49.45
NZL	TUN	60.62
NZL	TUR	239.94
NZL	TKM	3584.76
NZL	UKR	42.42
NZL	ARE	520.24
NZL	GBR	710.16
NZL	USA	35405.30
NZL	URY	17412.40

NZL	UZB	20.18
NZL	VNM	177.88
NZL	YEM	258.30
NZL	MKD	60.76
NZL	ZMB	34.68
NZL	ZWE	17.98
NIC	ATG	2.60
NIC	AUS	4588.62
NIC	BRB	14.26
NIC	BEL	375.54
NIC	BRN	4.10
NIC	CAN	357.48
NIC	CHN	1142.90
NIC	FJI	270.10
NIC	FRA	271.98
NIC	DEU	519.76
NIC	GRD	1.78
NIC	GUY	7.06
NIC	HKG	371.92
NIC	IND	175.12
NIC	IDN	358.52
NIC	ITA	307.42
NIC	JPN	2235.84
NIC	KIR	4.00
NIC	KOR	789.16
NIC	MDV	359.00
NIC	MLI	8.92
NIC	MDA	305.08
NIC	NLD	25.40
NIC	NZL	230.74
NIC	PRY	47.82
NIC	POL	358.92
NIC	STP	56.92
NIC	SEN	259.34
NIC	SLE	2.42
NIC	SVK	315.38
NIC	LKA	151.70
NIC	KNA	80.04
NIC	TLS	274.90
NIC	TTO	41.70
NIC	USA	1014.62
NIC	URY	2895.04
NIC	VEN	29.30
NIC	YEM	143.84
NER	BEL	15.74
NER	CAN	40.88
NER	CHN	3.76
NER	CRI	53.02
NER	DOM	6.28
NER	SLV	108.32
NER	FIN	7.20
NER	FRA	9.02
NER	DEU	18.66
NER	GTM	36.24
NER	HTI	4.40
NER	HND	63.24
NER	ITA	9.64
NER	JPN	8.80
NER	MDA	43.52
NER	NZL	6.38
NER	PNG	6.48
NER	RWA	9.32
NER	LKA	29.90

NER	CHE	3.06
NER	SYR	2.40
NER	USA	19.12
NER	URY	383.48
NER	VNM	2.84
NGA	DZA	0.44
NGA	BEL	3.02
NGA	BEN	4.58
NGA	BRA	1.08
NGA	BFA	1.92
NGA	CMR	0.72
NGA	CHN	1.98
NGA	CIV	8.88
NGA	FRA	119.84
NGA	DEU	0.76
NGA	GHA	12.74
NGA	IND	0.54
NGA	JPN	43.48
NGA	LBY	0.86
NGA	MLT	0.72
NGA	NZL	1.90
NGA	NOR	52.88
NGA	ESP	1.24
NGA	LKA	10.00
NGA	SYR	35.44
NGA	TLS	1.26
NGA	TON	3.22
NGA	USA	1.92
NGA	URY	13.62
NOR	BEL	166.07
NOR	BEN	31.52
NOR	BRA	3364.52
NOR	BFA	9.28
NOR	CMR	498.17
NOR	CAN	1089.87
NOR	CHL	342.05
NOR	CIV	1223.36
NOR	FIN	218.47
NOR	FRA	1903.17
NOR	DEU	291.53
NOR	GHA	799.37
NOR	IND	3353.93
NOR	IDN	700.72
NOR	ITA	959.77
NOR	JPN	876.30
NOR	KOR	747.93
NOR	NZL	784.17
NOR	NGA	45.52
NOR	PHL	239.83
NOR	QAT	1031.00
NOR	SAU	0.18
NOR	SRB	301.14
NOR	ESP	1001.00
NOR	LKA	3682.18
NOR	TON	126.57
NOR	TUN	235.26
NOR	URY	23173.90
NOR	UZB	81.62
OMN	AZE	33.10
OMN	BHS	2.75
OMN	BEL	3817.18
OMN	BRA	338.50
OMN	CAN	4343.50

OMN	CHN	1153.74
OMN	CZE	657.70
OMN	DNK	3549.06
OMN	EST	82.10
OMN	FIN	1483.90
OMN	FRA	8641.40
OMN	GAB	10.78
OMN	DEU	19822.90
OMN	ISL	326.20
OMN	IRL	1628.32
OMN	ITA	3033.90
OMN	JPN	1096.02
OMN	KOR	788.80
OMN	LVA	131.74
OMN	LTU	106.96
OMN	MUS	140.82
OMN	NZL	10272.90
OMN	PRT	1498.60
OMN	QAT	738.66
OMN	RWA	696.76
OMN	SVK	781.76
OMN	LKA	2413.66
OMN	CHE	9400.20
OMN	SYR	399.36
OMN	TGO	0.10
OMN	TKM	400.00
OMN	USA	25247.30
OMN	URY	6820.92
OMN	ZMB	25.84
PAK	BEL	32.53
PAK	CHN	5450.25
PAK	HKG	64.63
PAK	IND	294.95
PAK	IRN	228.90
PAK	JPN	2399.78
PAK	JOR	41.40
PAK	KOR	3489.10
PAK	KWT	43.18
PAK	LBY	30.75
PAK	MDV	298.03
PAK	NZL	38.48
PAK	PAN	50.50
PAK	ROU	96.58
PAK	SEN	239.13
PAK	SVK	306.65
PAK	LKA	186.45
PAK	TLS	2422.70
PAK	USA	87.50
PAK	URY	1820.83
PAK	MKD	86.44
PAN	AFG	753.34
PAN	AUS	122.10
PAN	BHR	41.33
PAN	BGD	228.88
PAN	BEL	314.44
PAN	BDI	3.44
PAN	KHM	5.48
PAN	CAN	199.18
PAN	CHN	423.26
PAN	COM	2.26
PAN	FRA	353.72
PAN	GMB	1.50
PAN	DEU	684.22

PAN	HKG	604.48
PAN	IND	239.52
PAN	IRN	137.66
PAN	ITA	564.22
PAN	JPN	140.26
PAN	KEN	68.00
PAN	KOR	189.60
PAN	LSO	0.48
PAN	MWI	28.92
PAN	MEX	38.42
PAN	MMR	25.48
PAN	NLD	6.63
PAN	NZL	389.96
PAN	NGA	13.10
PAN	NOR	99.54
PAN	PAK	65.82
PAN	QAT	121.92
PAN	WSM	2.24
PAN	SEN	353.58
PAN	ESP	211.66
PAN	LKA	387.86
PAN	KNA	151.62
PAN	TKM	300.94
PAN	GBR	1358.54
PAN	USA	924.42
PAN	URY	3610.00
PNG	ATG	3.70
PNG	ARM	33.10
PNG	BHS	11.55
PNG	BEL	31.94
PNG	BLZ	29.26
PNG	CHN	21.92
PNG	COL	14.78
PNG	CRI	143.06
PNG	DOM	12.82
PNG	ECU	291.16
PNG	SLV	165.54
PNG	DEU	3.98
PNG	GTM	321.86
PNG	GUY	5.48
PNG	HND	143.20
PNG	HKG	6.16
PNG	IND	8.64
PNG	ITA	16.36
PNG	JAM	44.84
PNG	JPN	3.96
PNG	MDA	12.54
PNG	NZL	56.88
PNG	NER	18.90
PNG	QAT	17.58
PNG	LKA	63.14
PNG	LCA	0.76
PNG	VCT	3.46
PNG	SWZ	9.98
PNG	CHE	55.94
PNG	USA	25.38
PNG	URY	420.20
PNG	VNM	682.70
PRY	AUS	1506.64
PRY	BEL	0.20
PRY	CHN	0.10
PRY	HKG	1.20
PRY	IND	0.20

PRY	IDN	1.60
PRY	JPN	1.20
PRY	KIR	1.50
PRY	KOR	4.00
PRY	MDV	1.80
PRY	NZL	0.30
PRY	NIC	1.70
PRY	POL	0.10
PRY	RWA	0.10
PRY	STP	0.48
PRY	ZAF	1.30
PRY	TTO	0.50
PRY	USA	3.00
PRY	URY	1.50
PRY	VEN	3.75
PER	AGO	11.18
PER	ARG	538.44
PER	BOL	27.98
PER	BRA	389.76
PER	CHL	88.84
PER	CHN	35.34
PER	FRA	10.04
PER	DEU	17.04
PER	HKG	8.38
PER	ISR	11.40
PER	ITA	33.82
PER	JPN	19.52
PER	LBN	7.20
PER	NZL	33.84
PER	PHL	102.32
PER	RWA	103.12
PER	ESP	8.08
PER	LKA	13.86
PER	SYR	59.18
PER	TLS	11.56
PER	URY	58.36
PER	UZB	371.56
PER	VNM	23.80
PHL	BEL	311.90
PHL	BOL	161.60
PHL	BRA	557.92
PHL	BGR	113.44
PHL	CAN	985.60
PHL	CHL	1228.80
PHL	CHN	1815.06
PHL	COL	383.48
PHL	ECU	310.56
PHL	FIN	128.94
PHL	FRA	145.02
PHL	DEU	578.46
PHL	HND	33.28
PHL	IND	91.14
PHL	ITA	491.06
PHL	JPN	991.82
PHL	KOR	408.36
PHL	MDA	265.88
PHL	NZL	485.48
PHL	PNG	263.70
PHL	LKA	602.48
PHL	SYR	1152.66
PHL	USA	600.60
PHL	URY	4454.08
PHL	VNM	356.10

POL	AUS	472.04
POL	BEL	442.32
POL	BRN	3.53
POL	KHM	4.06
POL	CAN	281.92
POL	CHN	13805.70
POL	FRA	214.42
POL	DEU	1586.88
POL	HKG	5091.60
POL	HUN	93.62
POL	IND	128.30
POL	IDN	407.24
POL	IRL	120.02
POL	ITA	163.60
POL	JPN	7938.50
POL	KIR	0.10
POL	KOR	1404.86
POL	LSO	0.56
POL	MDV	2978.70
POL	MRT	25.68
POL	MDA	1080.78
POL	NZL	3891.18
POL	PRY	3.20
POL	STP	1.02
POL	SVK	4632.98
POL	LKA	108.36
POL	TLS	1812.46
POL	TGO	0.20
POL	TTO	0.18
POL	GBR	139.00
POL	USA	530.00
POL	URY	7844.80
POL	YEM	385.66
PRT	AUT	1766.20
PRT	BLR	751.66
PRT	BEL	2695.98
PRT	BIH	105.34
PRT	BGR	342.38
PRT	CHN	628.70
PRT	HRV	347.80
PRT	CZE	4630.48
PRT	DNK	1918.06
PRT	EST	384.12
PRT	FIN	682.54
PRT	FRA	5706.10
PRT	DEU	26039.80
PRT	HUN	2613.48
PRT	ISL	62.38
PRT	ITA	5874.92
PRT	KAZ	236.88
PRT	KGZ	11.64
PRT	LVA	640.62
PRT	LTU	1533.70
PRT	LUX	78.44
PRT	MDG	95.88
PRT	MNG	62.38
PRT	MNE	11.10
PRT	NZL	3756.10
PRT	OMN	1788.02
PRT	QAT	354.56
PRT	RUS	1269.52
PRT	RWA	3885.36
PRT	SYC	202.73

PRT	SVN	1838.56
PRT	SLB	322.86
PRT	LKA	2375.46
PRT	CHE	3043.58
PRT	SYR	680.00
PRT	TKM	1146.86
PRT	ARE	3122.84
PRT	USA	5230.02
PRT	URY	1792.66
QAT	AGO	1277.96
QAT	AUT	214.16
QAT	BEL	1365.10
QAT	BRA	245.90
QAT	CPV	212.30
QAT	CHN	200.96
QAT	DNK	300.16
QAT	FIN	246.94
QAT	FRA	5093.50
QAT	DEU	5185.22
QAT	IRL	205.62
QAT	ITA	1664.98
QAT	LUX	37.74
QAT	MDV	189.26
QAT	MOZ	254.76
QAT	MMR	84.12
QAT	NZL	1308.20
QAT	PRT	223.32
QAT	SAU	28.82
QAT	SVK	577.04
QAT	LKA	10315.60
QAT	CHE	455.66
QAT	SYR	332.72
QAT	TGO	1.70
QAT	TKM	230.30
QAT	USA	3107.40
QAT	URY	2196.94
ROU	AUS	118.48
ROU	BHR	49.46
ROU	CHN	271.72
ROU	EGY	74.58
ROU	IND	762.00
ROU	IRN	31.98
ROU	ITA	41.64
ROU	JPN	11360.00
ROU	JOR	31.60
ROU	KOR	5565.58
ROU	KWT	43.58
ROU	NZL	96.16
ROU	NIC	179.74
ROU	PAN	307.64
ROU	POL	193.30
ROU	SEN	219.40
ROU	SVK	2476.88
ROU	ESP	91.42
ROU	LKA	320.96
ROU	TLS	1125.26
ROU	GBR	813.72
ROU	URY	242.56
RUS	ALB	22.98
RUS	ARM	31.38
RUS	AUT	875.62
RUS	BEL	481.08
RUS	BIH	106.64

RUS	BGR	737.86
RUS	CMR	18.68
RUS	CPV	204.94
RUS	CHN	223.18
RUS	HRV	194.70
RUS	CZE	290.80
RUS	EGY	194.10
RUS	FRA	2178.96
RUS	GEO	39.24
RUS	DEU	4416.34
RUS	GRC	596.14
RUS	HUN	1301.38
RUS	IND	229.12
RUS	ITA	5493.26
RUS	LBN	63.44
RUS	MDG	76.14
RUS	MNG	345.50
RUS	NZL	750.76
RUS	OMN	225.76
RUS	PRT	467.92
RUS	RWA	272.14
RUS	SYC	464.35
RUS	SVN	201.60
RUS	SLB	133.28
RUS	LKA	628.00
RUS	SYR	187.64
RUS	TJK	92.90
RUS	TKM	2139.70
RUS	ARE	313.62
RUS	GBR	286.06
RUS	USA	1487.28
RUS	URY	809.78
RWA	AFG	156.60
RWA	ALB	103.68
RWA	DZA	298.46
RWA	ARG	217.36
RWA	ARM	280.78
RWA	AUT	1701.56
RWA	AZE	771.20
RWA	BGD	153.92
RWA	BLR	11845.10
RWA	BEL	4824.10
RWA	BIH	142.90
RWA	BRA	947.46
RWA	BGR	2750.30
RWA	BFA	16.36
RWA	BDI	4.24
RWA	CHN	14997.70
RWA	CIV	63.80
RWA	HRV	1673.88
RWA	CYP	4929.54
RWA	CZE	4142.24
RWA	DNK	1133.54
RWA	ECU	84.28
RWA	EGY	718.72
RWA	SLV	44.84
RWA	EST	1190.90
RWA	ETH	37.50
RWA	FIN	8180.32
RWA	FRA	10769.30
RWA	GEO	387.70
RWA	DEU	28062.80
RWA	GRC	3711.48

RWA	HND	26.32
RWA	HUN	4776.20
RWA	ISL	47.76
RWA	IND	2600.88
RWA	IDN	331.80
RWA	IRN	946.80
RWA	ISR	980.54
RWA	ITA	16526.20
RWA	JPN	6670.70
RWA	JOR	127.88
RWA	KAZ	7052.46
RWA	KEN	59.53
RWA	KOR	4336.08
RWA	KGZ	495.94
RWA	LVA	817.30
RWA	LBN	346.84
RWA	LTU	3687.86
RWA	LUX	51.20
RWA	MDG	413.34
RWA	MLT	9.98
RWA	MUS	8.34
RWA	MNG	315.74
RWA	MNE	459.88
RWA	MOZ	1164.44
RWA	NZL	25050.00
RWA	NER	22.30
RWA	NOR	153.67
RWA	OMN	1365.94
RWA	PAN	268.18
RWA	PRY	8.52
RWA	POL	297.34
RWA	PRT	9415.18
RWA	QAT	674.64
RWA	RUS	3197.64
RWA	SRB	27.26
RWA	SYC	1958.78
RWA	SVN	3876.90
RWA	SLB	449.40
RWA	LKA	6874.98
RWA	CHE	3113.24
RWA	SYR	9759.36
RWA	TJK	413.84
RWA	TLS	1274.44
RWA	TGO	0.10
RWA	TUR	471.50
RWA	TKM	13708.80
RWA	ARE	12753.30
RWA	USA	8135.68
RWA	URY	15749.00
RWA	UZB	229.40
RWA	YEM	577.26
WSM	BEL	19.96
WSM	BDI	2.96
WSM	CHN	1.42
WSM	COD	4.14
WSM	CYP	0.44
WSM	DJI	0.34
WSM	FRA	0.64
WSM	DEU	1.32
WSM	HKG	11.76
WSM	IND	0.34
WSM	ITA	0.58
WSM	JPN	0.32

WSM	KEN	27.76
WSM	MDV	0.40
WSM	NZL	0.56
WSM	PAN	0.54
WSM	ESP	1.34
WSM	SUR	0.44
WSM	SWE	2.08
WSM	SYR	9.28
WSM	THA	1.28
WSM	UKR	4.94
WSM	USA	22.32
WSM	URY	3.62
STP	AUS	54.68
STP	BGD	0.02
STP	CAN	0.06
STP	DNK	0.04
STP	FJI	0.30
STP	FRA	0.04
STP	DEU	0.10
STP	HKG	0.10
STP	ITA	0.18
STP	JPN	1.26
STP	NIC	2.10
STP	SVK	0.04
STP	TZA	0.08
STP	TTO	0.24
STP	USA	0.94
STP	URY	12.72
SAU	AGO	0.10
SAU	BHS	0.02
SAU	BEL	0.60
SAU	CMR	0.02
SAU	FRA	0.18
SAU	GAB	0.02
SAU	JPN	0.02
SAU	NZL	1.94
SAU	NOR	0.02
SAU	PNG	0.02
SAU	QAT	1.38
SAU	ESP	0.02
SAU	LKA	0.02
SAU	URY	0.04
SEN	BHR	2881.46
SEN	BEL	1447.06
SEN	BRA	1358.38
SEN	BFA	8.68
SEN	BDI	24.00
SEN	CAN	1252.34
SEN	CPV	1.54
SEN	CHN	11517.00
SEN	COM	0.46
SEN	COD	5.80
SEN	ECU	52.56
SEN	EGY	1706.88
SEN	ERI	55.53
SEN	ETH	547.76
SEN	FRA	3965.48
SEN	GRC	1760.58
SEN	IND	6710.04
SEN	IDN	2586.90
SEN	IRN	290.65
SEN	ITA	4340.68
SEN	JPN	27823.20

SEN	JOR	2105.12
SEN	KEN	332.43
SEN	KOR	15777.70
SEN	LBN	239.26
SEN	MYS	6.92
SEN	MDV	1487.50
SEN	MEX	80.40
SEN	MOZ	1276.82
SEN	MMR	17.56
SEN	NLD	27.30
SEN	NZL	4199.18
SEN	NIC	260.88
SEN	PAK	253.68
SEN	PAN	2636.22
SEN	POL	2291.00
SEN	QAT	505.32
SEN	ROU	505.72
SEN	WSM	11.28
SEN	SLE	152.60
SEN	SVK	7301.96
SEN	ESP	2977.38
SEN	LKA	3109.26
SEN	KNA	103.10
SEN	SUR	726.32
SEN	TJK	328.96
SEN	THA	123.94
SEN	TLS	3384.50
SEN	TON	7.45
SEN	TUR	118.28
SEN	TKM	1755.28
SEN	UKR	29.94
SEN	GBR	4527.78
SEN	URY	28248.90
SEN	MKD	469.58
SRB	BEL	9.60
SRB	BEN	20.76
SRB	BFA	21.88
SRB	CMR	14.76
SRB	CPV	3.00
SRB	CAF	0.68
SRB	CHN	10.04
SRB	COD	9.20
SRB	CIV	43.10
SRB	FRA	131.32
SRB	GAB	5.02
SRB	GMB	69.82
SRB	GRC	24.14
SRB	GIN	42.66
SRB	IND	140.70
SRB	ITA	80.64
SRB	JPN	10.86
SRB	MLT	250.00
SRB	MUS	39.84
SRB	MOZ	10.48
SRB	NZL	14.76
SRB	QAT	9.54
SRB	LKA	74.82
SRB	SUR	9.16
SRB	SYR	21.98
SRB	TON	17.04
SRB	GBR	8.66
SYC	ALB	93.75
SYC	AUT	174.78

SYC	BEL	75.00
SYC	BIH	865.40
SYC	BGR	131.55
SYC	HRV	231.58
SYC	CZE	81.15
SYC	FRA	205.50
SYC	DEU	590.45
SYC	GRC	143.85
SYC	HUN	172.15
SYC	ITA	780.25
SYC	MDG	365.60
SYC	MAR	783.55
SYC	NZL	95.83
SYC	PRT	70.60
SYC	RUS	173.68
SYC	RWA	284.98
SYC	SVN	53.85
SYC	SLB	395.25
SYC	LKA	56.50
SYC	SYR	41.20
SYC	TKM	52.55
SYC	ARE	95.95
SYC	USA	108.20
SYC	URY	63.10
SLE	AUS	0.40
SLE	BEL	0.30
SLE	DNK	1.00
SLE	FIN	0.52
SLE	FRA	61.18
SLE	DEU	13.82
SLE	GHA	0.22
SLE	GRC	0.40
SLE	HKG	0.72
SLE	IRL	1.52
SLE	ITA	27.98
SLE	JPN	1.04
SLE	MDV	0.32
SLE	MRT	0.42
SLE	MEX	0.48
SLE	NZL	5.58
SLE	POL	0.58
SLE	SEN	103.28
SLE	SVK	1.02
SLE	ESP	1.64
SLE	KNA	1.50
SLE	SYR	0.38
SLE	GBR	12.40
SLE	USA	89.52
SLE	URY	1.28
SVK	AFG	1.66
SVK	AUS	8218.44
SVK	AZE	83.82
SVK	BGD	533.34
SVK	BEL	1012.16
SVK	BWA	10.62
SVK	BRA	810.44
SVK	BRN	271.03
SVK	KHM	123.28
SVK	CAN	863.60
SVK	CHN	20134.50
SVK	COM	0.80
SVK	COD	4.90
SVK	CIV	83.48

SVK	ERI	4.45
SVK	FJI	387.42
SVK	FRA	3723.28
SVK	GAB	10.70
SVK	GMB	3.82
SVK	DEU	5885.28
SVK	GUY	4.62
SVK	HKG	20219.80
SVK	HUN	569.32
SVK	IND	6162.56
SVK	IDN	22244.50
SVK	IRN	430.45
SVK	IRL	1303.42
SVK	ISR	353.44
SVK	JPN	12605.50
SVK	KEN	246.55
SVK	KIR	1.90
SVK	KOR	8167.48
SVK	LSO	6.92
SVK	LBR	901.82
SVK	MDV	30905.40
SVK	MLI	185.16
SVK	MRT	193.90
SVK	MUS	3.56
SVK	MEX	46.84
SVK	MDA	1963.34
SVK	MNE	21.66
SVK	MMR	13.56
SVK	NPL	15.38
SVK	NLD	119.23
SVK	NZL	5341.26
SVK	NIC	1126.90
SVK	NOR	312.67
SVK	PAK	85.30
SVK	PAN	464.60
SVK	PNG	2180.00
SVK	PRY	85.68
SVK	PER	15.92
SVK	POL	4508.92
SVK	ROU	91.46
SVK	STP	4.96
SVK	SLE	55.80
SVK	ESP	566.84
SVK	KNA	567.30
SVK	SWE	10.62
SVK	THA	67.70
SVK	TLS	9396.72
SVK	TGO	15.00
SVK	TTO	7.50
SVK	TUN	23.94
SVK	UKR	26.18
SVK	GBR	2743.46
SVK	USA	6521.28
SVK	URY	24406.50
SVK	VEN	16.85
SVK	YEM	4394.86
SVK	MKD	60.40
SVN	AUT	2393.36
SVN	BEL	731.78
SVN	BGR	140.12
SVN	CHN	200.74
SVN	HRV	187.62
SVN	CZE	4799.84

SVN	DNK	303.62
SVN	FIN	280.70
SVN	FRA	1749.40
SVN	DEU	9598.88
SVN	GRC	174.84
SVN	HUN	2030.48
SVN	ITA	2388.90
SVN	LVA	65.04
SVN	MNG	13.06
SVN	NZL	1273.64
SVN	PRT	2147.00
SVN	RUS	549.36
SVN	RWA	618.34
SVN	SYC	188.38
SVN	SLB	311.04
SVN	LKA	910.34
SVN	CHE	441.90
SVN	SYR	306.46
SVN	TKM	343.78
SVN	ARE	451.24
SVN	USA	1329.98
SVN	URY	1249.84
SLB	ALB	23.30
SLB	AUT	1490.30
SLB	BEL	192.44
SLB	BIH	622.70
SLB	HRV	1632.76
SLB	CZE	419.04
SLB	DNK	187.22
SLB	FRA	1275.34
SLB	DEU	3815.50
SLB	HUN	457.68
SLB	ITA	2449.62
SLB	MDG	171.06
SLB	NZL	289.22
SLB	PRT	537.82
SLB	RUS	227.80
SLB	RWA	637.14
SLB	SYC	868.50
SLB	SVN	286.36
SLB	LKA	325.46
SLB	CHE	184.58
SLB	SYR	207.92
SLB	TKM	161.06
SLB	ARE	182.98
SLB	USA	478.42
SLB	URY	450.98
ZAF	VEN	0.35
ESP	AGO	586.48
ESP	AUS	1131.08
ESP	BEL	1354.10
ESP	BEN	19.56
ESP	BWA	2932.50
ESP	BFA	17.14
ESP	BDI	7.04
ESP	CMR	40.50
ESP	CAN	428.82
ESP	CPV	2.82
ESP	CAF	0.53
ESP	CHN	1918.38
ESP	COM	6.78
ESP	COD	13.80
ESP	CIV	54.00

ESP	ERI	4.85
ESP	FJI	2.80
ESP	FRA	1070.04
ESP	GAB	16.12
ESP	GMB	1.68
ESP	DEU	3611.26
ESP	GHA	213.82
ESP	HKG	625.98
ESP	IND	2430.26
ESP	ISR	670.58
ESP	ITA	1208.40
ESP	JPN	5619.86
ESP	KEN	520.27
ESP	KOR	855.26
ESP	LSO	738.18
ESP	MWI	103.64
ESP	MYS	364.70
ESP	MLI	3.18
ESP	MLT	57.06
ESP	MUS	8.86
ESP	MEX	284.94
ESP	MOZ	217.24
ESP	MMR	940.20
ESP	NPL	2149.90
ESP	NZL	2259.02
ESP	NGA	16.96
ESP	NOR	516.16
ESP	WSM	15.64
ESP	SAU	0.16
ESP	SRB	48.90
ESP	SLE	49.00
ESP	LKA	1313.60
ESP	SUR	54.46
ESP	SWE	1283.46
ESP	SYR	1243.44
ESP	THA	436.06
ESP	TON	16.60
ESP	TKM	1313.26
ESP	UKR	149.42
ESP	GBR	469.28
ESP	USA	6326.18
ESP	URY	5402.76
ESP	ZMB	1261.36
ESP	ZWE	1044.98
LKA	ALB	43.70
LKA	DZA	1327.72
LKA	ARG	595.86
LKA	AUS	1031.86
LKA	AUT	1861.44
LKA	BHS	4.75
LKA	BHR	48.70
LKA	BRB	8.00
LKA	BLR	79.58
LKA	BEL	6045.92
LKA	BEN	16.90
LKA	BOL	36.14
LKA	BIH	49.36
LKA	BRA	1362.02
LKA	BRN	3.63
LKA	BGR	345.52
LKA	BFA	17.50
LKA	CMR	32.10
LKA	CPV	24.62

LKA	CAF	0.58
LKA	CHL	671.38
LKA	CHN	1937.70
LKA	COL	340.08
LKA	COM	0.82
LKA	COD	5.70
LKA	CRI	122.68
LKA	CIV	83.60
LKA	HRV	330.40
LKA	CYP	221.32
LKA	CZE	1562.68
LKA	DNK	1433.02
LKA	ECU	145.90
LKA	EGY	204.14
LKA	SLV	80.20
LKA	EST	106.44
LKA	FIN	941.30
LKA	FRA	38116.70
LKA	GAB	27.52
LKA	GMB	4.20
LKA	DEU	23292.80
LKA	GHA	99.22
LKA	GRC	2340.42
LKA	GTM	117.22
LKA	HND	58.86
LKA	HUN	1045.44
LKA	ISL	69.72
LKA	IRN	332.20
LKA	IRL	1055.78
LKA	ISR	690.32
LKA	ITA	17799.30
LKA	JAM	29.20
LKA	JPN	1479.70
LKA	LVA	111.16
LKA	LBN	175.98
LKA	LBY	92.90
LKA	LTU	208.92
LKA	LUX	164.12
LKA	MDG	38.04
LKA	MWI	31.38
LKA	MLT	10.60
LKA	MRT	121.78
LKA	MUS	23.02
LKA	MEX	67.80
LKA	MDA	3360.34
LKA	MNG	16.28
LKA	MNE	4.60
LKA	MOZ	3022.90
LKA	MMR	24.42
LKA	NPL	36.42
LKA	NZL	6560.02
LKA	NER	34.48
LKA	NOR	138.93
LKA	OMN	1182.82
LKA	PAK	95.08
LKA	PNG	73.20
LKA	PER	27.78
LKA	PHL	226.48
LKA	PRT	2452.22
LKA	QAT	18511.80
LKA	ROU	87.02
LKA	RUS	934.46
LKA	RWA	1639.98

LKA	SAU	0.08
LKA	SEN	741.54
LKA	SRB	125.26
LKA	SLE	51.72
LKA	SVN	670.76
LKA	SLB	544.06
LKA	ESP	842.20
LKA	SWZ	3.30
LKA	SWE	1.38
LKA	CHE	1922.00
LKA	SYR	3010.36
LKA	TGO	0.05
LKA	TON	18.68
LKA	TUN	51.42
LKA	TUR	703.68
LKA	TKM	3393.62
LKA	GBR	577.60
LKA	USA	17402.10
LKA	URY	8262.32
LKA	UZB	54.80
LKA	VNM	505.48
KNA	AUS	53.12
KNA	BEL	200.94
KNA	CAN	67.52
KNA	FJI	2.46
KNA	FRA	101.72
KNA	DEU	233.80
KNA	HKG	64.54
KNA	IND	285.60
KNA	IRN	46.32
KNA	ISR	41.82
KNA	ITA	120.18
KNA	JPN	156.32
KNA	MLI	61.64
KNA	MDA	38.16
KNA	NZL	90.82
KNA	PAN	34.42
KNA	RWA	138.20
KNA	SEN	31.98
KNA	SVK	68.66
KNA	LKA	36.54
KNA	TJK	63.26
KNA	TKM	56.24
KNA	GBR	126.76
KNA	USA	671.20
KNA	URY	1862.68
LCA	ALB	0.04
LCA	ATG	0.22
LCA	BRB	0.12
LCA	CAN	0.12
LCA	CRI	0.02
LCA	DOM	0.26
LCA	FRA	0.18
LCA	GRD	0.06
LCA	JPN	0.18
LCA	VCT	0.16
LCA	SDN	0.12
LCA	CHE	0.06
LCA	TUN	0.42
LCA	USA	3.72
LCA	URY	31.32
VCT	ATG	1.43
VCT	BHS	0.18

VCT	BRB	6.13
VCT	BLZ	0.13
VCT	CAN	0.25
VCT	CHN	0.08
VCT	DOM	4.05
VCT	FRA	1.20
VCT	DEU	0.38
VCT	GRD	2.83
VCT	GUY	0.63
VCT	JAM	0.70
VCT	ESP	0.13
VCT	LCA	0.93
VCT	SDN	2.73
VCT	SWZ	0.08
VCT	TUN	15.73
VCT	USA	19.33
VCT	URY	12.78
VCT	VNM	0.20
SDN	ATG	2.72
SDN	BHS	0.02
SDN	BRB	4.80
SDN	CAN	0.12
SDN	DOM	1.78
SDN	FRA	0.20
SDN	GRD	1.74
SDN	GUY	0.14
SDN	JAM	1.02
SDN	NZL	0.04
SDN	LCA	1.16
SDN	VCT	4.18
SDN	SWZ	0.06
SDN	CHE	0.02
SDN	TUN	4.44
SDN	USA	11.80
SDN	URY	2.92
SDN	VNM	0.14
SUR	BGD	14.88
SUR	BEL	6.23
SUR	CAN	32.70
SUR	CHN	2895.90
SUR	EGY	59.38
SUR	ERI	5.38
SUR	FRA	19.08
SUR	DEU	30.50
SUR	HKG	6.48
SUR	IND	25.50
SUR	IDN	17.00
SUR	ITA	14.08
SUR	JPN	303.53
SUR	JOR	6.98
SUR	KOR	18.00
SUR	LBN	20.88
SUR	MDA	9.20
SUR	PAN	11.20
SUR	SEN	178.20
SUR	SVK	37.38
SUR	TJK	14.28
SUR	TLS	8.78
SUR	TUR	6.58
SUR	TKM	6.60
SUR	GBR	136.30
SUR	USA	69.88
SWZ	BRB	7.54

SWZ	GUY	4.64
SWZ	ISL	34.36
SWE	AGO	4.90
SWE	AUS	64.76
SWE	BWA	73.94
SWE	CHN	19.62
SWE	ETH	2.96
SWE	FRA	1.70
SWE	IRN	2.04
SWE	ITA	2.26
SWE	JPN	7.30
SWE	KEN	22.22
SWE	MWI	6.46
SWE	MYS	92.46
SWE	MEX	7.00
SWE	MMR	57.94
SWE	NPL	5.64
SWE	NZL	3.94
SWE	NIC	23.66
SWE	ESP	999.12
SWE	THA	9.94
SWE	TLS	18.22
SWE	UKR	19.58
SWE	USA	39.24
SWE	URY	112.22
SWE	VEN	0.40
SWE	ZMB	10.00
SWE	ZWE	15.76
CHE	DZA	276.36
CHE	ARG	222.14
CHE	AUS	1581.10
CHE	AUT	1544.64
CHE	AZE	55.58
CHE	BGD	148.34
CHE	BRB	16.70
CHE	BLR	107.14
CHE	BEL	6806.94
CHE	BOL	33.20
CHE	BIH	57.00
CHE	BWA	72.62
CHE	BRA	934.84
CHE	BGR	203.46
CHE	CAN	1810.68
CHE	CPV	4.06
CHE	CHL	364.70
CHE	CHN	2695.74
CHE	HRV	229.90
CHE	CYP	78.72
CHE	CZE	982.66
CHE	DNK	10521.40
CHE	EGY	194.98
CHE	SLV	39.46
CHE	EST	962.52
CHE	ETH	61.88
CHE	FJI	2.18
CHE	FIN	8179.10
CHE	FRA	6605.62
CHE	GAB	8.20
CHE	GMB	1.08
CHE	DEU	14612.20
CHE	GHA	91.96
CHE	GRC	710.98
CHE	GRD	1.46

CHE	GTM	62.36
CHE	HND	46.32
CHE	HUN	899.84
CHE	ISL	385.10
CHE	IND	1174.78
CHE	IDN	498.16
CHE	IRN	758.63
CHE	IRL	694.70
CHE	ISR	401.42
CHE	ITA	4796.72
CHE	JAM	30.52
CHE	JPN	2123.40
CHE	KAZ	235.26
CHE	KGZ	7.00
CHE	LVA	508.04
CHE	LBY	46.53
CHE	LTU	570.44
CHE	LUX	82.24
CHE	MDV	604.70
CHE	MLI	4.36
CHE	MDA	888.20
CHE	MNG	16.52
CHE	MOZ	307.26
CHE	NPL	4.64
CHE	NZL	6505.52
CHE	NIC	230.38
CHE	NER	17.86
CHE	OMN	12021.10
CHE	PAK	92.80
CHE	PAN	271.78
CHE	PER	26.54
CHE	PHL	94.10
CHE	PRT	2684.64
CHE	QAT	671.08
CHE	ROU	56.04
CHE	RUS	443.14
CHE	RWA	2238.68
CHE	STP	0.28
CHE	SEN	922.48
CHE	SYC	197.83
CHE	SVK	855.88
CHE	SVN	297.80
CHE	SLB	176.44
CHE	ESP	773.38
CHE	LKA	4004.30
CHE	KNA	67.92
CHE	LCA	0.70
CHE	VCT	2.74
CHE	SDN	1.42
CHE	SUR	152.28
CHE	SWE	1.48
CHE	SYR	1323.24
CHE	THA	58.80
CHE	TUR	141.66
CHE	TKM	1313.42
CHE	UKR	38.46
CHE	ARE	473.70
CHE	USA	10003.40
CHE	URY	13281.20
CHE	UZB	20.48
CHE	ZMB	42.18
CHE	ZWE	15.40
SYR	ALB	67.06

SYR	DZA	184.00
SYR	ATG	3.63
SYR	ARG	236.78
SYR	ARM	46.44
SYR	AUS	1187.04
SYR	AUT	4500.40
SYR	BHS	11.80
SYR	BHR	85.60
SYR	BRB	13.40
SYR	BLR	107.64
SYR	BEL	2513.14
SYR	BLZ	2.10
SYR	BEN	9.96
SYR	BOL	8.18
SYR	BIH	134.18
SYR	BWA	19.94
SYR	BRA	1353.84
SYR	BRN	3.17
SYR	BGR	224.86
SYR	KHM	8.54
SYR	CAN	1822.72
SYR	CPV	1.32
SYR	CAF	0.40
SYR	CHN	2984.94
SYR	COL	223.66
SYR	CRI	78.58
SYR	CIV	34.08
SYR	HRV	269.24
SYR	CYP	63.94
SYR	CZE	1150.20
SYR	DNK	843.02
SYR	ERI	4.48
SYR	FJI	3.16
SYR	FIN	576.04
SYR	FRA	11712.70
SYR	GEO	31.14
SYR	DEU	30139.90
SYR	GRC	870.38
SYR	GTM	95.14
SYR	HKG	3684.50
SYR	HUN	763.40
SYR	ISL	86.82
SYR	IND	7068.40
SYR	IRN	1310.20
SYR	IRL	668.60
SYR	ISR	2579.44
SYR	ITA	12414.10
SYR	JAM	31.06
SYR	JPN	4870.40
SYR	JOR	107.74
SYR	KAZ	143.28
SYR	KEN	83.55
SYR	KIR	0.90
SYR	KOR	1168.04
SYR	KGZ	5.76
SYR	LVA	117.18
SYR	LBN	328.66
SYR	LBY	81.57
SYR	LTU	87.24
SYR	LUX	183.16
SYR	MDG	62.42
SYR	MDV	1193.26
SYR	MLI	3.48

SYR	MRT	64.86
SYR	MEX	45.80
SYR	MDA	1050.84
SYR	MOZ	234.94
SYR	NPL	22.64
SYR	NLD	51.60
SYR	NZL	4288.02
SYR	NIC	169.22
SYR	NOR	144.47
SYR	OMN	624.66
SYR	PAK	87.12
SYR	PAN	357.92
SYR	PRY	3.64
SYR	PER	104.00
SYR	POL	293.56
SYR	PRT	1254.82
SYR	QAT	434.54
SYR	ROU	120.80
SYR	RUS	394.16
SYR	RWA	1451.76
SYR	WSM	5.06
SYR	STP	0.20
SYR	SAU	0.04
SYR	SEN	1076.08
SYR	SYC	166.75
SYR	SLE	2.70
SYR	SVK	2150.74
SYR	SVN	328.16
SYR	SLB	275.76
SYR	ESP	594.00
SYR	LKA	5135.72
SYR	KNA	106.86
SYR	LCA	0.64
SYR	VCT	3.44
SYR	SUR	67.36
SYR	SWE	4.36
SYR	CHE	1366.12
SYR	THA	78.10
SYR	TLS	1142.00
SYR	TGO	0.05
SYR	TTO	0.16
SYR	TUR	149.34
SYR	TKM	3943.08
SYR	GBR	1739.38
SYR	USA	6842.76
SYR	URY	14397.40
SYR	UZB	19.30
SYR	VNM	175.74
SYR	YEM	674.48
SYR	MKD	349.68
SYR	ZMB	18.04
SYR	ZWE	26.00
TJK	DZA	103.90
TJK	HRV	58.62
TJK	CYP	74.38
TJK	EGY	160.88
TJK	FRA	974.46
TJK	DEU	98.56
TJK	ITA	1883.68
TJK	JOR	240.60
TJK	KWT	101.86
TJK	LBN	263.44
TJK	LBY	71.38

TJK	NZL	143.80
TJK	QAT	45.98
TJK	ROU	30.36
TJK	SEN	513.10
TJK	LKA	260.14
TJK	SUR	38.90
TJK	TKM	389.22
TJK	ARE	36.22
TJK	GBR	91.86
TJK	USA	222.42
TJK	URY	210.10
TJK	MKD	34.44
THA	BEL	33.64
THA	BDI	28.12
THA	CAN	11.40
THA	CHN	95.98
THA	COM	1.44
THA	COD	51.74
THA	FRA	25.10
THA	DEU	71.44
THA	HKG	10.96
THA	IND	80.12
THA	ITA	36.92
THA	JPN	74.70
THA	KEN	97.96
THA	MYS	37.98
THA	NZL	87.52
THA	PAN	12.62
THA	WSM	23.86
THA	SEN	13.14
THA	SVK	11.92
THA	ESP	186.68
THA	LKA	12.68
THA	SYR	197.32
THA	UKR	40.68
THA	GBR	43.34
THA	USA	219.02
THA	URY	25.98
THA	ZMB	39.22
TLS	ATG	2.50
TLS	ARG	264.58
TLS	AUS	4014.06
TLS	BHR	52.00
TLS	BGD	332.02
TLS	BRB	8.34
TLS	BEL	1342.90
TLS	BEN	33.62
TLS	BWA	7.18
TLS	BRN	65.97
TLS	BFA	8.28
TLS	BDI	1.74
TLS	KHM	987.76
TLS	CMR	42.08
TLS	CAN	1730.74
TLS	CPV	5.76
TLS	CAF	0.38
TLS	CHL	230.70
TLS	CHN	14997.30
TLS	COM	0.32
TLS	COD	6.00
TLS	CIV	126.02
TLS	CYP	46.18
TLS	ECU	118.08

TLS	ETH	37.40
TLS	FJI	34.16
TLS	FRA	1351.86
TLS	GAB	17.34
TLS	GMB	1.80
TLS	DEU	2159.04
TLS	GHA	118.44
TLS	HKG	6259.34
TLS	IND	1546.90
TLS	IDN	3527.88
TLS	IRL	503.00
TLS	ISR	396.38
TLS	ITA	1382.86
TLS	JAM	25.70
TLS	JPN	15353.90
TLS	JOR	95.32
TLS	KEN	81.25
TLS	KIR	0.40
TLS	KOR	2806.98
TLS	LBN	72.64
TLS	MWI	28.38
TLS	MDV	6132.28
TLS	MLI	37.18
TLS	MLT	13.96
TLS	MUS	4.88
TLS	MEX	50.18
TLS	MDA	1538.90
TLS	MMR	34.44
TLS	NLD	36.70
TLS	NZL	2952.00
TLS	NIC	584.36
TLS	NGA	14.70
TLS	NOR	177.90
TLS	PAK	171.10
TLS	PAN	467.24
TLS	PRY	19.76
TLS	PER	28.50
TLS	POL	2206.90
TLS	ROU	102.96
TLS	STP	1.16
TLS	SEN	956.00
TLS	SRB	186.22
TLS	SLE	7.20
TLS	SVK	7668.34
TLS	ESP	922.06
TLS	LKA	961.64
TLS	KNA	151.98
TLS	VCT	6.08
TLS	SWZ	6.54
TLS	SYR	930.82
TLS	THA	34.64
TLS	TGO	1.20
TLS	TTO	0.80
TLS	TUN	47.22
TLS	GBR	1322.34
TLS	USA	3076.26
TLS	URY	20652.80
TLS	VEN	4.35
TLS	YEM	2509.52
TLS	MKD	66.58
TLS	ZWE	14.40
TGO	AUS	33.70
TGO	BGD	2.55

TGO	BRA	0.50
TGO	CAN	1.45
TGO	CAF	0.05
TGO	CIV	0.10
TGO	CYP	0.15
TGO	FJI	0.50
TGO	DEU	1.05
TGO	IDN	1.75
TGO	ITA	1.80
TGO	JPN	12.10
TGO	LBR	0.05
TGO	MDV	0.90
TGO	NZL	0.05
TGO	NIC	0.10
TGO	OMN	0.10
TGO	PAN	1.15
TGO	POL	0.05
TGO	QAT	7.45
TGO	SVK	0.60
TGO	SUR	0.15
TGO	TLS	0.15
TGO	URY	7.35
TON	AUS	3.60
TON	BEL	4.38
TON	BEN	41.45
TON	BRA	4.00
TON	BFA	72.30
TON	CHN	10.25
TON	CIV	5.28
TON	FRA	6.83
TON	DEU	3.25
TON	GHA	50.90
TON	IND	18.65
TON	IDN	4.85
TON	ITA	3.08
TON	MLT	108.82
TON	MOZ	3.75
TON	NZL	30.08
TON	NIC	4.48
TON	NGA	24.30
TON	NOR	109.77
TON	PAN	3.58
TON	SRB	6.25
TON	TLS	3.58
TON	URY	4.40
TTO	AUS	0.38
TTO	CAN	0.02
TTO	CHN	0.04
TTO	FJI	0.08
TTO	DEU	0.08
TTO	HKG	0.10
TTO	JPN	4.84
TTO	KOR	0.44
TTO	NIC	1.96
TTO	STP	0.16
TTO	SVK	0.02
TTO	USA	0.04
TTO	URY	3.32
TUN	ATG	48.62
TUN	BHS	42.56
TUN	BRB	320.76
TUN	BLZ	6.72
TUN	BRA	64.72

TUN	CAN	117.86
TUN	COL	133.82
TUN	DOM	162.86
TUN	FRA	305.04
TUN	DEU	72.02
TUN	GRD	81.36
TUN	GUY	227.06
TUN	HND	51.46
TUN	HUN	63.90
TUN	JAM	650.64
TUN	MDA	151.54
TUN	NZL	87.52
TUN	PNG	50.74
TUN	LKA	270.34
TUN	LCA	28.48
TUN	VCT	81.64
TUN	SDN	55.52
TUN	SWZ	184.18
TUN	USA	113.62
TUN	URY	5786.54
TUR	DZA	174.08
TUR	BEL	305.18
TUR	BRA	68.24
TUR	CHN	24.26
TUR	EGY	48.84
TUR	FRA	3713.22
TUR	DEU	959.20
TUR	GRC	22.54
TUR	IND	100.42
TUR	IRN	49.94
TUR	ITA	2570.16
TUR	JPN	24.72
TUR	LBY	498.14
TUR	MOZ	103.88
TUR	NZL	243.32
TUR	QAT	34.30
TUR	SEN	34.78
TUR	SRB	28.80
TUR	LKA	613.54
TUR	CHE	26.08
TUR	SYR	155.66
TUR	TKM	114.96
TUR	USA	362.48
TUR	URY	137.84
TKM	ALB	203.90
TKM	DZA	887.72
TKM	ARM	69.64
TKM	AUT	973.86
TKM	AZE	348.56
TKM	BHR	39.23
TKM	BLR	78.48
TKM	BEL	2332.84
TKM	BIH	232.78
TKM	BGR	1264.70
TKM	BDI	4.62
TKM	CMR	18.78
TKM	CPV	6.08
TKM	CHL	235.75
TKM	CIV	29.08
TKM	HRV	252.34
TKM	CYP	677.52
TKM	CZE	529.30
TKM	DNK	758.24

TKM	EGY	378.84
TKM	ERI	14.00
TKM	ETH	102.24
TKM	FRA	4700.46
TKM	GMB	1.98
TKM	GEO	369.56
TKM	DEU	10674.00
TKM	GRC	1449.96
TKM	IRN	942.64
TKM	IRL	518.68
TKM	ISR	1410.24
TKM	ITA	5649.18
TKM	JOR	253.70
TKM	KAZ	493.70
TKM	KGZ	36.64
TKM	LVA	58.04
TKM	LBN	225.28
TKM	LSO	1.74
TKM	LBY	79.87
TKM	LTU	119.40
TKM	MDG	121.18
TKM	MRT	49.86
TKM	MUS	4.48
TKM	MNG	98.18
TKM	MOZ	488.36
TKM	NZL	2338.22
TKM	OMN	412.00
TKM	PRT	1398.82
TKM	QAT	480.32
TKM	ROU	98.08
TKM	RUS	2110.64
TKM	RWA	2713.64
TKM	SAU	0.06
TKM	SEN	988.46
TKM	SRB	32.92
TKM	SYC	265.03
TKM	SLE	3.68
TKM	SVN	225.06
TKM	SLB	187.06
TKM	LKA	3785.74
TKM	SUR	122.52
TKM	CHE	821.42
TKM	TJK	318.52
TKM	TUR	325.52
TKM	ARE	888.88
TKM	GBR	1748.84
TKM	USA	6645.34
TKM	URY	4549.94
TKM	VEN	0.40
TKM	MKD	132.22
UGA	AFG	33.24
UGA	ARM	35.94
UGA	AZE	191.00
UGA	GEO	77.74
UGA	ARE	2919.90
UKR	AUS	5.24
UKR	BEL	33.14
UKR	BDI	22.46
UKR	CHN	6.50
UKR	COD	49.40
UKR	FRA	27.72
UKR	DEU	34.10
UKR	HKG	12.86

UKR	ISR	5.76
UKR	ITA	8.66
UKR	JPN	6.08
UKR	KEN	86.78
UKR	NZL	64.12
UKR	WSM	49.56
UKR	SVK	24.40
UKR	ESP	14.06
UKR	LKA	18.46
UKR	SUR	67.16
UKR	SYR	77.32
UKR	THA	15.54
UKR	GBR	98.90
UKR	USA	34.68
UKR	URY	15.62
ARE	ALB	81.22
ARE	DZA	487.32
ARE	ARM	113.54
ARE	AZE	340.08
ARE	BLR	913.20
ARE	BIH	73.98
ARE	BGR	919.44
ARE	CMR	19.70
ARE	CHN	702.28
ARE	CYP	68.06
ARE	CZE	591.44
ARE	EGY	616.20
ARE	EST	166.42
ARE	ETH	51.40
ARE	GEO	267.42
ARE	DEU	1501.32
ARE	HUN	908.12
ARE	IND	591.52
ARE	IDN	363.62
ARE	IRN	426.46
ARE	ITA	2000.06
ARE	JOR	236.94
ARE	KAZ	879.90
ARE	KGZ	39.48
ARE	LVA	152.60
ARE	LBN	125.78
ARE	LTU	223.60
ARE	MDG	88.42
ARE	MLT	17.46
ARE	MNG	681.24
ARE	MNE	19.86
ARE	MOZ	206.14
ARE	NZL	599.24
ARE	PRT	1161.32
ARE	RUS	690.70
ARE	RWA	8179.80
ARE	SEN	385.06
ARE	SRB	62.68
ARE	SYC	306.40
ARE	SVN	477.86
ARE	LKA	461.52
ARE	TJK	600.94
ARE	TON	11.50
ARE	TUR	220.28
ARE	TKM	2810.66
ARE	GBR	382.48
ARE	USA	344.16
ARE	URY	1087.30

ARE	YEM	202.66
GBR	AFG	273.20
GBR	DZA	347.12
GBR	ARM	47.78
GBR	AZE	35.74
GBR	BHR	742.34
GBR	BGD	140.60
GBR	BEL	406.48
GBR	BWA	5.98
GBR	BRN	2.97
GBR	BDI	8.96
GBR	COM	24.20
GBR	CYP	66.22
GBR	ERI	67.65
GBR	ETH	182.40
GBR	GAB	9.40
GBR	GMB	4.42
GBR	GEO	92.68
GBR	GHA	54.08
GBR	HKG	1345.88
GBR	IND	6255.80
GBR	IRN	4416.85
GBR	JPN	24393.90
GBR	JOR	201.24
GBR	KEN	1202.45
GBR	KOR	9730.34
GBR	KWT	740.96
GBR	KGZ	9.26
GBR	LBN	82.16
GBR	LBY	416.72
GBR	MWI	18.82
GBR	MYS	45.70
GBR	MDV	758.80
GBR	MLI	124.86
GBR	MLT	17.16
GBR	MUS	9.56
GBR	MEX	70.76
GBR	MMR	59.36
GBR	NPL	12.82
GBR	NLD	32.90
GBR	NZL	338.12
GBR	NIC	357.94
GBR	NOR	230.10
GBR	PAK	2621.64
GBR	PAN	2381.20
GBR	POL	498.24
GBR	ROU	541.78
GBR	RWA	325.92
GBR	WSM	30.90
GBR	SAU	0.08
GBR	SEN	1574.76
GBR	SLE	24.16
GBR	SVK	3036.52
GBR	ESP	487.56
GBR	KNA	204.44
GBR	SUR	428.16
GBR	SWE	2.02
GBR	SYR	905.12
GBR	TJK	144.74
GBR	THA	364.06
GBR	TLS	5097.32
GBR	UKR	209.56
GBR	USA	448.56

GBR	URY	594.92
GBR	MKD	944.74
GBR	ZMB	176.68
GBR	ZWE	28.90
USA	ALB	35.92
USA	ATG	31.93
USA	ARG	329.46
USA	ARM	67.40
USA	AUS	4785.24
USA	AUT	2450.58
USA	AZE	391.68
USA	BHS	13.55
USA	BHR	245.10
USA	BGD	163.20
USA	BRB	87.76
USA	BLR	145.16
USA	BEL	21680.10
USA	BLZ	10.32
USA	BEN	39.28
USA	BOL	16.42
USA	BWA	58.26
USA	BRA	1461.78
USA	BRN	62.33
USA	BGR	395.12
USA	BFA	9.98
USA	BDI	6.56
USA	KHM	13.70
USA	CMR	46.96
USA	CAN	8585.10
USA	CPV	10.18
USA	CAF	0.30
USA	CHL	265.52
USA	CHN	5626.90
USA	COL	213.46
USA	COM	0.46
USA	COD	20.90
USA	CRI	79.76
USA	CIV	163.16
USA	HRV	365.94
USA	CYP	580.18
USA	CZE	2181.58
USA	DNK	4565.14
USA	DOM	10.10
USA	ECU	65.00
USA	EGY	346.34
USA	ERI	11.63
USA	EST	308.10
USA	ETH	108.72
USA	FJI	8.80
USA	FIN	2868.52
USA	FRA	37737.00
USA	GAB	32.98
USA	GMB	20.40
USA	GEO	104.40
USA	DEU	47632.20
USA	GHA	341.34
USA	GRC	2242.82
USA	GRD	16.04
USA	GUY	39.96
USA	HKG	5031.80
USA	HUN	1760.56
USA	ISL	284.92
USA	IND	4776.14

USA	IDN	603.84
USA	IRN	885.93
USA	IRL	28549.90
USA	ISR	2491.60
USA	ITA	16117.10
USA	JAM	122.14
USA	JPN	6978.36
USA	JOR	249.90
USA	KAZ	443.26
USA	KEN	378.77
USA	KOR	3240.52
USA	KGZ	5.70
USA	LVA	178.54
USA	LBN	296.00
USA	LSO	0.88
USA	LBY	259.00
USA	LTU	432.16
USA	LUX	357.28
USA	MDG	44.88
USA	MWI	18.48
USA	MYS	56.36
USA	MDV	1794.74
USA	MLI	17.20
USA	MLT	14.76
USA	MRT	459.96
USA	MUS	67.64
USA	MEX	91.96
USA	MDA	1800.08
USA	MNG	23.02
USA	MNE	6.84
USA	MOZ	590.18
USA	MMR	18.46
USA	NPL	35.10
USA	NLD	17.17
USA	NZL	25324.80
USA	NIC	746.06
USA	NGA	21.46
USA	NOR	1739.60
USA	OMN	4086.66
USA	PAK	338.44
USA	PAN	599.86
USA	PNG	26.48
USA	PRY	14.34
USA	PER	28.74
USA	PHL	100.88
USA	POL	432.86
USA	PRT	3527.16
USA	QAT	2550.98
USA	ROU	481.04
USA	RUS	1128.52
USA	RWA	3577.12
USA	WSM	7.70
USA	STP	1.10
USA	SAU	0.06
USA	SEN	3142.24
USA	SRB	120.50
USA	SYC	159.68
USA	SLE	35.32
USA	SVK	3750.88
USA	SVN	613.66
USA	SLB	347.78
USA	ESP	3699.24
USA	LKA	18536.20

USA	KNA	268.34
USA	LCA	12.70
USA	VCT	33.42
USA	SDN	18.92
USA	SUR	338.54
USA	SWZ	4.64
USA	SWE	5.74
USA	CHE	8305.06
USA	SYR	6997.22
USA	THA	135.38
USA	TLS	1263.96
USA	TGO	0.15
USA	TON	8.80
USA	TTO	0.28
USA	TUN	202.26
USA	TUR	284.62
USA	TKM	4620.26
USA	UKR	102.40
USA	ARE	589.70
USA	GBR	6139.08
USA	URY	56138.60
USA	UZB	35.26
USA	VEN	0.90
USA	VNM	304.50
USA	YEM	199.70
USA	MKD	141.42
USA	ZMB	200.80
USA	ZWE	78.98
URY	AFG	18.80
URY	ALB	33.92
URY	DZA	1157.46
URY	ATG	288.27
URY	ARG	3972.10
URY	ARM	108.06
URY	AUS	16807.50
URY	AUT	4143.90
URY	AZE	174.54
URY	BHS	2679.95
URY	BHR	255.63
URY	BGD	394.72
URY	BRB	541.90
URY	BLR	250.86
URY	BEL	19466.10
URY	BLZ	199.92
URY	BEN	20.00
URY	BOL	328.36
URY	BIH	121.62
URY	BWA	38.60
URY	BRA	16856.40
URY	BRN	190.37
URY	BGR	369.08
URY	BFA	33.16
URY	BDI	5.06
URY	KHM	21.54
URY	CMR	121.94
URY	CAN	209724.00
URY	CPV	23.18
URY	CAF	1.15
URY	CHL	4704.40
URY	CHN	51255.30
URY	COL	6108.84
URY	COD	68.15
URY	CRI	4066.38

URY	CIV	136.44
URY	HRV	398.72
URY	CYP	128.34
URY	CZE	2094.32
URY	DNK	2514.14
URY	DOM	58.20
URY	ECU	1985.38
URY	EGY	1715.92
URY	SLV	1752.30
URY	ERI	36.30
URY	EST	215.50
URY	ETH	305.16
URY	FJI	62.78
URY	FIN	2399.58
URY	FRA	29414.20
URY	GAB	74.38
URY	GMB	22.14
URY	GEO	136.72
URY	DEU	54046.80
URY	GHA	368.44
URY	GRC	1868.42
URY	GRD	108.56
URY	GTM	3549.34
URY	GUY	222.10
URY	HND	1814.38
URY	HKG	16712.50
URY	HUN	1290.64
URY	ISL	546.48
URY	IND	9436.50
URY	IDN	3737.50
URY	IRL	9053.30
URY	ISR	9958.26
URY	ITA	13069.60
URY	JAM	1961.76
URY	JPN	66179.10
URY	JOR	533.74
URY	KAZ	989.02
URY	KEN	522.67
URY	KIR	2.00
URY	KOR	31166.20
URY	KGZ	70.60
URY	LVA	112.12
URY	LBN	485.20
URY	LSO	5.90
URY	LBY	84.67
URY	LTU	349.94
URY	LUX	595.84
URY	MDG	53.84
URY	MWI	69.86
URY	MDV	15048.00
URY	MLI	13.86
URY	MLT	70.84
URY	MRT	238.14
URY	MUS	49.96
URY	MEX	71.86
URY	MDA	121340.00
URY	MNG	37.48
URY	MNE	40.88
URY	MOZ	994.86
URY	MMR	70.80
URY	NPL	29.62
URY	NLD	21.57
URY	NZL	27112.80

URY	NIC	2764.64
URY	NER	756.04
URY	NGA	67.56
URY	NOR	2344.97
URY	OMN	2917.76
URY	PAK	500.18
URY	PAN	1707.64
URY	PNG	1413.23
URY	PRY	99.54
URY	PER	213.26
URY	PHL	2381.94
URY	POL	8798.96
URY	PRT	2504.48
URY	QAT	1157.46
URY	ROU	897.52
URY	RUS	956.58
URY	RWA	5307.04
URY	WSM	12.70
URY	STP	22.52
URY	SAU	0.14
URY	SEN	8713.40
URY	SRB	107.52
URY	SYC	313.08
URY	SLE	9.00
URY	SVK	25021.90
URY	SVN	511.62
URY	SLB	269.46
URY	ESP	4653.58
URY	LKA	10172.30
URY	KNA	225.28
URY	LCA	128.46
URY	VCT	214.46
URY	SDN	81.62
URY	SUR	66.48
URY	SWZ	287.52
URY	SWE	6.26
URY	CHE	3925.86
URY	SYR	12020.40
URY	TJK	276.64
URY	THA	113.36
URY	TLS	8291.32
URY	TGO	0.45
URY	TON	15.08
URY	TTO	10.70
URY	TUN	1651.86
URY	TUR	407.72
URY	TKM	5600.56
URY	UKR	90.24
URY	ARE	810.80
URY	GBR	7915.72
URY	USA	44990.90
URY	UZB	279.14
URY	VEN	3.00
URY	VNM	6017.26
URY	YEM	918.64
URY	MKD	244.48
URY	ZMB	46.48
URY	ZWE	108.70
UZB	ARG	345.08
UZB	BRA	544.54
UZB	CAN	79.10
UZB	CPV	1.62
UZB	CHL	97.72

UZB	CHN	129.56
UZB	FRA	30.38
UZB	DEU	161.88
UZB	IRN	44.14
UZB	ISR	38.50
UZB	ITA	96.74
UZB	JPN	26.98
UZB	MDA	137.68
UZB	NZL	59.82
UZB	PER	63.50
UZB	PHL	27.30
UZB	RWA	82.50
UZB	LKA	113.72
UZB	SYR	32.54
UZB	TLS	49.32
UZB	USA	94.04
UZB	URY	536.26
UZB	VNM	49.30
VUT	AFG	82.80
VUT	AZE	45.64
VUT	BGD	280.44
VUT	KAZ	285.88
VUT	KGZ	67.42
VUT	RWA	950.56
VEN	AUS	1.90
VEN	BEL	1.45
VEN	CRI	0.05
VEN	FJI	3.20
VEN	FRA	1.10
VEN	DEU	0.80
VEN	HKG	0.85
VEN	IND	0.75
VEN	JPN	1.55
VEN	KIR	0.10
VEN	MDV	0.30
VEN	NZL	0.70
VEN	NIC	0.70
VEN	PRY	0.35
VEN	POL	2.45
VEN	SVK	1.70
VEN	ZAF	0.30
VEN	ESP	0.10
VEN	TLS	0.20
VEN	GBR	0.10
VEN	USA	0.30
VEN	URY	0.30
VNM	BHS	33.45
VNM	BLZ	2.02
VNM	BOL	32.54
VNM	BRA	711.43
VNM	CAN	730.20
VNM	CHL	425.70
VNM	CHN	182.40
VNM	COL	1172.44
VNM	CRI	449.64
VNM	CIV	107.54
VNM	DOM	2.18
VNM	ECU	628.16
VNM	SLV	171.00
VNM	DEU	443.50
VNM	GRD	2.32
VNM	GUY	10.94
VNM	HND	88.18

VNM	ITA	163.20
VNM	JAM	320.82
VNM	JPN	175.13
VNM	MDA	850.76
VNM	NZL	1035.30
VNM	NER	193.32
VNM	PNG	171.18
VNM	PER	65.10
VNM	PHL	461.44
VNM	ESP	6407.10
VNM	LKA	738.20
VNM	SDN	1.44
VNM	TUN	267.96
VNM	USA	641.48
VNM	URY	31815.90
VNM	UZB	296.94
YEM	AUS	2668.28
YEM	BEL	495.16
YEM	KHM	433.18
YEM	CAN	275.20
YEM	CPV	2.42
YEM	CHN	2557.94
YEM	CIV	59.66
YEM	FRA	589.94
YEM	DEU	1035.84
YEM	GHA	36.72
YEM	HKG	379.02
YEM	IDN	622.28
YEM	ITA	417.72
YEM	JPN	4582.68
YEM	KOR	615.08
YEM	MDV	1042.06
YEM	MDA	148.70
YEM	NZL	599.16
YEM	NGA	8.54
YEM	PRY	3.44
YEM	POL	649.88
YEM	RWA	245.58
YEM	SAU	0.48
YEM	SRB	38.76
YEM	SVK	1607.42
YEM	LKA	338.80
YEM	TLS	568.28
YEM	TGO	4.85
YEM	USA	906.44
YEM	URY	5039.44
YEM	VEN	0.45
MKD	AUS	21.16
MKD	CHN	1408.40
MKD	DJI	13.68
MKD	EGY	25.30
MKD	ERI	6.25
MKD	FRA	22.90
MKD	IND	879.60
MKD	IDN	14.12
MKD	JPN	197.40
MKD	KOR	215.70
MKD	KWT	105.56
MKD	MDV	33.42
MKD	NIC	31.64
MKD	POL	12.20
MKD	SEN	115.04
MKD	SVK	101.74

MKD	ESP	91.10
MKD	SYR	185.02
MKD	TLS	1210.52
MKD	GBR	219.60
MKD	USA	83.14
MKD	URY	174.16
ZMB	BEL	30.24
ZMB	BWA	14.66
ZMB	BDI	8.02
ZMB	CHN	106.74
ZMB	COD	125.00
ZMB	EGY	78.82
ZMB	FRA	27.60
ZMB	IND	52.62
ZMB	JPN	18.72
ZMB	KEN	17.88
ZMB	KOR	27.00
ZMB	MYS	44.52
ZMB	MDV	26.50
ZMB	NPL	14.64
ZMB	NZL	42.82
ZMB	PAN	19.98
ZMB	SEN	60.84
ZMB	ESP	380.48
ZMB	SYR	856.76
ZMB	THA	89.76
ZMB	TLS	113.92
ZMB	GBR	29.04
ZMB	USA	189.88
ZMB	URY	13.60
ZMB	ZWE	269.25
ZWE	BEL	30.98
ZWE	BWA	142.30
ZWE	BGR	18.43
ZWE	CHN	75.25
ZWE	COD	263.05
ZWE	FRA	29.68
ZWE	DEU	57.20
ZWE	IDN	14.85
ZWE	ITA	33.28
ZWE	JPN	24.05
ZWE	LSO	0.88
ZWE	MYS	54.58
ZWE	MMR	214.15
ZWE	NPL	16.78
ZWE	NZL	280.45
ZWE	RWA	21.38
ZWE	SVK	13.95
ZWE	ESP	872.90
ZWE	LKA	16.78
ZWE	SWE	3.28
ZWE	SYR	104.35
ZWE	TLS	17.98
ZWE	GBR	16.70
ZWE	USA	179.10
ZWE	URY	100.65
ZWE	ZMB	480.58
