# Supplementary material for: Impact of the Topology of Global Macroeconomic Network on the Spreading of Economic Crises
Source: PLoS One. 2011 Mar 31;6(3):e18443. doi: 10.1371/journal.pone.0018443 (PMC3069097; doi:10.1371/journal.pone.0018443)
Supplement: Table S2 — List of all the avalanche relations between non-connected countries. (PDF) [file pone.0018443.s016.pdf]

Crisis Origin	Collapsed country
China	Kuwait
China	Liberia
China	Myanmar
Germany	Liberia
Germany	Montenegro
Germany	Turkmenistan
Hong Kong	Liberia
Hong Kong	Myanmar
Indonesia	Liberia
Indonesia	Myanmar
Indonesia	Panama
Japan	Angola
Japan	Liberia
Japan	Myanmar
Malaysia	Liberia
Malaysia	Myanmar
Malaysia	Panama
Russia	Montenegro
Russia	Turkmenistan
Singapore	Myanmar
USA	Angola
USA	Kuwait
USA	Liberia
USA	Myanmar
