# Supplementary material for: Impact of the Topology of Global Macroeconomic Network on the Spreading of Economic Crises
Source: PLoS One. 2011 Mar 31;6(3):e18443. doi: 10.1371/journal.pone.0018443 (PMC3069097; doi:10.1371/journal.pone.0018443)
Supplement: Text S1 — Supporting Information. (PDF) [file pone.0018443.s017.pdf]

Supplementary Information on

"Impact of the topology of global macroeconomic network on the spreading of economic crises"

by Kyu-Min Lee, Jae-Suk Yang, Gunn Kim, Jae-Sung Lee, Kwang-Il Goh, In-Mook Kim.

Text S1

Sections

S1. Crisis spreading model
S2. Trade volume-GDP profile (TGP) analysis
S3. Avalanches affecting non-trading countries.
S4. Spanning tree representation of the GMN.
S5. The avalanche network.
S6. Randomization properties of GSNs
S7. Continental clustering: Intra-continental linkage analysis.
S8. Avalanche profiles in randomized networks.
S9. Rich-club effect in the randomized networks.
S10. Robustness of the global picture I: Modified crisis spreading models.
S11. Robustness of the global picture II: Impact of fiscal soundness conditions of countries.
S12. Robustness of the global picture III: Crisis spreading dynamics for different f/t.
S13. Supplementary references.

S1. Crisis spreading model

The crisis spreading model introduced in the text is run for each of 175 countries as the initiation country to obtain the avalanche size distribution $P(A)$. The flowchart of the algorithm for the crisis spreading procedure starting from a single country's collapse is depicted in Fig. S1.

S2. Trade volume-GDP profile (TGP) analysis

In the text, we introduced the TGP analysis. The trade pattern of each country can be efficiently visualized in the TGP, with which one could disseminate how it can affect the avalanche size. The TGPs of all countries are provided as Fig. S2.

S3. Avalanches affecting non-trading countries.

In the crisis spreading dynamics, we can find situations where even non-trading countries are affected. For instance, as we can see in Fig. S3, China's collapse leads to Myanmar's collapse even though these two countries are not trading with each other. This is possible through a sequence of cascade in which China makes Thailand collapse via a direct trade relation, and in turn Thailand affects Myanmar via a direct link. A full list of these non-trading avalanche relations is given in

Table S2.

S4. Spanning tree representation of the GMN.

To construct the maximum spanning tree of GMN, we use a modified version of the Kruskal algorithm[1] with the trade volume as the link weight. The algorithm proceeds as follows.

i) Sort the links in the descending order of the link weight (trade volume).
ii) Start from the network of all nodes with no links.
iii) Pick the link on the top of the (remaining) sorted list and putatively put it onto the network.
iv) If the addition of this link leads to the generation of a loop in the network, this link is discarded. Otherwise, the link remains added to the network.
v) The used link is removed from the sorted list.
vi) Repeat iii)—v).

In this way, we obtain a spanning tree connecting all 175 countries with 174 links. The MST of GSNs are obtained by the same algorithm.

S5. The avalanche network.

In the text, we found that the avalanche dynamics was constrained by the structure of GMN. To reveal the global picture, the avalanche network is useful. In the avalanche network, if one country *A* can make the other one *B* collapse via an avalanche, an arrow is assigned to connect the two countries from *A* to *B*. In this way, we can construct the network that reveals the large-scale avalanche relationship among countries at a glance. The avalanche network overlaid with the world map is shown in Fig. S4.

By coarse-graining the avalanche relations at the continental level, we can obtain the continental avalanche network, in which nodes are continents and two continents are connected by a directed link if the union of crises starting from one continent can make more than a given fraction (30%) of countries from the other continent collapse (Fig. S5). In this network, each node size means the total GDP value of countries involved in each continent. The thickness of a link is assigned by the fraction of collapsed countries in the target continent.

We divided the globe into eight continental regions, Africa, North America, South America, East Asia, Middle-East Asia, East Europe, West Europe, and Oceania. See Table S1 for each country's continental association.

S6. Randomization properties of GSNs

In order to extract typical properties of GSN, we need to consider a large number of independent network configurations and the ensemble average behavior obtained from them. Upon randomization, GSN reaches a stationary state after around 5×10^5 shufflings are performed (Fig.

S6A). This can also been seen by observing how the degree of overlap between GSN configurations evolves during the randomization steps. We found that the overlap decreases with the randomizations and at around 5x10^5 shufflings the overlap reaches a saturation level of more than 90% difference (Fig. S6B). Therefore we may consider two GSN configurations separated by 5x10^5 random shufflings as sufficiently independent. To perform ensemble average we collected 10^3 such independent GSN configurations.

As can be seen in the Fig S6A, there are intermittent spikes and dips in the sum of avalanche sizes in the GSN as we perform a long sequence of random shufflings. For the spikes, at which the sum instantaneously increases over 700, what happens is that France's or China's avalanche size increases abruptly by about 120. The increase of France's impact is a result of inducing China's and subsequently Germany's collapse by France at these particular configurations. Meanwhile, China's increment is due to whether the Germany is collapsed by China or not. The dips with the sum below 550 occur for configurations for which Japan's avalanche size decreases by about 120, originating from the fact that Japan is no longer able to make Germany collapse in those configurations.

S7. Continental clustering: Intra-continental linkage analysis.

In order to quantitatively assess the degree of continental clustering exhibited by the GMN and the randomized networks, we calculate the fraction of intra-continental links connecting countries within the same continent. Here we consider the same continental division into eight continental regions as we did for the continental avalanche network.

We calculate the fraction of intra-continental links with 10^3 different configurations of the GSN and the GDN, respectively, and compare the results with those of the GMN. The empirical *P*-value is also obtained with these random ensembles, by observing the number of configurations that show the intra-continental link fraction equal to or higher than that of the GMN.

S8. Avalanche profiles in randomized networks.

In the GSN and the GDN, not only avalanche sizes of countries change with respect to the GMN, but also do the avalanche profiles of countries, the composition of sub-processes that make up a total avalanche process.

In the GSN, two opposite changes are present with respect to the GMN. For countries with large avalanche sizes such as USA, Germany, Japan, China, and France, there is a significant increase in the propensity of indirect avalanche, whereas the direct avalanche remains at almost the same level as in the GMN. In contrast, countries with medium avalanche sizes such as Russia, Indonesia, Malaysia, Hong Kong, and Singapore, lose most of indirect avalanche (Fig. S7A).

In the GDN only three countries, USA, Germany, and China, exhibit finite avalanches, and the proportion of indirect avalanche becomes more dominant, whereas the direct avalanche becomes limited compared with the GMN (Fig. S7B).

S9. Rich-club effect in the randomized networks.

Detailed characterization of topological differences between the GMN and two randomized structures GSN and GDN should provide more insight into the structure-dynamics relationships for understanding differences in the randomized structures compared to GMN. One interesting related network-topological concept is the rich-club effect,[2] introduced to quantify the degree of mutual linkage between high-degree vertices in the network. Because the high-degree vertices correspond to big economies in the GMN and therefore contribute crucially to the spreading process of crises, quantifying changes in the rich-club effect for the randomized structures would reveal an important topological information. To this end, we calculated the rich-club coefficient (RCC) $\phi(s)$ as a function of the node strength s, defined by the total sum of trade a country is engaging in, defined by[2]

$$\phi(s) = \frac{L_{>s}}{n_{>s}(n_{>s}-1)},$$

Where $L_{>s}$ is the number of directed links between vertices with strength larger than s, with $n_{>s}$ being the number of vertices with strength larger than s. We calculated RCCs and compared them for the GMN and two randomized structures GSN and GDN (Fig. S8). The result shows that compared to original network, two randomized structures exhibit a systematic increase of RCC, which suggests that the disentanglement of community structure of the international trades promotes the mutual linkage between big economies. Moreover, the change in RCC is much larger for GDN than in GSN, showing that the more we liberate the community structure by randomizing the trade network, the higher degree of rich-club effect develops. This increased level of rich-club connections may in part account for the differences in crisis spreading property of the randomized structures and that of original network, such as the decrease in the frequency but the increase of average impact of the crisis avalanche shown in Fig. 10B.

S10. Robustness of the global picture I: Modified crisis spreading models.

In the text, we studied the economic crisis spreading in terms of avalanche dynamics based on trade relations. To gain the global picture that is robust to additional details such as the addition of more economic channels between countries or implementing country-specific economic conditions for crisis spreading, it is critical to establish how robust the results obtained in this paper would be under modifications of the crisis spreading model. In this regard, we have considered a number of modifications to the model studied in the text and studied their global avalanche picture.

First, we check whether the avalanche distribution is robust even if we set the crisis spreads through links with weights given by the nonlinear function of the trade volume. In the modified model, each link weight is scaled by the power of factor α. By this setting, we can consider the crisis propagate through something beyond simple trade relationships such as investments, financial relation, etc. For several values of α in the range $1 < \alpha < 1.5$, we have confirmed that there exists a critical point at which $P(A)$ follows the same power-law behavior as the original model, and overall results for the avalanche profiles and the avalanche network are conserved (Figs. S9A-C). As $\alpha \approx 1.5$, we found that the centralization of link weights towards heavy-trading countries becomes extreme, so that the avalanche size distribution becomes super-critical for any finite values of f/t.

Second, the individual country's tolerance capacity to the crisis can also be dependent on something beyond the GDP. In this respect, we also introduce factor-α scaling to each country's GDP and regard this as a new tolerance capacity. Again the avalanche behavior is robust for $0.9 < \alpha < 1.7$ (Fig. S9D-F). Therefore, on the average, the nonlinear scaling for the economic capacities would not alter the general picture of the crisis spreading dynamics established in this work.

Third, we consider the case in which the crisis spreads when the sum of reduced export and import is used for determining whether or not the neighboring country becomes collapsed, rather than considering export and import separately as in the original model. For this model, we observed that there exists a critical point at which $P(A)$ follows the same power-law behavior as the original model, and overall results for the avalanche profiles and the avalanche network are conserved (Figs. S9G-I).

Fourth, we consider the case in which the collapsed country continuously participates in the crisis spreading process, in contrast with the original model in which once collapsed a country does not participate in further processes. In this new model, when a country induces its neighbor's collapse, there is a perpetual cycle of weight reduction occurs, effectively resulting in the complete reduction of the link weights from these countries. Therefore one might expect that the crisis spreads more easily and broadly. It turns out, however, that this model's resulting avalanche outcomes are identical to those of the original model with f=1, therefore with t=1/7, the critical point is retained and we have exactly the same global picture as the original model.

S11. Robustness of the global picture II: Impact of fiscal soundness conditions of countries.

Practically, the tolerance to crisis can be dependent on individual country's current economic indicator such as current account balance and public- and external debt. Here we apply these indices to our model for investigating the robustness of the main conclusions. To this end, we modify the economic capacity of each country with these indices and run the crisis spreading model under the modified economic capacities over the global economic system.

Specifically, for the debt data, we subtracted 40% (1%) of public (external) debt from the GDP to obtain the modified economic capacity of a country. Therefore, the country running into large debt would have lower threshold. The particular choice of the percentage factor for applying debt condition does not have a significant impact on the conclusion, as long as it does not result in the negative economic capacity for countries with large debt. We obtained the debt data from Central Intelligence Agency World Factbook 2008 (https://www.cia.gov/library/publications/download/download-2008/index.html).

As we can see from Fig. S10, the overall feature is robust compared to original model under these modifications: the power-law-like avalanche size distribution is obtained around f/t = 7 (Figs. S10A and D); the avalanche profile exhibits large portions of indirect avalanches (Figs. S10B and E); and there is a significant intra-continental linkage in the avalanche networks (Figs. S10C and F). One notable difference is that under external debt application, Germany acquires significantly large additional avalanche size, ending up a larger avalanche size than even USA. This can happen because under this setting Germany can induce collapses of France and then China, and

subsequently many more countries connected to these two countries. This specific difference, however, does not alter the global patterns of the crisis spreading processes discussed in the text. In reality, all these factors should get integrated together, and predicting the actual outcomes is far from simple. Nevertheless, as all above modification studies suggest, the global patterns of avalanche dynamics would not be qualitatively different from the main conclusions of this work.

S12. Robustness of the global picture III: Crisis spreading dynamics for different *f/t*.

Finally, we consider the robustness of the conclusions with respect to the model parameter, in particular the parameter ratio *f/t*. As shown in Fig. 2, the avalanche size distribution can take qualitative different form for different value of *f/t*. Therefore, it would be important to check the robustness of the conclusions drawn from the "critical point" *f/t*=7.

First, near the critical point, say 5<*f/t*<9, the avalanche size distribution keeps the approximate power-law form (Fig. S11) and in this range of *f/t,* the overall properties of the crisis spreading model dynamics remain intact (Fig. S12). The avalanche profiles contain significant indirect avalanches; the continental clustering and enhanced intra-continental linkage clearly manifest in the avalanche network. Therefore, the overall conclusions of our model study can be reached unambiguously for any choices of the cascading parameter *f/t* within this rather wide range.

Secondly, further away from the critical point, the avalanche size distribution no longer attains a simple approximate power-law form. Especially, when *f/t* is too small as *f/t* < 3, there is no non-zero avalanche in the model. On the other hand, when *f/t* is too large as *f/t* > 20, there is an excess of global avalanches which affects more than 90% of countries, making a delta-function-type peak at *A*=167 (Figs. S13A and D). Even in those non-power-law regimes, the key properties of the crisis spreading dynamics we identified can be observed. For example, when *f/t*=3, only ten countries exhibit non-zero avalanches, with *A*=8 being the largest avalanche. Even in this weakly-interacting case, one can see that the indirect avalanche occurs significantly (Fig. S13B) and the avalanches are highly constrained within continent (Fig. S13C). On the other extreme, when *f/t*=20, 27 countries are able to initiate global avalanches with *A*=167, spanning the whole globe. Even in this strongly-interacting case, the two key properties of propensity of indirect avalanche (Fig. S13E) and continental clustering (Fig. S13F) can be detected. Specifically, if we discard the delta-function-like peak at *A*=167, the fraction of intra-continental links remains to be strongly enhanced compared to randomized structures, for the whole range of cascade parameters 3 < *f/t* < 30 (Fig. S14). Altogether, we have found that the choice of the parameter ratio *f/t* does not critically affect our main findings and thereby the general conclusion presented for a particular parameter ratio *f/t* =7 in the text.

S13. Supplementary references

1. Cormen TH, Leiserson CE, Rivest RL, Stein C (2001) *Introduction to Algorithms*. 2nd ed. (MIT University Press, Cambridge).
2. Zlatic V, Bianconi G, Garlaschelli D, Rao F, Caldarelli G (2009) On the rich-club effect in dense and weighted networks. Eur Phys J B 67:271-275.
